# The cyclopropylcarbinyl route to γ-silyl carbocations

**DOI:** 10.3762/bjoc.15.170

**Published:** 2019-07-24

**Authors:** Xavier Creary

**Affiliations:** 1 Department of Chemistry and Biochemistry, University of Notre Dame, Notre Dame, IN 45556, USA

**Keywords:** bicyclobutane, carbocation, cyclopropylcarbinyl, rearrangement, silicon

## Abstract

The mesylate derivative of *cis*-1-hydroxymethyl-2-trimethylsilylcyclopropane has been prepared, along with a number of related mesylates and triflates with substituents on the 1-position. These substrates all solvolyze in CD_3_CO_2_D to give products derived from cyclopropylcarbinyl cations that undergo further rearrangement to give 3-trimethylsilylcyclobutyl cations. These 3-trimethylsilylcyclobutyl cations are stabilized by a long-range rear lobe interaction with the γ-trimethylsilyl group. When the substituent is electron-withdrawing (CF_3_, CN, or CO_2_CH_3_), significant amounts of bicyclobutane products are formed. The bicyclobutanes are a result of γ-trimethylsilyl elimination from the cationic intermediate that has an unusually long calculated Si–C bond. The solvolysis chemistry of mesylate and triflate derivatives of *trans*-1-hydroxymethyl-2-trimethylsilylcyclopropane and 1-substituted analogs can be quite different since these substrates do not generally lead to 3-trimethylsilylcyclobutyl cations.

## Introduction

Carbocations, positively charged trivalent carbon compounds and reactive intermediates, have continued to fascinate chemists since the early discoveries of tropylium [1–2] and trityl [3–7] salts. Many of the giants of organic chemistry during the last century contributed heavily to the development of carbocation chemistry. This article will deal with three types of carbocations that have been of intense and fundamental interest over the years, i.e., cyclopropylcarbinyl cations, electron-deficient cations, and silyl substituted carbocations. A brief overview of these types of carbocations is warranted.

**Scheme 1:**
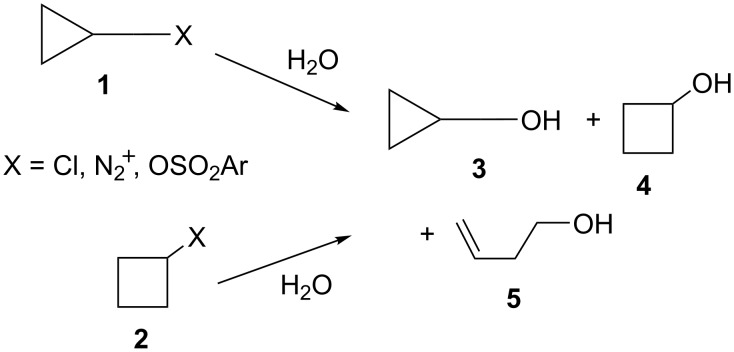
Solvolyses of cyclopropylcarbinyl and cyclobutyl substrates.

**Scheme 2:**
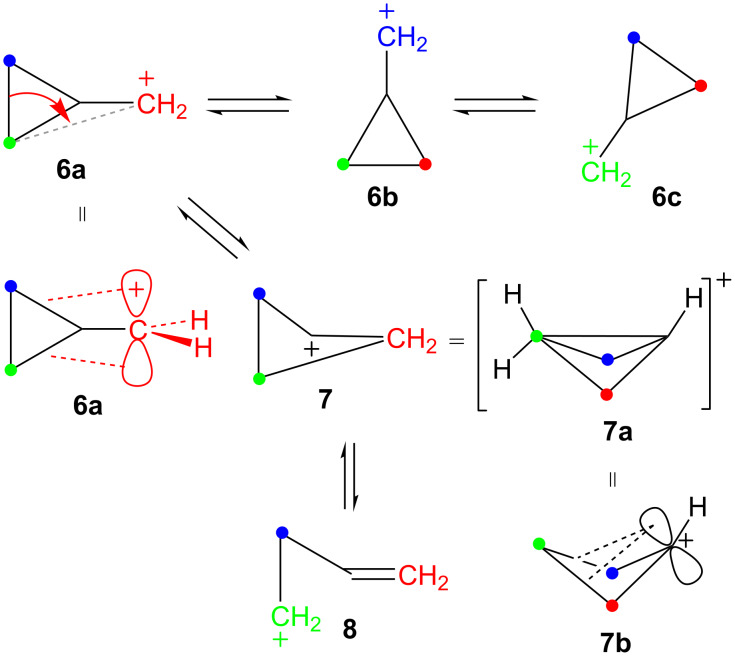
The cyclopropylcarbinyl–cyclobutyl–homoallyl cation manifold.

Cyclopropylcarbinyl cations are an extensively studied system [8–9]. Initial interest was derived from the fact that both cyclopropylcarbinyl and cyclobutyl substrates **1** and **2**, where X represents diazonium ion [10–11], chloride [10], or naphthalenesulfonate [12] leaving groups, reacted in aqueous solvents to give an identical mixture of products **3**, **4**, and **5** (Scheme 1). Additionally, solvolysis rates were far greater than expected for primary and strained secondary systems. To account for these facts, it has been suggested that there are common cationic intermediates in these solvolysis reactions of **1** and **2**. Labelling [13–15], stable ion [16–19], and computational studies [19] implicate the involvement of three degenerate cyclopropylcarbinyl cations, **6a**, **6b**, and **6c**, in equilibrium with cyclobutyl cation **7**, as well as the homoallylic cation **8** (Scheme 2). Cations **6** are stabilized by the cyclopropyl ring and are therefore much more stable than simple primary carbocations. The cyclobutyl cation **7** is also quite stabilized relative to simple secondary carbocations. This cation has been called a “bicyclobutonium” cation, **7a**, which is a nonclassical cation (a cation containing hypercoordinated carbon) that could be derived from protonation of bicyclobutane [20]. Another potential mode of stabilization is by an interaction of the cationic center with the adjacent strained cyclobutyl bonds as in **7b**.

**Figure 1:**
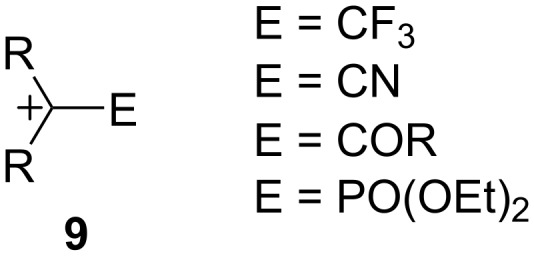
Electron-deficient carbocations.

A second class of carbocations that this article will deal with is the so-called “electron-deficient” carbocation, i.e., carbocations **9** (Figure 1) substituted with electron-withdrawing groups E [21]. Many studies have shown that such cations can indeed be generated and that they can derive stabilization by a variety of mechanisms. Chief among these cations are the α-trifluoromethyl [22–24], α-cyano [22,25–29], α-carbonyl [30–33], and α-phosphoryl [34–35] analogs of **9**. Carbocations of type **9** will be examined in conjunction with the cyclopropylcarbinyl–cyclobutyl manifold.

**Scheme 3:**
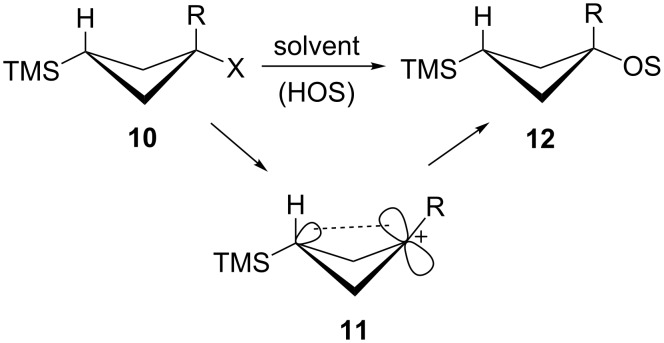
Solvolyses of γ-trimethylsilylcyclobutyl substrates.

The third type of carbocation that will be incorporated into this paper is the trimethylsilyl-substituted carbocation [36–44]. We have been interested in long-range interactions of silicon with both carbene [45–48] and carbocation centers [49–50]. Along these lines, γ-trimethylsilyl cations of general type **11** have been generated under stable-ion [51] as well as solvolytic conditions [52–54]. They are greatly stabilized by the “rear lobe” type of interaction shown involving the γ-trimethylsilyl group. A number of related cations are also stabilized by analogous γ-silyl interactions [55–59], which have also been termed ”percaudal” interactions [56]. Certain carbenes can also be stabilized in a similar fashion [60–61]. Thus substrates of type **10** solvolyze in protic solvents with large rate enhancements (anchimeric assistance) to generate carbocations **11** as reactive intermediates (Scheme 3). These cations **11** capture solvent molecules to give exclusively products **12** with net retention of configuration, a characteristic of carbocations that are stabilized by this type of rear lobe interaction.

**Figure 2:**
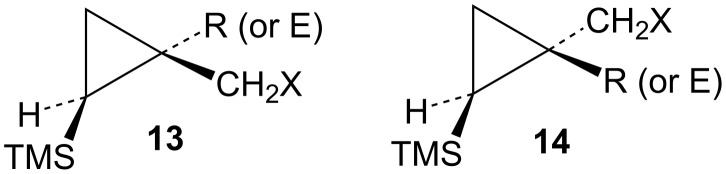
Substrates of interest.

A series of cyclopropylcarbinyl substrates **13** and **14** (Figure 2), where X is a leaving group and R is an electron-donating group and E is an electron-withdrawing group, have now been examined. The goal was to evaluate the cyclopropylcarbinyl to cyclobutyl cation rearrangement. Can these substrates lead to γ-trimethylsilyl-substituted cyclobutyl cations **11** and what are the fates of such carbocations? Answers to these questions were sought.

## Results and Discussion

### Phenyl-substituted systems

**Scheme 4:**
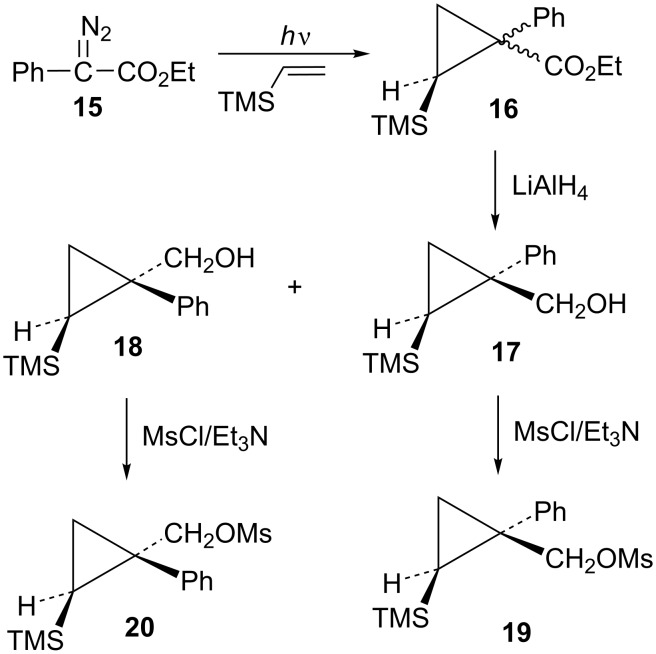
Synthesis of mesylates **19** and **20**.

The first compounds to be examined were the mesylates **19** and **20**. These substrates were prepared as shown in Scheme 4. Irradiation of ethyl 2-diazo-2-phenylacetate (**15**) in vinyltrimethylsilane as solvent gave an isomeric mixture of esters **16**. Subsequent reduction with lithium aluminum hydride gave a mixture of alcohols **17** and **18**, which could be readily separated by silica gel chromatography. The assignment of stereochemistry of these isomers was based on shielding effects in both ^1^H and ^13^C NMR spectra. For example, the trimethylsilyl singlet in **18** appears at δ −0.30 (shielded by the *cis*-phenyl group), while the trimethylsilyl singlet in **17** appears at δ 0.14 (deshielded by the *trans*-phenyl group). Such effects are in complete agreement with calculated shifts based on B3LYP/6-31G* calculated structures of **17** and **18**. Additionally, nOe studies on **17** confirm the stereochemical assignment. Conversion to mesylates **19** and **20** using mesyl chloride and triethylamine was straightforward.

**Scheme 5:**
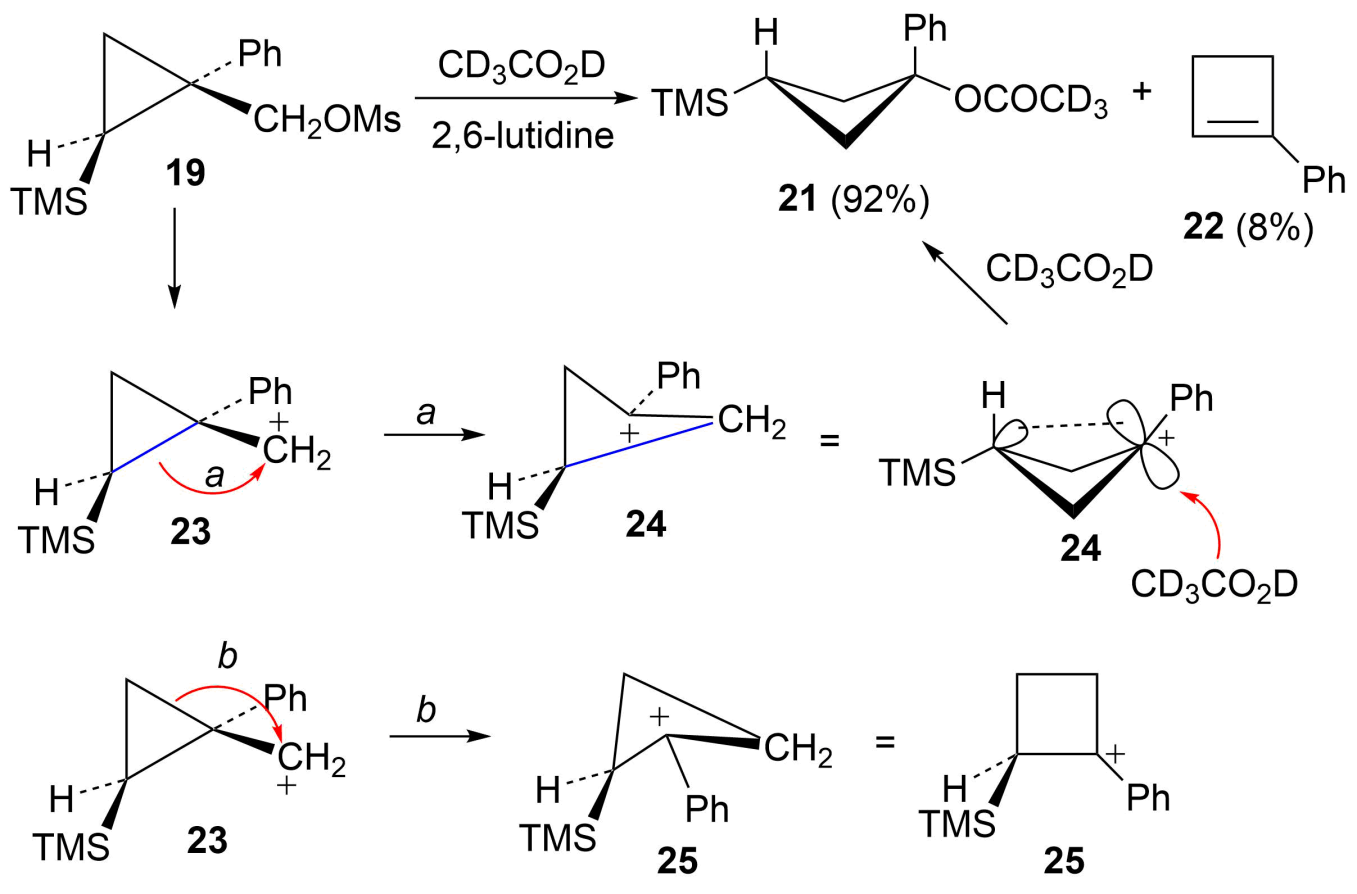
Reaction of mesylate **19** in CD_3_CO_2_D.

Mesylate **19** reacts readily in CD_3_CO_2_D at 20 °C (Table 1) to give the substituted cyclobutyl acetate **21** (92%) as the major product along with 8% of the alkene **22**. It is proposed (Scheme 5) that these products arise from stepwise formation of the cyclopropylcarbinyl cation **23**. This cation can rearrange via migration of bond *a* to give the cyclobutyl cation **24**. The *cis*-nature of the phenyl group and the hydrogen in cation **23** necessarily results in the formation of the γ-silyl-stabilized cation **24**. This cation is the source of the acetate **21**. Alternatively, cation **23** can rearrange by migration of the *b* bond of the cyclopropane. This leads to the β-silylcyclobutyl cation **25**, which can subsequently desilylate to give the minor product, the alkene **22**. Interestingly, formation of the γ-silyl cation **24** is preferred over the β-silyl cation **25**.

**Scheme 6:**
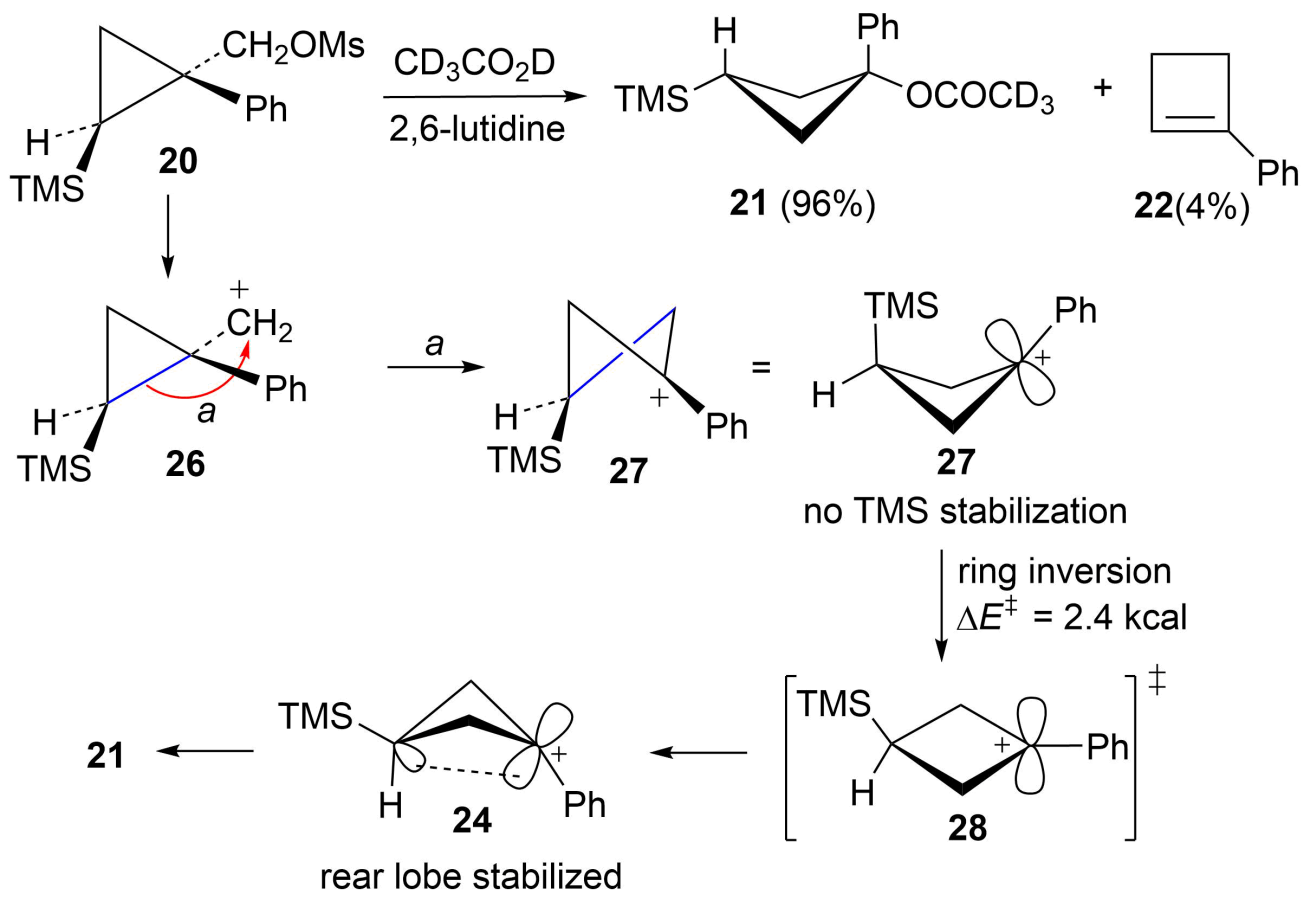
Reaction of mesylate **20** in CD_3_CO_2_D.

**Figure 3:**
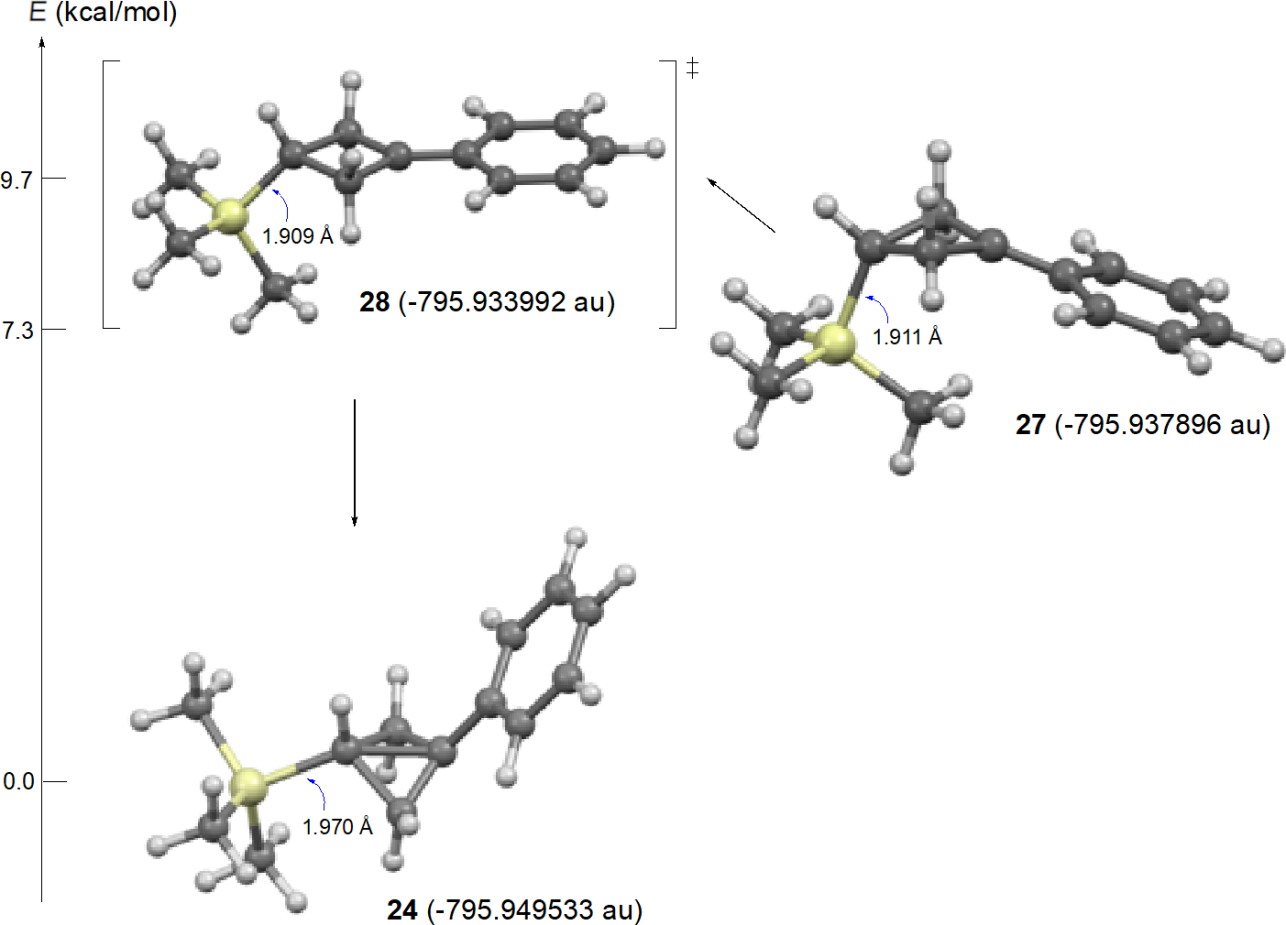
M062X/6-311+G** calculated structures and relative energies of cations **24**, **27**, and transition state **28**.

Reaction of the isomeric mesylate **20** in CD_3_CO_2_D gives the same rearranged products **21** and **22**. These products are accounted for mechanistically in Scheme 6. The initially formed cyclopropylcarbinyl cation **26** rearranges by migration of the *a* bond of the cyclopropane to give the cyclobutyl cation **27**. This cation **27** is different from the γ-silyl-stabilized cation **24** in that the *cis*-nature of the phenyl and TMS groups in **26** requires that these groups are closer to each other in **27**. Shown in Figure 3 are M062X/6-311+G** calculated structures and energies of cations **27** and **24**, which are distinct energy minima, along with the transition state **28** which connects these two cations. Cation **27** derives most of its stabilization from the phenyl group, while the TMS group in the 3-position provides no cross-ring stabilization. The calculated barrier for ring inversion of **27** to give the lower energy rear lobe stabilized γ-trimethylsilyl cation **24** is only 2.4 kcal/mol. Calculations at the B3LYP/6-31G*, B3LYP/6-311+G**, MP2/6-31G*, and the MP2/6-311+G** levels lead to the same conclusions, i.e., cations **24** and **27** are distinct energy minima with a very low barrier for conversion of **27** to **24**. Therefore, formation of **27** under solvolytic conditions should readily yield **24**, and subsequently the substitution product **21**. The small amount (4%) of elimination product **22** is a result of rearrangement of **26** to the β-trimethylsilyl cation **25** as described in Scheme 5.

### Unsubstituted and methyl-substituted systems

**Scheme 7:**
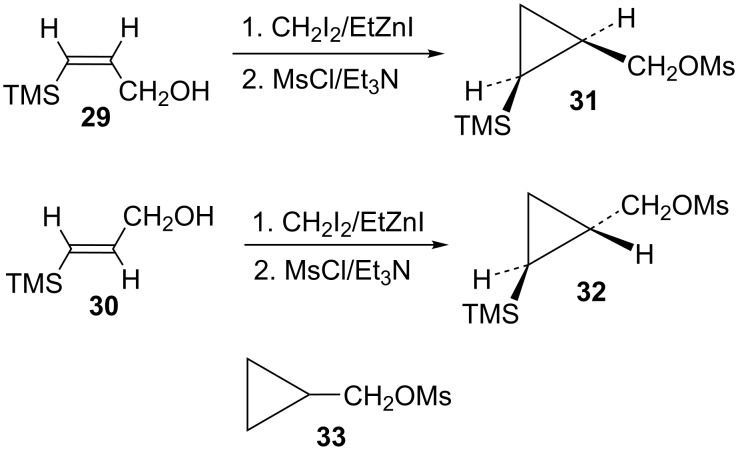
Synthesis of mesylates **31** and **32**.

Attention was next turned to potential γ-trimethylsilylcyclobutyl cation systems lacking phenyl stabilization. Thus pure *Z-* and *E*-alcohols **29** and **30** were each cyclopropanated under Simmons–Smith conditions, and the resultant stereochemically pure alcohols were converted to mesylates **31** and **32**, respectively (Scheme 7). For rate comparisons, cyclopropylcarbinyl mesylate **33** [62–63] was also prepared.

**Scheme 8:**
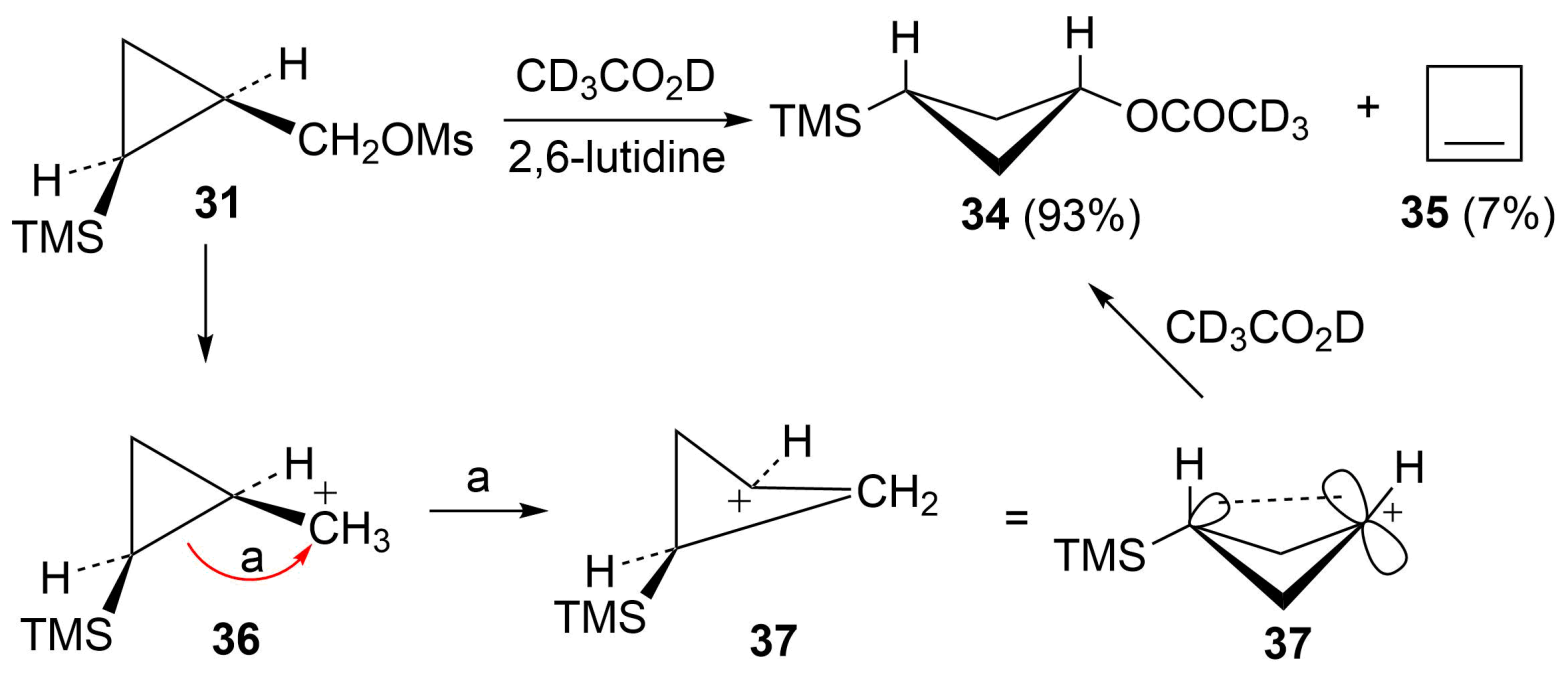
Reaction of mesylate **31** in CD_3_CO_2_D.

Mesylate **31** reacted readily in CD_3_CO_2_D to give the *cis*-cyclobutyl acetate **34** as the major product (Scheme 8), along with a small amount of cyclobutene (**35**). The rate of **31** (Table 1) is not substantially enhanced relative to the unsubstituted cyclopropylcarbinyl mesylate (**33**). The small rate enhancement factor of 3.56 is consistent with a small inductive stabilization of the initially formed cationic intermediate. This behavior is completely analogous to that of the phenyl analog **19** and a similar mechanistic pathway is proposed. The initially formed cyclopropylcarbinyl cation **36** rearranges to the γ-silylcyclobutyl cation **37**, the source of the major product **34**. The desilylated product **35** arises from the alternative β-trimethylsilylcyclobutyl cation.

**Scheme 9:**
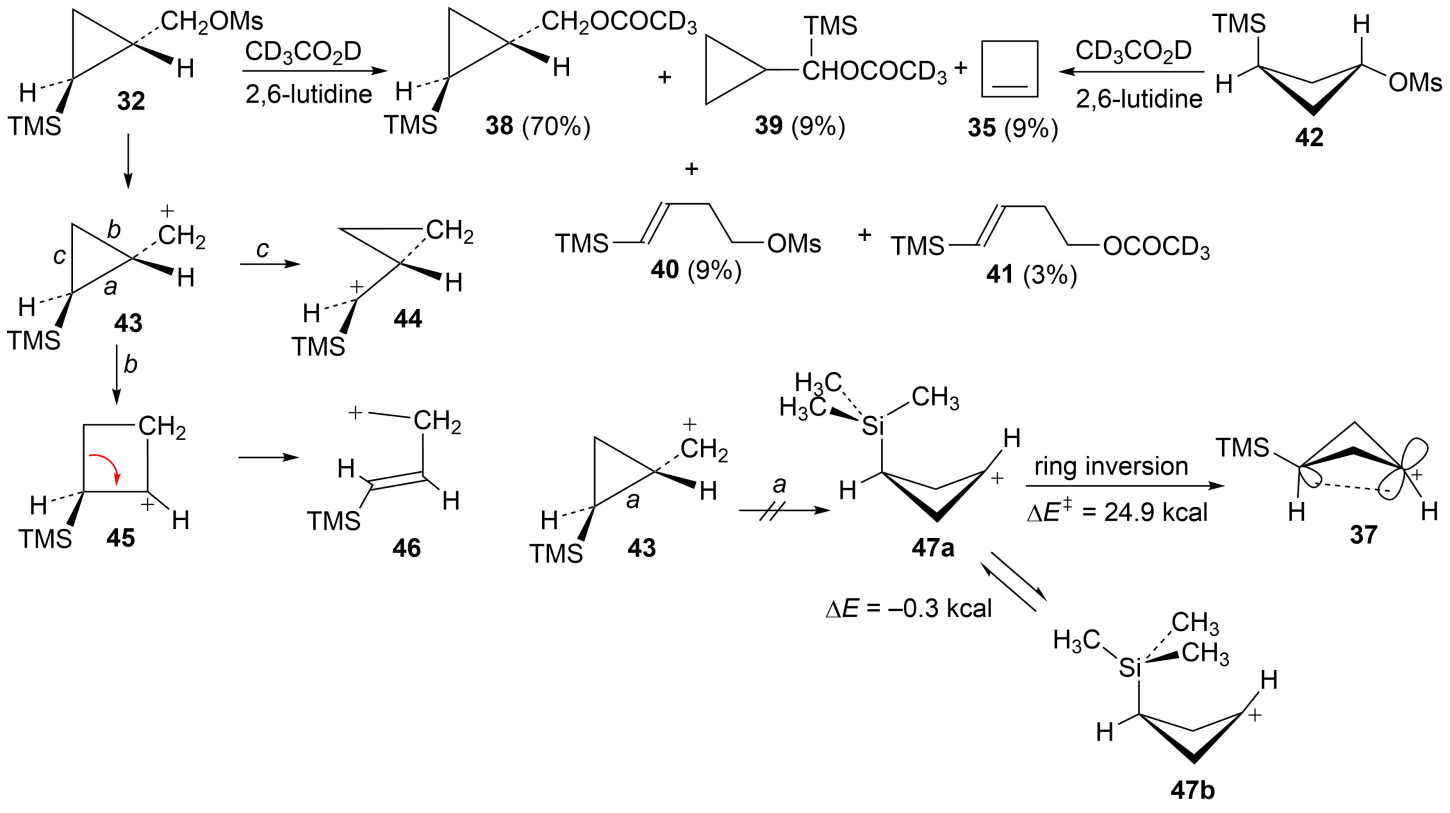
Reaction of mesylate **32** in CD_3_CO_2_D.

The behavior of mesylate **32** is in contrast to that of **31** and the phenyl analog **20**. Five products, **35**, **38**, **39**, **40**, and **41**, are obtained and these products are formed in essentially the identical ratio as seen in our previous study of the *trans*-mesylate **42** [52]. The similarity of products formed from acetolysis of **32** and **42** implies that the same cation rearrangement manifold is involved. Scheme 9 gives a mechanistic rationale for these products. Capture of an unrearranged discrete cyclopropylcarbinyl cation **43** gives the major product **38**, while migration of bond *c* to the cationic center gives rearranged cation **44**, the source of the rearranged acetate **39**. Ring expansion via migration of bond *b* in **43** gives the β-trimethylsilyl-stabilized cyclobutyl cation **45**, and subsequent desilylation provides cyclobutene (**35**). Alternatively, cyclobutyl to homoallylic cation rearrangement leads to the homoallylic products **40** and **41** via internal mesylate return or solvent capture. Of interest is the fact that no product **34** (derived from γ-trimethylsilyl-stabilized cation **37**) is formed. Our previous computational study [52] provided insight into the lack of involvement of cation **37**. This study at the B3LYP/6-31G* level suggested that migration of bond *a* in **43** is not viable since the resultant cation **47** is not an energy minimum at this level, but a transition state. However, a current study at the M062X/6-311+G** level finds that both conformations **47a** and **47b** are energy minima. While **47a** lies 10.8 kcal/mol above **37**, the barrier for inversion of **47a** to **37** is quite large (24.9 kcal/mol). Hence there is no viable route to **37**.

**Scheme 10:**
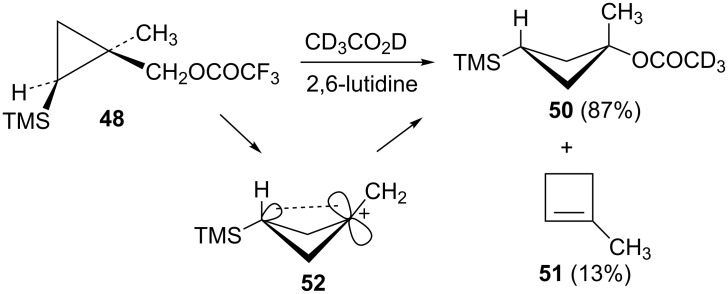
Reaction of trifluoroacetate **48** in CD_3_CO_2_D.

In order to complete the study of substrates **13** with electron-donating groups, the methyl analog **48** was prepared from the corresponding cyclopropylcarbinyl alcohol, which was available from methyl 2-diazopropanoate by a process completely analogous to the synthesis of the phenyl analog **17**. The mesylate derivative was too reactive for rates to be measured and hence the trifluoroacetate derivative **48** was studied. Acetolysis gave the acetate **50** along with a smaller amount of methylcyclobutene (**51**, Scheme 10). This reactivity is completely analogous to that seen in the phenyl and hydrogen analogs **19** and **31**, i.e., a mechanistic scheme involving the γ-trimethylsilyl-stabilized cation **52** is likely.

The isomeric trifluoroacetate **49** (shown in Table 1) gives methylcyclobutene (**51**) (68%) as the major acetolysis product, along with minor products that are identical to those previously reported [52] in solvolysis of the trifluoroacetate derivative of (1*r*,3*r*)-1-methyl-3-(trimethylsilyl)cyclobutanol. As in the case of mesylate **32**, the γ-trimethylsilyl-stabilized cation **52** is apparently not formed from trifluoroacetate **49** due to stereochemical constraints.

**Table 1:** Solvolysis rates for substrates in CD_3_CO_2_D at 20.0 °C.

Compound	*k* (s^−1^)	*k*_rel_ (for ROMs)

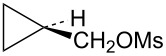 **33**	1.71 × 10^−4^	1.0
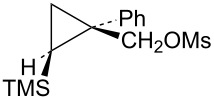 **19**	6.50 × 10^−4^	3.8
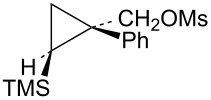 **20**	1.26 × 10^−3^	7.4
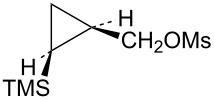 **31**	6.09 × 10^−4^	3.6
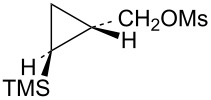 **32**	6.89 × 10^−4^	4.0
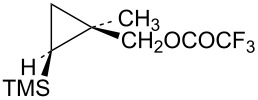 **48**	1.31 × 10^−7a^	76^b,c^
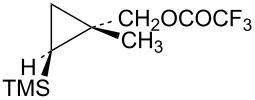 **49**	8.91 × 10^−8a^	52^b,c^

^a^Extrapolated from data at higher temperatures. *k* for **48** at 60.0 °C = 2.58 × 10^−5^ s^−1^; *k* for **48** at 80.0 °C = 2.33 × 10^−4^ s^−1^; *k* for **49** at 60.0 °C = 1.62 × 10^−5^ s^−1^; k for **49** at 80.0 °C = 1.42 × 10^−4^ s^−1^. ^b^Mesylate is too reactive for rate to be measured. ^c^Assuming mesylate reacts 10^5^ faster than trifluoroacetate.

### Systems with electron-withdrawing groups

**Scheme 11:**
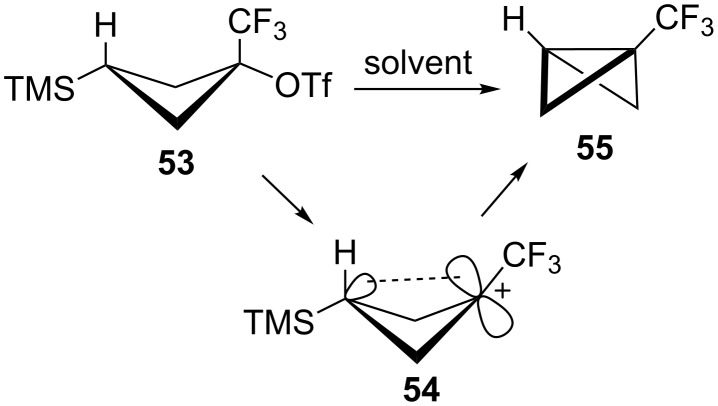
Bicyclobutane formation from a γ-trimethylsilyl cation.

Attention was next turned to cyclopropylcarbinyl systems substituted with electron-withdrawing groups. Previously Tilley and co-workers [55] have examined the triflate **53** and found that this system solvolyzes with rear lobe TMS participation (Scheme 11). The unusual feature in solvolysis of **53** is the formation of the highly strained bicyclobutane **55** as the sole product. It was therefore of interest to see if the cyclopropylcarbinyl to cyclobutyl rearrangement could be used to access the carbocation **54**, and subsequently, bicyclobutane **55**. It was also of interest to see if other bicyclobutanes could be formed if the CF_3_ group were replaced by other electron-withdrawing groups that we have previously examined in carbocation forming reactions.

**Scheme 12:**
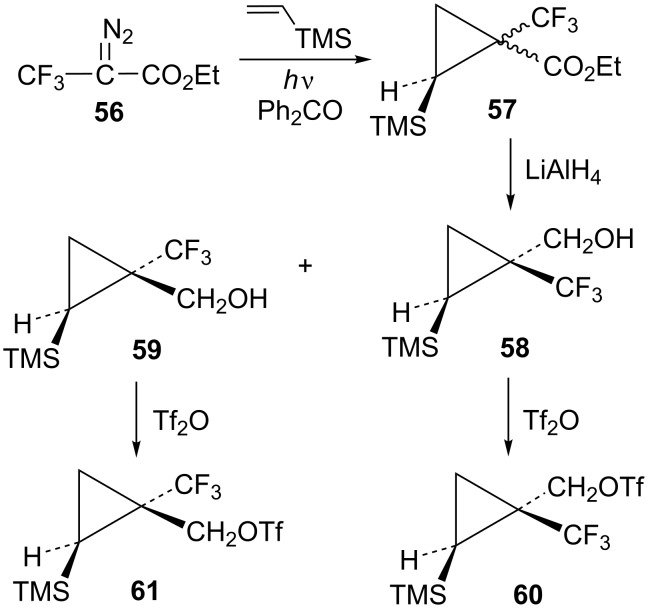
Formation of triflates **60** and **61**.

The requisite trifluoromethyl-substituted cyclopropylcarbinyl systems were prepared by addition of the carbene derived from the diazoester **56** to vinyltrimethylsilane as shown in Scheme 12. Reduction of the ester mixture **57** with lithium aluminum hydride gave a chromatographically separable mixture of alcohols **58** and **59**. Stereochemistry of the alcohol **58** was established by long-range ^19^F coupling to the *cis*-trimethylsilyl group hydrogens (*J*_H-F_ = 0.9 Hz). Long-range ^19^F coupling to the TMS methyl groups of **58** was also observed in the ^13^C NMR spectrum (*J*_C-F_ = 2.1 Hz) [64–65]. This long-range ^19^F coupling is not observed when the CF_3_ group is *trans* to the TMS group in the isomer **59**.

**Scheme 13:**
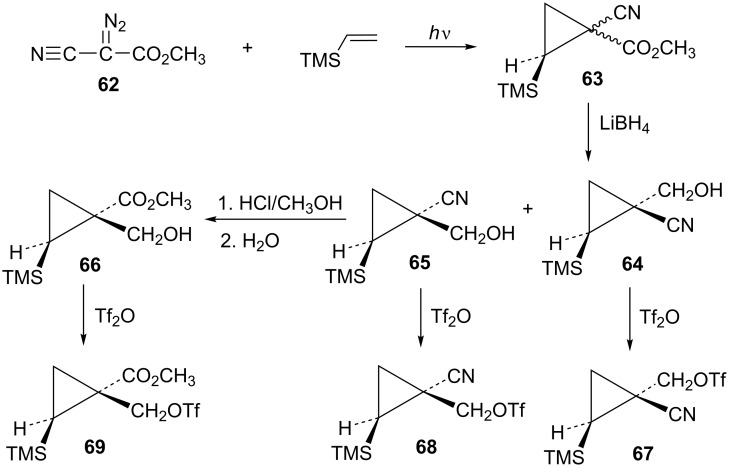
Formation of triflates **67**, **68**, and **69**.

Additional cyclopropylcarbinyl systems containing the electron-withdrawing cyano and carbomethoxy groups were prepared in an analogous fashion as shown in Scheme 13. Carbomethoxycyano carbene addition to vinyltrimethylsilane followed by lithium borohydride reduction of the ester functionality of **63** gave a separable mixture of alcohols **64** and **65**. The stereochemistry of the product **65** was established using nOe studies. Cyano to carbomethoxy conversion in **65** to give alcohol **66** was straightforward. Triflate derivatives **67** and **68** were prepared since analogous mesylate derivatives were relatively unreactive. Triflate **69** was a highly reactive substrate that could only be prepared in about 80% purity. The less reactive mesylate derivative **75** was therefore prepared and used for kinetic studies.

**Scheme 14:**
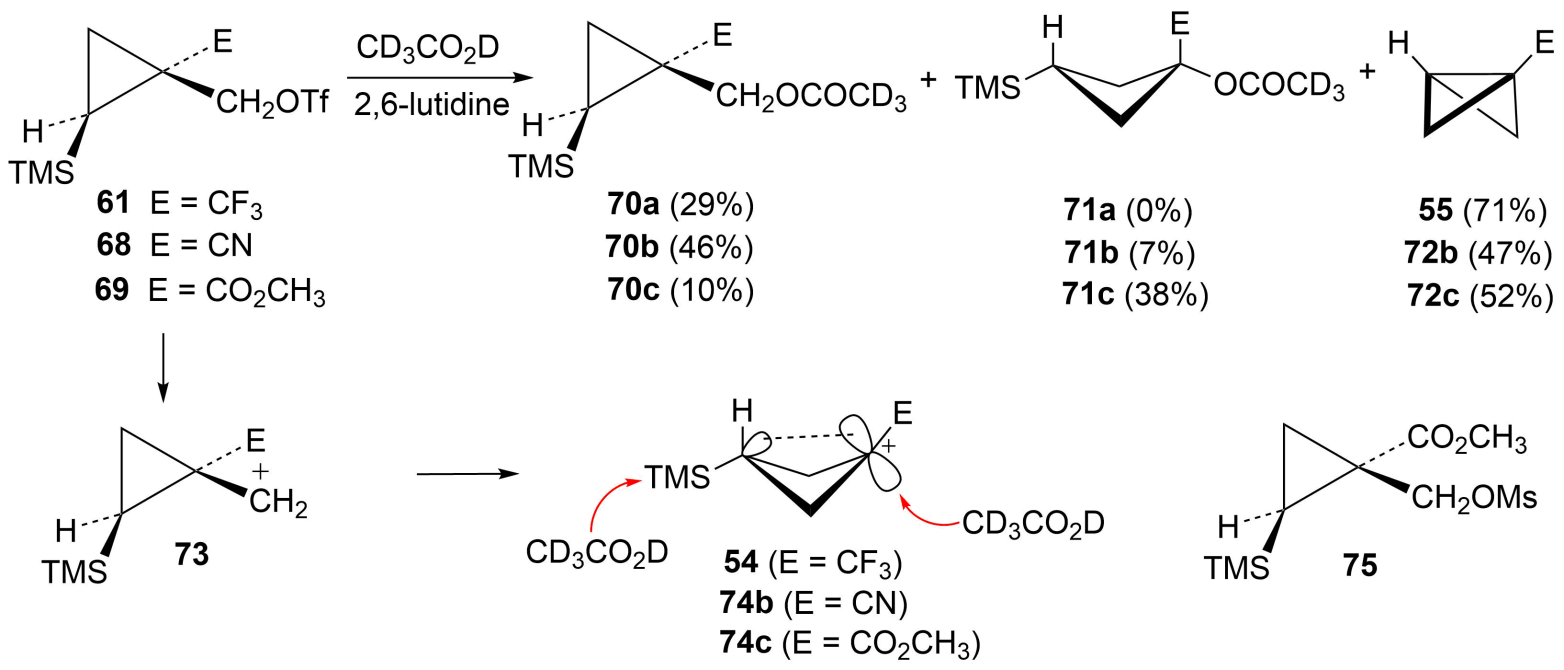
Reactions of substrates with electron-withdrawing groups in CD_3_CO_2_D.

The triflates **61**, **68**, and **69** (with electron-withdrawing groups *trans* to trimethylsilyl) were all solvolyzed in CD_3_CO_2_D and results are shown in Scheme 14. Since the triflate **69** was highly reactive and could not be isolated in pure form, the mesylate derivative **75** was used in kinetic studies that were carried out in the 40–60 °C range. Rates of reaction of mesylate derivatives (Table 2) were all substantially slower than the parent mesylate **33** or the phenyl, methyl, or H analogs. This is attributed to a significant inductive destabilizing β-effect of the group E on the initially formed cation **73**. The triflates all produced significant amounts of bicyclobutane products **55** and **72** along with some unrearranged substitution products **70**. In the cases of **68** and **69**, some rearranged substitution products **71** were also formed. The mesylate **75** gave the same initial products as the triflate **69**. However, the bicyclobutane **72c** formed from mesylate **75** was not completely stable at 40–60 °C, but degraded slowly to a mixture of other products. The bicyclobutanes **55**, **72b**, and **72c** were quite stable in CD_3_CO_2_D at 20 °C, where triflate studies were carried out.

**Table 2:** Solvolysis rates for substrates in CD_3_CO_2_D at 20.0 °C.

Compound	*k* (s^−1^)	*k*_rel_ (for ROMs)

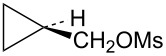 **33**	1.71 × 10^−4^	1.00
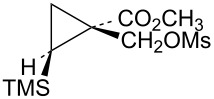 **75**	1.26 × 10^−7a^	7.3 × 10^−4^
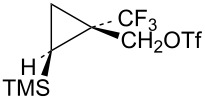 **61**	2.25 × 10^−4^	1.3 × 10^−5b^
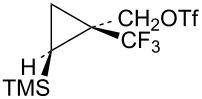 **60**	1.25 × 10^−3^	7.3 × 10^−5b^
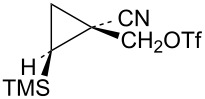 **68**	2.14 × 10^−4^	1.3 × 10^−5b^
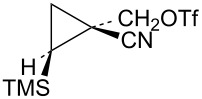 **67**	1.61 × 10^−3^	9.4 × 10^−5b^

^a^Extrapolated from data at higher temperatures. *k* at 40.0 °C = 2.24 × 10^−6^ s^−1^; *k* at 50.0 °C = 8.40 × 10^−6^ s^−1^; *k* at 60.0 °C = 2.85 × 10^−5^ s^−1^. ^b^Assuming triflate reacts 10^5^ faster than mesylate.

**Figure 4:**
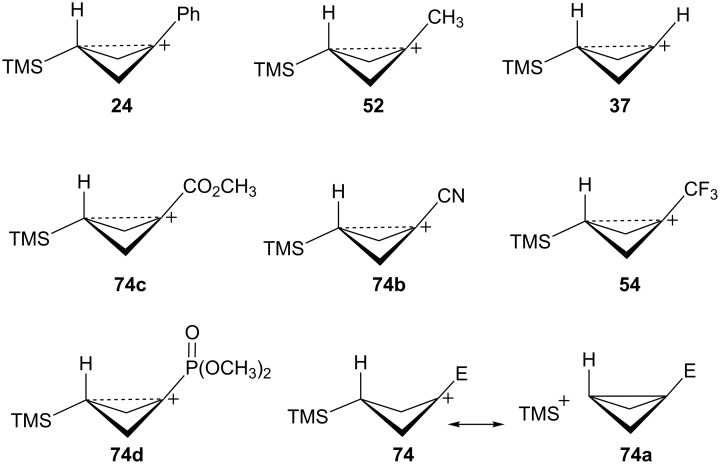
γ-Trimethylsilyl cations.

**Scheme 15:**
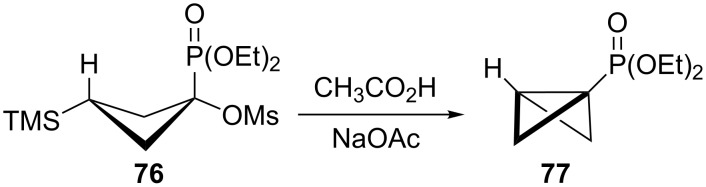
Bicyclobutane formation from mesylate **76** in CH_3_CO_2_H.

The bicyclobutane products **55** and **72** are a result of desilylation of the γ-silyl cations **54** and **74**. Why are bicylobutanes formed from cations **54** and **74** and not from cations **24**, **37**, and **52**, which do not have electron-withdrawing groups? Previous studies have shown that “electron-deficient” cations **9**, where E = COR [66], CN [25], CF_3_ [67], and PO(OEt)_2_ [34], readily eliminate β-hydrogens to form alkenes as major products. They do not readily capture solvent at the cationic center. It is therefore expected that nucleophilic attack at the cationic centers of **54** and **74** will be slowed. Table 3 shows results of calculations on the γ-trimethylsilylcyclobutyl cations shown in Figure 4 at different levels of theory. The presence of the electron-withdrawing group results in an increase in the Si–C3 bond length relative to the cations **24** and **52**. Also, the cross-ring C1–C3 distance is decreased. In the language of resonance theory, these features are in line with increased contributions of form **74a** to the overall structure of the cation. These features suggest more facile nucleophilic attack should occur at silicon, favoring bicyclobutane formation. Also included in Table 3 are calculated bond lengths in the phosphoryl-substituted cation **74d**, which also shows a very long Si–C bond. Preferred trimethylsilyl elimination from this intermediate is in line with the behavior of mesylate **76**, which gives exclusively the bicyclobutane **77** on solvolysis in CH_3_CO_2_H (Scheme 15).

**Table 3:** Calculated bond lengths (Å) of γ-trimethylsilyl cations.

Cation	Bond	B3LYP/ 6-31G*	B3LYP/ 6-311+G**	MP2/ 6-31G*	MP2/ 6-311+G**	M062X/ 6-311+G**

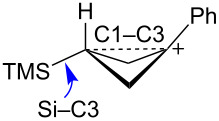 **24**	Si–C3 C1–C3	1.962 1.916	1.959 1.914	1.975 1.760	1.970 1.759	1.970 1.736
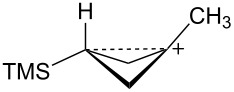 **52**	Si–C3 C1–C3	1.999 1.717	1.994 1.719	1.990 1.665	1.983 1.675	1.984 1.652
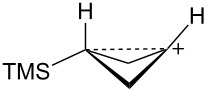 **37**	Si–C3 C1–C3	2.016 1.662	2.013 1.659	2.004 1.636	1.998 1.645	2.000 1.616
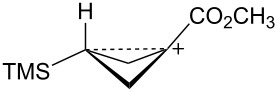 **74c**	Si–C3 C1–C3	2.018 1.658	2.018 1.655	2.009 1.625	2.002 1.632	2.007 1.601
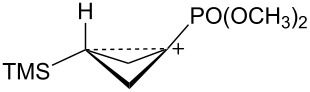 **74d**	Si–C3 C1–C3	2.013 1.663	2.012 1.659	2.008 1.624	2.003 1.630	2.004 1.602
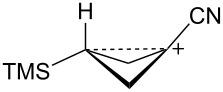 **74b**	Si–C3 C1–C3	2.046 1.694	2.045 1.688	2.037 1.652	2.028 1.663	2.031 1.623
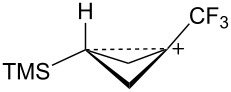 **54**	Si–C3 C1–C3	2.034 1.646	2.037 1.642	2.024 1.616	2.019 1.623	2.024 1.595

**Scheme 16:**
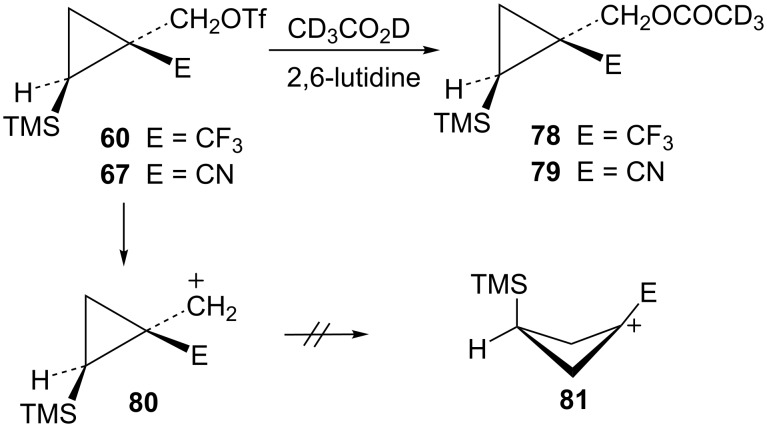
Reactions of triflates **60** and **67** in CD_3_CO_2_D.

The final item to be addressed is the behavior of triflates **60** and **67** with electron-withdrawing CF_3_ and CN groups *cis* to the trimethylsilyl group. These substrates gave exclusively unrearranged substitution products **78** and **79** when reacted in CD_3_CO_2_D (Scheme 16). The lack of rearrangement products suggests that these potent electron-withdrawing groups make further rearrangement of cations **80** untenable. Indeed, M062X/6-311+G** calculations show that the potential rearranged cation **81** (E = CN) is not even an energy minimum, but a transition state.

## Conclusion

1-Substituted-*cis*-2-trimethylsilylyclopropylcarbinyl mesylates and triflates **13** solvolyze in CD_3_CO_2_D to give products derived from 3-trimethylsilylcyclobutyl cations. These cationic intermediates are stabilized by a long-range rear lobe interaction with the γ-trimethylsilyl group. When the substituent is electron-withdrawing (CF_3_, CN, or CO_2_CH_3_), significant amounts of bicyclobutane products are formed. The bicyclobutanes are a result of γ-trimethylsilyl elimination from the cationic intermediate. Computational studies support a carbocation intermediate with an unusually long Si–C bond, indicative of increased demand for Si–C hyperconjugation due to the electron-withdrawing group. With the exception of the phenyl substitution, the chemistry of *trans*-derivatives **14** is quite different since these substrates are geometrically precluded from forming γ-trimethylsilyl-stabilized cyclobutyl cations.

## Experimental

Full experimental details are given in Supporting Information File 1.

## Supporting Information

Full experimental details, ^1^H and ^13^C NMR spectra of new compounds, and M062X/6-311+G** computational studies are presented as Supporting Information.

File 1 Experimental details and ^1^H and ^13^C NMR spectra of new compounds.

File 2 M062X/6-611+G** calculated structures, energies, and Cartesian coordinates for carbocations and transition states.
