# Supplementary material for: The cyclopropylcarbinyl route to γ-silyl carbocations
Source: Beilstein J Org Chem. 2019 Jul 24;15:1769–80. doi: 10.3762/bjoc.15.170 (PMC6664402; doi:10.3762/bjoc.15.170)

BEILSTEIN JOURNAL OF ORGANIC CHEMISTRY

Supporting Information

for

The cyclopropylcarbinyl route to γ-silyl carbocations

Xavier Creary

Beilstein J. Org. Chem. **2019,** *15,* 1769–1780. doi:10.3762/bjoc.15.170

Experimental details and ^{1}H and ^{13}C NMR spectra of new compounds

License and Terms: This is a supporting information file under the terms of the Creative Commons Attribution License (http://creativecommons.org/licenses/by/4.0). Please note that the reuse, redistribution and reproduction in particular requires that the authors and source are credited.
The license is subject to the *Beilstein Journal of Organic Chemistry* terms and conditions: (https://www.beilstein-journals.org/bjoc)

General. NMR spectra were recorded on a Varian DirectDrive 600 MHz spectrometer. HRMS measurements were carried out using either a Brucker MicroTOF-II spectrometer (electrospray ionization source with time-of-flight mass analyzer) or a Micromass GCT Premier spectrometer (gas chromatography mode with electron impact ionization mode).

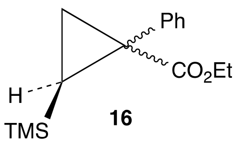

16

Photochemical reaction of ethyl 2-diazo-2-phenylacetate with vinyltrimethylsilane. A solution of 430 mg of $PhCN_2CO_2Et$ [1] in 10.2 mL of vinyltrimethylsilane was placed in a sealed Pyrex tube under N_2. The solution was irradiated with a Hanovia 450 Watt lamp for 3 h and 50 min as the tube was cooled in a stream of air. During this time the color substantially faded. The solution was then transferred to a flask and the excess vinyltrimethylsilane was removed by distillation at approximately 200 mm pressure. The last traces of vinyltrimethylsilane were removed under aspirator vacuum. The residue was distilled using a vacuum pump to give 298 mg (50% yield) of a mixture of esters **16**, bp 85-90 °C (0.2 mm). This mixture was used directly in the $LiAlH_4$ reduction step.

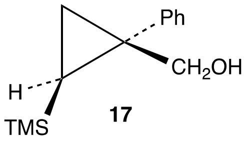

17

and

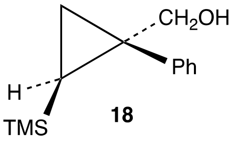

18

Reaction of esters 16 with $LiAlH_4$. Lithium aluminum hydride (105 mg; 2.767 mmol) was suspended in 5 mL of ether and a solution of 298 mg (1.137 mmol) of the ester mixture **16** in 10 mL of ether was added dropwise with stirring at room temperature. The reaction was exothermic. After 15 min at room temperature a solution a solution of 1 g of 10% NaOH was added dropwise with stirring. Stirring was continued until

the precipitated aluminum salts turned white. A small amount of $MgSO_4$ was added, the mixture was then filtered, and the ether solvent was removed using a rotary evaporator. The residue was chromatographed on 7.4 g of silica gel and the column was eluted with increasing amounts of ether in pentane. Nothing eluted with 2-6% ether in pentane. Alcohol **17** (86 mg) eluted first with 8% ether in pentane, followed by a small amount of a mixture of **17** and **18**. Alcohol **18** (56 mg) next eluted with 8-11% ether in pentane. ^1H NMR of **17** (600 MHz, $CDCl_3$) δ 7.37-7.34 (m, 2 H), 7.33-7.29 (m, 2 H), 7.24-7.20 (m, 1 H), 3.82 (d of d, *J* = 11.4, 1.1 Hz, 1 H), 3.64 (d, *J* = 11.4 Hz, 1 H), 1.43 (br, 1 H), 1.22 (d of d of d, *J* = 10.2, 3.9, 1.1 Hz, 1 H), 0.83 (d of d, *J* = 7.9, 3.9 Hz, 1 H), 0.14 (s, 9 H), 0.11 (d of d, *J* = 10.2, 7.9 Hz, 1 H). ^{13}C NMR of **17** (150 MHz, $CDCl_3$) δ 144.5, 129.0, 128.4, 126.6, 69.0, 33.7, 15.5, 12.9, -0.3. HRMS (ESI) (M + Na^+) calculated for $C_{13}H_{20}NaOSi$ 243.1176, found 243.1147.

^1H NMR of **18** (600 MHz, $CDCl_3$) δ 7.38-7.33 (m, 2 H), 7.32-7.27 (m, 2 H), 7.24-7.21 (m, 1 H), 3.93 (d of d, *J* = 11.2, 1.0 Hz, 1 H), 3.33 (d, *J* = 11.2 Hz, 1 H), 1.45 (br s, 1 H), 1.02-0.98 (m, 2 H), 0.06 (d of d, *J* = 9.6, 8.1 Hz, 1 H), -0.30 (s, 9 H). ^{13}C NMR of **18** (150 MHz, $CDCl_3$) δ 140.9, 130.5, 128.2, 126.9, 73.3, 33.7, 12.6, 10.4, -1.6. HRMS (ESI) (M + Na^+) calculated for $C_{13}H_{20}NaOSi$ 243.1176, found 243.1140.

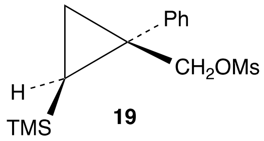

19

Preparation of mesylate 19. A solution of 39 mg (0.177 mmol) of alcohol **18** and 29 mg (0.253 mmol) of CH_3SO_2Cl in 1.5 mL of CH_2Cl_2 was cooled to −10 °C and 32 mg (0.317 mmol) of triethylamine in a small amount of CH_2Cl_2 was added dropwise. The mixture was warmed to room temperature and then transferred to a separatory funnel using 3 mL of

ether. Pentane (2 mL) pentane was added and the mixture was then rapidly washed successively with ice water, cold dilute HCl solution, ice water, and saturated NaCl solution. The organic extract was dried over a mixture of Na_2SO_4 and $MgSO_4$, filtered and the solvent was removed using a rotary evaporator. The yield of mesylate was 50 mg (94% yield). The neat mesylate is unstable for long periods at room temperature and was stored in ether/pentane solution at -20 °C. ^{1}H NMR of **19** (600 MHz, $CDCl_3$) δ 7.36-7.33 (m, 2 H), 7.32-7.29 (m, 2 H), 7.25-7.21 (m, 1 H), 4.36 (d of d, *J* = 10.5, 0.7 Hz, 1 H), 4.33 (d, *J* = 10.5 Hz, 1 H), 2.63 (s, 3 H), 1.34 (d of d of d, *J* = 10.3, 4.2, 0.8 Hz, 1 H), 0.96 (d of d, *J* = 8.3, 4.2 Hz, 1 H), 0.26 (d of d, *J* = 10.3, 8.3 Hz, 1 H), 0.17 (s, 9 H). ^{13}C NMR of **19** (150 MHz, $CDCl_3$) δ 143.4, 129.1, 128.4, 127.0, 76.9, 37.2, 30.4, 16.0, 13.6, -0.5.

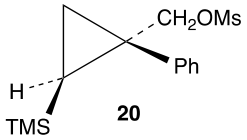

20

Preparation of mesylate 20. A solution of 39 mg (0.177 mmol) of alcohol **18** and 32 mg (0.279 mmol) of CH_3SO_2Cl in 1.5 mL of CH_2Cl_2 was cooled to −10 °C and 35 mg (0.347 mmol) of triethylamine in a small amount of CH_2Cl_2 was added dropwise. The mixture was warmed to room temperature and then transferred to a separatory funnel using about 3 mL of ether. About 2 mL of pentane was added and the mixture was then rapidly washed successively with ice water, cold dilute HCl solution, ice water, and saturated NaCl solution. The organic extract was dried over a mixture of Na_2SO_4 and $MgSO_4$, filtered and the solvent was removed using a rotary evaporator. The yield of mesylate was 48 mg (91% yield). The neat mesylate is unstable for long periods at room temperature and was stored in ether/pentane solution at -20 °C. NMR spectra were recorded at -5 °C since decomposition occurred slowly in $CDCl_3$ over the

time needed to record spectra at ambient temperature. ^{1}H NMR of **20** (600 MHz, $CDCl_3$, -5 °C) δ 7.38-7.35 (m, 2 H), 7.32-7.28 (m, 2 H), 7.26-7.22 (m, 1 H), 4.54 (d of d, *J* = 10.4, 1.2 Hz, 1 H), 3.98 (d, *J* = 10.4 Hz, 1 H), 2.60 (s, 3 H), 1.16-1.11 (m, 2 H), 0.20 (d of d, *J* = 10.5, 7.8 Hz, 1 H), -0.29 (s, 9 H). ^{13}C NMR of **20** (150 MHz, $CDCl_3$, -5 °C) δ 139.4, 130.5, 128.3, 127.4, 81.1, 37.1, 30.2, 13.4, 11.3, -1.8.

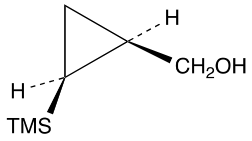

Preparation of *cis*-1-(hydroxymethyl)-2-trimethylsilylcyclopropane. A solution of 1.4 mL of 1.4 M EtZnI (1.960 mmol) was placed in flask under N_2 and 250 mg (0.933 mmol) of CH_2I_2 in 0.3 mL of ether was added in one portion. After 5 min, 60 mg (0.462 mmol) of (*Z*)-3-(trimethylsilyl)prop-2-en-1-ol [2] in 0.5 mL of ether was added dropwise. The reaction was exothermic. The mixture was warmed in a 35 °C bath for 1 h, quenched with dilute HCl solution, and the ether extract was the diluted with pentane. The organic extract was then washed with water and dilute NH_3 solution. After drying over $MgSO_4$, the solvent was removed using a rotary evaporator. The residue was chromatographed on 6.4 g of silica gel. The column was eluted with increasing amounts of ether in pentane. *cis*-1-(Hydroxymethyl)-2-trimethylsilylcyclopropane (39 mg) eluted with 12% ether in pentane. ^{1}H NMR of *cis*-1-(hydroxymethyl)-2-trimethylsilylcyclopropane (600 MHz, $CDCl_3$) δ 3.57 (d of d, *J* = 11.1, 6.9 Hz, 1 H), 3.45 (d of d, *J* = 11.1, 8.0 Hz, 1 H), 1.40 (br, 1 H), 1.36 (m, 1 H), 0.85 (d of d of d, *J* = 10.0, 7.0, 3.8 Hz, 1 H), 0.22 (m, 1 H), 0.04 (s, 9 H), -0.24 (t of d, *J* = 9.6, 7.7 Hz, 1 H). ^{13}C NMR of *cis*-1-(hydroxymethyl)-2-trimethylsilylcyclopropane (150 MHz, $CDCl_3$) δ 65.8, 19.4, 7.5, 2.3, -0.2.

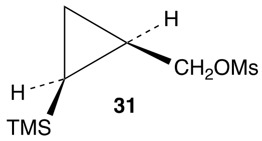

31

Preparation of mesylate 31. A solution of 20 mg (0.139 mmol) of *cis*-1-(hydroxymethyl)-2-trimethylsilylcyclopropane and 23 mg (0.201 mmol) of CH_3SO_2Cl in 1.5 mL of CH_2Cl_2 was cooled to −10 °C and 26 mg (0.257 mmol) of triethylamine in 0.3 mL of CH_2Cl_2 was added dropwise. The mixture was warmed to room temperature and then transferred to a separatory funnel using 3 mL of ether and 2 mL of pentane. The mixture was then rapidly washed successively with ice water, cold dilute HCl solution, ice water, and saturated NaCl solution. The organic extract was dried over a mixture of Na_2SO_4 and $MgSO_4$, filtered and the solvent was removed using a rotary evaporator to give 28 mg (91% yield) of mesylate 31 as an oil. Mesylate **31** was stored as an ether-pentane solution at -20 °C. ^{1}H NMR of **31** (600 MHz, $CDCl_3$) δ 4.20 (d of d, *J* = 10.6, 8.2 Hz, 1 H), 4.12 (d of d, *J* = 10.6, 7.6 Hz, 1 H), 3.03 (s, 3 H), 1.49 (m, 1 H), 0.98 (d of d of d, *J* = 10.0, 7.7, 4.2 Hz, 1 H), 0.37, (d of t, *J* = 8.0, 4.4 Hz, 1 H), 0.07 (s, 9 H), -0.09 (d of t. *J* = 11.3, 9.7 Hz, 1 H). ^{13}C NMR of **31** (150 MHz, $CDCl_3$) δ 73.5, 38.0, 15.6, 8.2. 3.4, -0.4

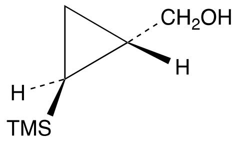

Preparation of *trans*-1-(hydroxymethyl)-2-trimethylsilylcyclopropane. Ten mL of 1.4 M EtZnI (14.00 mmol) was placed in a flask under N_2 and 1.669 g (6.23 mmol) of CH_2I_2 in 2 mL of ether was added dropwise. After 5 min, a solution of 405 mg (3.12 mmol) of (*E*)-3-(trimethylsilyl)prop-2-en-1-ol [3] in 2 mL of ether was added dropwise over 5 min. The reaction was exothermic. The mixture was then refluxed for 90 min, cooled in an ice bath, and quenched with dilute HCl solution. The mixture was then transferred to a separatory funnel

and 10 mL of pentane was added. The organic extract was washed with $Na_2S_2O_3$ solution and dilute NaOH solution. After drying over $MgSO_4$, the solvent was removed via rotary. The residue was chromatographed on 8 g of silica gel and the column was eluted with increasing amounts of ether in pentane. *trans*-1-(Methoxymethyl)-2-trimethylsilylcyclopropane (70 mg) eluted with 6% ether in pentane. *trans*-1-(Hydroxymethyl)-2-trimethylsilylcyclopropane containing a small amount of unreacted (*E*)-3-(trimethylsilyl)prop-2-en-1-ol eluted with 12% ether in pentane. *trans*-1-(Hydroxymethyl)-2-trimethylsilylcyclopropane [4] (257 mg) eluted with 14–15% ether in pentane. ^1H NMR of *trans*-1-(hydroxymethyl)-2-trimethylsilylcyclopropane (600 MHz, $CDCl_3$) δ 3.48 (d of d, *J* = 11.0, 6.5 Hz, 1 H), 3.44 (d of d, *J* = 11.0, 7.0 Hz, 1 H), 1,41 (br, 1 H), 1.01 (m, 1 H), 0.48-0.44 (m, 2 H), 0.04 (s, 9 H), 0.49 (m, 1 H). ^{13}C NMR of *trans*-1-(hydroxymethyl)-2-trimethylsilylcyclopropane (150 MHz, $CDCl_3$) δ 68.7, 18.0, 7.1, 2.2, -2.4.

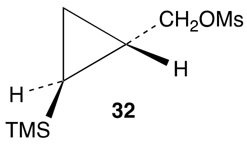

32

Preparation of mesylate 32. A solution of 47 mg (0.326 mmol) of *trans*-1-(hydroxymethyl)-2-trimethylsilylcyclopropane and 53 mg (0.463 mmol) of CH_3SO_2Cl in 2.0 mL of CH_2Cl_2 was cooled to −10 °C and 59 mg (0.584 mmol) of triethylamine in 0.5 mL of CH_2Cl_2 was added dropwise. The mixture was warmed to room temperature and then transferred to a separatory funnel using 3 mL of ether. Pentane (3 mL) was added and the mixture was then rapidly washed successively with ice water, cold dilute HCl solution, ice water, and saturated NaCl solution. The organic extract was dried over a mixture of Na_2SO_4 and $MgSO_4$, filtered and the solvent was removed using a rotary evaporator to give 65 mg (90 % yield) of mesylate **32** as

an unstable oil. The mesylate was immediately re-dissolved in ether/pentane and stored at −20 °C. NMR spectra were recorded at −5 °C. ^{1}H NMR of **32** (600 MHz, $CDCl_3$, -5 °C) δ 4.12 (d of d, J = 10.5, 7.0 Hz, 1 H), 4.08 (d of d, J = 10.5, 7.5 Hz, 1 H), 3.02 (s, 3 H), 1.12 (m, 1 H), 0.65-0.57 (m, 2 H), -0.03 (s, 9 H), -0.32 (m, 1 H). ^{13}C NMR of **32** (150 MHz, $CDCl_3$, -5 °C) δ 76.4, 37.8, 14.1, 8.1, 3.5, -2.5.

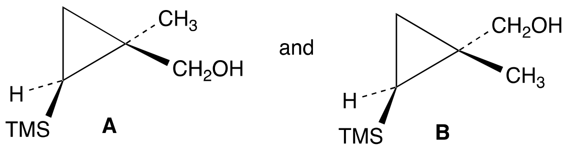

Preparation of 1-methyl-2-(trimethylsilyl)-cyclopropylmethanol. Benzophenone (1.00 g) was dissolved in 16 mL of vinyltrimethylsilane and 210 mg of methyl 2-diazopropanoate [1] was added. The solution was placed in a Pyrex tube and the air-cooled tube was irradiated with a Hanovia 450 W lamp for 75 min. During this time the yellow color substantially faded. The solution was then transferred to a round-bottom flask and the excess vinyltrimethylsilane was distilled off at atmospheric pressure. The residue was transferred to a 15 mL flask and a short path distillation head was attached. The pressure was reduced to 0.3 mm using a vacuum pump, the receiver flask was cooled in dry ice, and the residue was distilled. The more volatile products collected at less than 55 °C (slight warming with a heat gun). The benzophenone does not distill at this pressure and temperature. Methyl 1-methyl-2-(trimethylsilyl)cyclopropanecarboxylate (290 mg; 85% yield) was collected as a mixture of stereoisomers.

The ester mixture above (290 mg; 1.559 mmol) was dissolved in 3 mL of ether and this solution was added dropwise to 250 mg (6.588 mmol) of $LiAlH_4$ suspended in 5 mL of ether. On completion of the addition, a solution of 0.1 g on NaOH in 0.9 g of water was added carefully

dropwise to the mixture. After 30 min, $MgSO_4$ was added with stirring to the ether and the mixture was filtered. The solids were washed thoroughly with ether and the ether was removed using a rotary evaporator. The crude products were chromatographed on 9 g of silica gel. The column was eluted with increasing amounts of ether in pentane. The ((1*S**,2*S**)-1-methyl-2-(trimethylsilyl)cyclopropyl)methanol, **A**, eluted with 14% ether in pentane. This was followed by mixture of **A** and ((1*R**,2*S**)-1-methyl-2-(trimethylsilyl)cyclopropyl)methanol, **B**, and then by pure **B**. NMR spectra of **A** were recorded in DMSO-d_6 since **A** decomposed slowly in $CDCl_3$ that had a trace of HCl. ^1H NMR of **A** (600 MHz, DMSO-d_6) δ 4.48 (d of d, *J* = 5.9, 4.5 Hz, 1 H), 3.27 (d of d of d, *J* = 10.8, 5.9, 0.8 Hz, 1 H), 3.20 (d of d, *J* = 10.8, 4.5 Hz, 1 H), 1.10 (s, 3 H), 0.50 (d of d of d, *J* = 9.9, 3.3, 0.8 Hz, 1 H), 0.35 (d of d, *J* = 7.4, 3.3 Hz, 1 H), -0.01 (s, 9 H), -0.53 (d of d, *J* = 9.8, 7.4 Hz, 1 H). ^{13}C NMR of **A** (150 MHz, DMSO-d_6) δ 66.7, 23.9, 23.6, 15.8, 11.2, 0.0. HRMS (EI) (M^+) calculated for $C_8H_{18}OSi$ 158.1127, found 158.1135.

Alcohol **B** was identical to the product formed by cyclopropanation of (*E*)-2-methyl-3-(trimethylsilyl)prop-2-en-1-ol with CH_2I_2/EtZnI. ^1H NMR of **B** (600 MHz, $CDCl_3$) δ 3.35 (s, 2 H), 1.18 (s, 3 H), 0.65 (d of d, *J* = 10.4, 3.7 Hz, 1 H), 0.34 (d of d, *J* = 7.2, 3.7 Hz, 1 H), 0.04 (s, 9 H), -0.42 (d of d, *J* = 10.5, 7.2 Hz, 1 H). ^{13}C NMR of **B** (150 MHz, $CDCl_3$) δ 73.6, 23.4, 18.3, 15.3, 9.2, -0.3.

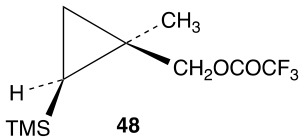

48

Preparation of trifluoroacetate 48. A solution of 29.6 mg of alcohol **A** prepared above (0.187 mmol) and 44 mg of 2,6-lutidine (0.411 mmol) in 3 mL of ether was cooled to −10 °C and 75 mg of trifluoroacetic anhydride (0.357 mmol) in a small amount of

ether was added dropwise. The mixture was warmed to 0 °C and then transferred to a separatory funnel using an additional 2 mL of ether. Pentane (3 mL) was added and the mixture was then rapidly washed successively with ice water, cold dilute HCl solution, ice water, and saturated NaCl solution. The organic extract was dried over a mixture of Na_2SO_4 and $MgSO_4$, filtered, and the solvent was removed using a rotary evaporator to give 41 mg (87% yield) of trifluoroacetate **48**. The trifluoroacetate **48** was stored in ether/pentane at −20 °C. ^{1}H NMR of **48** (600 MHz, $CDCl_3$) δ 4.37 (d, *J* = 10.8 Hz, 1 H), 4.05 (d, *J* = 10.8 Hz, 1 H), 1.21 (s, 3 H), 0.74 (d of d, *J* = 10.2, 4.2 Hz, 1 H), 0.54 (d of d, *J* = 7.8, 4.2 Hz, 1 H), 0.04 (s, 9 H), -0.27 (d of d, *J* = 9.6, 7.8 Hz, 1 H). ^{13}C NMR of **48** (150 MHz, $CDCl_3$) δ 157.7 (q, *J* = 42 Hz), 114.7 (q, *J* = 286 Hz), 74.9, 23.8, 20.5, 16.3, 12.8, -0.6.

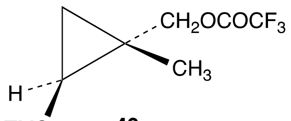

49

Preparation of trifluoroacetate 49. The synthesis of trifluoroacetate **49** (86% yield) from alcohol **B** prepared above was analogous to the preparation of **48**. ^{1}H NMR of **49** (600 MHz, $CDCl_3$) δ 4.17 (d, *J* = 10.8 Hz, 1 H), 4.09 (d, *J* = 10.8 Hz, 1 H), 1.18 (s, 3 H), 0.81 (d of d, *J* = 10.6, 4.1 Hz, 1 H), 0.46 (d of d, *J* = 7.6, 4.2 Hz, 1 H), 0.05 (s, 9 H), -0.26 (d of d, *J* = 10.7, 7.6 Hz, 1 H). ^{13}C NMR of **49** (150 MHz, $CDCl_3$) δ 157.7 (q, *J* = 42 Hz), 114.7 (q, *J* = 286 Hz), 78.6, 20.0, 18.45, 15.9, 10.4, -0.6.

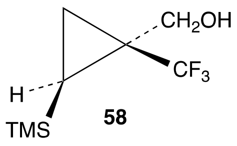

58

and

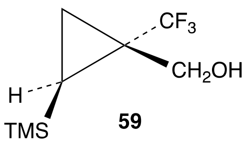

59

Preparation of 1-(trifluoromethyl)-2-(trimethylsilyl)cyclopropylmethanol, 58 and 59. Benzophenone (815 mg) was dissolved in 13.7

mL of vinyltrimethylsilane and 321 mg of the $CF_3CN_2CO_2Et$, **56** [5], was added. The solution was placed in a Pyrex tube and the air cooled tube was irradiated with a Hanovia 450 W lamp for 85 min. The solution was then transferred to a round-bottom flask and the excess vinyltrimethylsilane was distilled off at atmospheric pressure. The residue was transferred to a 15 mL flask and a short path distillation head was attached. The pressure was reduced to 0.2 mm using a vacuum pump to remove any remaining volatiles. The receiver flask was cooled in a dry ice bath and the more volatile products were collected at less than 50 °C. A trace of benzophenone contaminated the ester products **57** that distilled at this temperature and pressure. This product was re-distilled to remove the trace of benzophenone. The yield of esters **57** (mixture of stereoisomers) was 356 mg, bp 70–75 °C (15 mm).

Lithium aluminum hydride (200 mg; 5.270 mmol) was suspended in 5 mL of ether and a solution of 389 mg (1.531 mmol) of the ester mixture **57** in 3 mL of ether was added dropwise at room temperature. On completion of the addition, an additional 10 mL of ether was added to the mixture followed by careful addition of a solution of 0.1 g on NaOH in 1.0 g of water. After addition of $MgSO_4$ the mixture was the filtered using a Buchner funnel and the ether solvent was removed using a rotary evaporator. The crude products were chromatographed on 9 g of silica gel and the column was eluted with increasing amounts of ether in pentane. Nothing eluted with 2–6% ether in pentane. Alcohol **59** (96 mg) eluted with 8–10% ether in pentane. This was followed by a mixture of alcohols **59** and **58**. Alcohol **58** (94 mg) next eluted with 10-12% ether in pentane. ^{1}H NMR of **58** (600 MHz, $CDCl_3$) δ 3.85 (d of d, *J* = 12.4, 6.4 Hz, 1 H), 3.54 (d of d, *J* = 12.4, 6.6 Hz, 1 H), 1.72 (t, *J* = 6.5 Hz, 1 H), 0.99-0.90 (m, 2 H), 0.11-0.06 (m, 10 H). ^{13}C NMR of **58** (150 MHz, $CDCl_3$) δ 127.9 (q, *J* = 275 Hz), 66.3 (q, *J* = 1.8 Hz), 30.1 (q, *J* =

31.3 Hz), 11.9 (q, J = 2.2 Hz), 9.7 (q, J = 1.2 Hz), -1.0 (q, J = 2.1 Hz). HRMS (EI) $(M - CH_3)^+$ calculated for $C_7H_{12}F_3OSi$ 197.0610, found 197.0609.

^1H NMR of **59** (600 MHz, $CDCl_3$) δ 3.86 (d of d, J = 12.9, 6.0 Hz, 1 H), 3.65 (d of d, J = 12.9, 6.8 Hz, 1 H), 1.59 (t of q, J = 6.4, 0.9 Hz, 1 H), 1.23 (d of d, J = 11.0, 4.5 Hz, 1 H), 0.67 (m, 1 H), 0.30 (d of d, J = 11.0, 8.6 Hz, 1 H), 0.10 (s, 9 H). ^{13}C NMR of **59** (150 MHz, $CDCl_3$) δ 127.5 (q, J = 275 Hz), 62.6 (q, J = 1.2 Hz), 29.0 (q, J = 30.4 Hz), 11.6 (q, J = 2.5 Hz), 7.8, -0.9. HRMS (EI) $(M - CH_3)^+$ calculated for $C_7H_{12}F_3OSi$ 197.0610, found 197.0619.

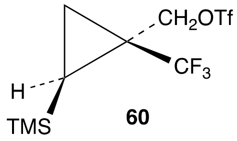

60

Preparation of triflate 60. A solution of 35 mg (0.165 mmol) of alcohol **58** and 34 mg (0.317 mmol) of 2,6-lutidine in 2.5 mL of CH_2Cl_2 was cooled to −10 °C and 84 mg (0.298 mmol) of triflic anhydride in 0.3 mL of CH_2Cl_2 was added dropwise. The mixture was then warmed to room temperature and then transferred to a separatory funnel using 6 mL of pentane. The mixture was then rapidly washed successively with ice water, cold dilute HCl solution, ice water, and saturated NaCl solution. The organic extract was dried over a mixture of Na_2SO_4 and $MgSO_4$. The solution was filtered and the solvent was removed using a rotary evaporator to give 52 mg (92% yield) of triflate **60** as an oil. The triflate was stored in pentane at -20 °C. ^1H NMR of **60** (600 MHz, $CDCl_3$) δ 4.71 (d, J = 11.5 Hz, 1 H), 4.35 (d, J = 11.5 Hz, 1 H), 1.21-1.15 (m, 2 H), 0.26 (t of m, J = 10.3 Hz, 1 H), 0.11 (q, J = 0.9 Hz, 9 H). ^{13}C NMR of **60** (150 MHz, $CDCl_3$) δ 126.2 (q, J = 275 Hz), 118.6 (q, J = 319 Hz), 79.9, 27.8 (q, J = 34.4 Hz), 13.6 (q, J = 1.8 Hz), 12.2, -1.2 (q, J = 2.0 Hz).

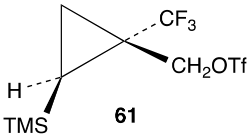

61

Preparation of Triflate 61. This procedure from alcohol **59** was analogous to the formation of triflate **60**. The yield of triflate **61** was 92%. This triflate was stored in pentane at -20 °C. ^{1}H NMR of **61** (600 MHz, $CDCl_3$) δ 4.71 (d, *J* = 11.6 Hz, 1 H), 4.50 (d, *J* = 11.6 Hz, 1 H), 1.47 (d of d, *J* = 11.2, 5.0 Hz, 1 H), 0.86 (m, 1 H), 0.53 (d of d, *J* = 11.1, 9.1 Hz, 1 H), 0.14 (s, 9 H). ^{13}C NMR of **61** (150 MHz, $CDCl_3$) δ 125.7 (q, *J* = 275 Hz), 118.6 (q, *J* = 319 Hz), 76.2, 27.5 (q, *J* = 33.5 Hz), 12.8 (q, *J* = 1.9 Hz), 9.6, -1.2.

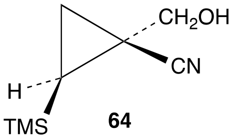

64

and

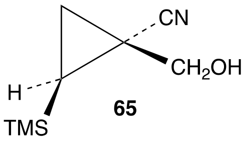

65

Preparation of 1-(cyano)-2-(trimethylsilyl)-cyclopropylmethanol, 64 and 65. A solution of 145 mg of $NCCN_2CO_2CH_3$, **62** [6], in 12 mL of vinyltrimethylsilane was placed in a Pyrex tube and the air cooled tube was irradiated with a Hanovia 450 W lamp for 90 min. The excess vinyltrimethylsilane was removed by distillation at atmospheric pressure. The residue was chromatographed on 7 g of silica gel and the column was eluted with increasing amounts of ether in pentane. The cyanoesters **63** (120 mg; 53% yield) eluted as a mixture of stereoisomers with 4–5% ether in pentane.

A solution of 170 mg (0.863 mmol) of esters **63** in 11 mL of ether was stirred under nitrogen as 32 mg (1.469 mmol) of $LiBH_4$ was added. After 5 h and 45 min at room temperature 10 mL of additional ether was added and the mixture was carefully quenched by adding 15 mL of 2% HCl in water. The organic phase was washed with water and then saturated NaCl solution. After drying over a mixture of Na_2SO_4 and $MgSO_4$ the solvent was removed using a rotary evaporator. The residue was chromatographed on 7.7 g of silica gel. The column was

eluted with increasing amounts of ether in pentane. Nothing eluted with 5–35% ether in pentane. Cyanoalcohol **65** eluted first with 38% ether in pentane. This was followed by mixture of **65** and **64**. Pure alcohol **64** also eluted with 38% ether in pentane. ^{1}H NMR of **64** (600 MHz, $CDCl_3$) δ 3.63 (d of d, *J* = 11.5, 5.6 Hz, 1 H), 3.60 (d of d, *J* = 11.5, 5.6 Hz, 1 H), 1.97 (t, *J* = 6.1 Hz, 1 H), 1.18-1.12 (m, 2 H), 0.18 (d of d, *J* = 10.4, 8.8 Hz, 1 H), 0.16 (s, 9 H). ^{13}C NMR of **64** (150 MHz, $CDCl_3$) δ 122.0, 68.0, 16.7, 15.9, 11.7, -2.1. HRMS (ESI) (M + H^+) calculated for $C_8H_{16}NOSi$ 170.0996, found 170.0961.

^{1}H NMR of **65** (600 MHz, $CDCl_3$) δ 3.66 (d of d, *J* = 11.8, 6.5 Hz, 1 H), 3.61 (d of d, *J* = 11.8, 5.8 Hz, 1 H), 2.08 (t, *J* = 6.2 Hz, 1 H), 1.54 (d of d of d, *J* = 11.0, 4.4, 0.7 Hz, 1 H), 0.87 (d of d, *J* = 9.1, 4.5 Hz, 1 H), 0.56 (d of d, *J* = 11.0, 9.1 Hz, 1 H), 0.10 (s, 9 H). ^{13}C NMR of **65** (150 MHz, $CDCl_3$) δ 123.5, 64.4, 17.5, 16.2, 13.7, -0.8. HRMS (ESI) (M + Na^+) calculated for $C_8H_{15}NOSi$ 192.0815, found 192.0805.

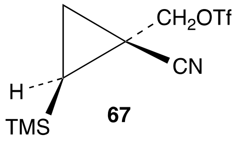

67

Preparation of triflate 67. This procedure from alcohol **64** was analogous to the formation of triflate **60**. The yield of triflate **67** was 85%. ^{1}H NMR of **67** (600 MHz, $CDCl_3$) δ 4.53 (d, *J* = 10.9 Hz, 1 H), 4.35 (d, *J* = 10.9 Hz, 1 H), 1.43-1.37 (m, 2 H), 0.41 (d of d, *J* = 10.7, 9.4 Hz, 1 H), 0.19 (s, 1 H). ^{13}C NMR of **67** (150 MHz, $CDCl_3$) δ 119.3, 118.5 (q, *J* = 320 Hz), 80.8, 18.0, 14.4, 13.9, -2.3.

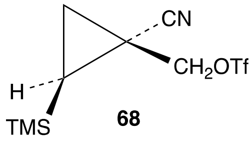

68

Preparation of triflate 68. This procedure from alcohol **65** was analogous to the formation of triflate **60**. The yield of triflate **68** was 91%. This triflate was stored in pentane at -20 °C. ^{1}H NMR of **68** (600 MHz, $CDCl_3$) δ 4.49 (d, *J* = 11.0 Hz, 1 H), 4.44 (d, *J* = 11.0 Hz, 1 H), 1.78 (d of d of d, *J* = 11.0, 4.9, 0.6 Hz, 1 H), 1.09 (d of d, *J* = 9.6, 4.9 Hz, 1 H), 0.82 (d of d, *J* = 11.0, 9.6 Hz, 1 H), 0.15 (s, 9 H). ^{13}C NMR of **68** (150 MHz, $CDCl_3$) δ 120.7, 118.6 (q, *J* = 320 Hz), 77.3, 17.7, 16.1, 14.5, -1.1.

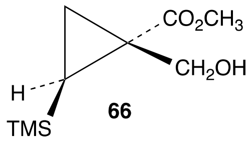

66

Preparation of alcohol 66. A solution of 16.5 mg (0.098 mmol) of alcohol **65** in 4 mL of ether containing 50 mg (1.563 mmol) of CH_3OH was cooled in ice-salt bath and HCl gas was slowly bubbled into the solution for five min. The mixture was kept at room temperature for 20 h and then the solvents were removed using a rotary evaporator. The residue was dissolved in 2 mL of water and the mixture was kept at room temperature for 60 h. The solution was then saturated with Na_2SO_4 and extracted with ether. The ether extract was dried over $MgSO_4$, filtered, and the solvent was removed using a rotary evaporator to give 14.9 mg (76% yield) of the ester **66** as an oil. ^{1}H NMR of **66** (600 MHz, $CDCl_3$) δ 3.72 (d of d, *J* = 12.2, 7.0 Hz, 1 H), 3.70 (s, 3 H), 3.64 (d of d, *J* = 12.2, 6.9 Hz, 1 H), 2.59 (t, *J* = 6.9 Hz, 1 H), 1.50 (d of d of d, *J* = 10.9, 3.4, 0.8 Hz, 1 H), 0.82 (d of d, *J* = 8.9, 3.4 Hz, 1 H), 0.62 (d of d, *J* = 10.8, 8.9 Hz, 1 H), 0.09 (s, 9 H). ^{13}C NMR of **66** (150 MHz, $CDCl_3$) δ 176.1, 64.2, 52.1, 30.5, 18.0, 15.4, -0.7.

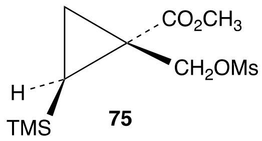
75

Preparation of mesylate 75. A solution of 8.4 mg (0.042 mmol) of alcohol **66** and 18 mg (0.157 mmol) of CH_3SO_2Cl in 1.0 mL of CH_2Cl_2 was cooled to −10 °C and 22 mg (0.218 mmol) of triethylamine in 0.2 mL of CH_2Cl_2 was added dropwise. The mixture was warmed to about 10 °C and then transferred to a separatory funnel using 2 mL of ether and 2 mL of pentane. The mixture was then rapidly washed successively with ice water, cold dilute HCl solution, ice water, and saturated NaCl solution. The organic extract was dried over a mixture of Na_2SO_4 and $MgSO_4$. The solution was filtered and the solvent was removed using a rotary evaporator to give 10.6 mg (91% yield) of mesylate **75** as an oil. ^1H NMR of **75** (600 MHz, $CDCl_3$) δ 4.40 (d, J = 10.6 Hz, 1 H), 4.33 (d, J = 10.6 Hz, 1 H), 3.70 (s, 3 H), 3.08 (s, 3 H), 1.63 (d of d of d, J = 10.9, 3.7, 0.4 Hz, 1 H), 0.95 (d of d, J = 9.2, 3.7 Hz, 1 H), 0.78 (d of d, J = 11.0, 9.2 Hz, 1 H), 0.11 (s, 9 H). ^{13}C NMR of **75** (150 MHz, $CDCl_3$) δ 173.6, 71.6, 52.4, 37.3, 27.5, 18.5, 16.3, -0.9.

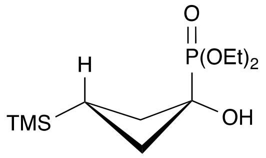

Preparation of diethyl 1-hydroxy-3-(trimethylsilyl)cyclobutyl phosphonate. A mixture of 402 mg (2.831 mmol) of 3-trimethylsilylcyclobutanone and 595 mg (2.833 mmol) of diethyl trimethylsilyl phosphite was sealed in a Pyrex tube and the tube heated at 100 °C for 43 h. The tube was opened and the residue was distilled to give 906 mg (91% yield) of diethyl 3-(trimethylsilyl)-1-(trimethylsilyloxy)-cyclobutylphosphonate, bp 83–84 °C (0.1 mm).

The silylated phosphonate above (884 mg) was dissolved in 9 mL of 10^{-3} M CF_3CO_2H in methanol. The mixture was stirred at room temperature for 24 h and the methanol was the removed using a rotary evaporator. The residue was distilled to give 679 mg (97% yield) of diethyl 1-hydroxy-3-(trimethylsilyl)cyclobutylphosphonate, bp 112-113 °C (0.1 mm). ^{1}H NMR (600 MHz, $CDCl_3$) δ 4.19 (quintet, *J* = 7.2 Hz, 4 H), 3.32 (br, 1 H), 2.55 (m, 2 H), 2.00 (m, 2 H), 1.51 (quintet, *J* = 10.2 Hz, 1 H), 1.34 (t, *J* = 7.1 Hz, 6 H), -0.04 (s, 9 H). ^{13}C NMR (150 MHz, $CDCl_3$) δ 71.1 (d, *J* = 156 Hz), 62.8 (d, *J* = 7.2 Hz), 33.7 (d, *J* = 1.2 Hz), 16.5 (d, *J* = 5.5 Hz), 12.5 (d, *J* = 3.4 Hz), -3.6. NMR spectra show the presence of 7% of the isomer with *trans* TMS and OH groups. HRMS (ESI) (M + H$^+$) calculated for $C_{11}H_{26}F_3O_4PSi$ 281.1332, found 281.1304.

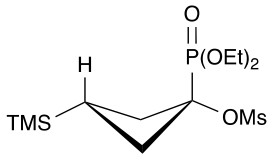

76

Preparation of mesylate 76. The diethyl 1-hydroxy-3-(trimethylsilyl) cyclobutyl phosphonate prepared above (284 mg; 1.014 mmol) was dissolved in 3 mL of CH_2Cl_2 and 260 mg (2.640 mmol) of CH_3SOCl [7] was added. The solution was cooled to −10 °C and a solution of 316 mg (3.129 mmol) of Et_3N in one mL of CH_2Cl_2 was added dropwise. The mixture was warmed to room temperature for 5 min and then transferred to a separatory funnel using ether and 5 mL of pentane. The mixture was washed with cold water, dilute HCl solution, cold water, saturated NaCl solution, and then dried over a mixture of Na_2SO_4 and $MgSO_4$. After filtration the solvents were removed using a rotary evaporator to give 321 mg (93% yield) of the sulfinate ester, which was immediately oxidized.

The sulfinate ester above (60 mg; 0.175 mmol) was dissolved in 5 mL of ether and 52 mg of 85% *m*-chloroperbenzoic acid (0.256 mmol) was added in a single portion. The mixture was stirred at room temperature for 20 h. Pentane (5 mL) was then added to the ether solution and the mixture was cooled in an ice bath. The mixture was then stirred vigorously as a solution of NaOH, NaI, and $Na_2S_2O_3$ in water was added. The aqueous phase was decanted and the organic extract was dried over $MgSO_4$. After filtration, solvent removal using a rotary evaporator left 55 mg (88% yield) of mesylate **76** as an oil. About 5% of the isomeric mesylate was present. ^{1}H NMR of **76** (600 MHz, $CDCl_3$) δ 4.26 (m, 4 H), 3.09 (s, 3 H), 2.80-2.65 (m, 4 H), 1.72 (quintet of m, *J* = 10.2 Hz, 1 H), 1.38 (t, *J* = 7.2 Hz, 6 H), 0.00 (s, 9 H). ^{13}C NMR of **76** (150 MHz, $CDCl_3$) δ 82.6 (d, *J* = 164 Hz), 63.6 (d, *J* = 7.0 Hz), 40.4, 33.6, 16.5 (d, *J* = 5.7 Hz), 14.5 (d, *J* = 1.5 Hz), -3.6.

Solvolyses of mesylates, triflates, and trifluoroacetates in CD_3CO_2D. Kinetics procedures. Rate data reported in Table 1 were all determined using ^{1}H NMR spectroscopy. Approximately 4 mg of the appropriate substrate was dissolved in 400 mg of CD_3CO_2D containing approximately 1.5 equivalents of 2,6-lutidine. A portion of this mixture was placed in a 3 mm NMR tube and the tube was immediately placed in a temperature controlled NMR probe at 20.0 °C. At appropriate time intervals, the tube was analyzed by ^{1}H NMR to determine amounts of remaining starting material. For most runs, the signal at δ 2.75 or δ 7.60 due to the 2,6-lutidine was used as an internal standard. For mesylates **19** and **20**, the upfield area in the TMS region was monitored. For mesylates **31** and **68**, the amount of covalent mesylate versus developing mesylate anion at δ 2.86 was monitored. For mesylate **69** and trifluoroacetates **48** and **49**, sealed tubes were placed in a constant temperature bath at elevated temperatures. The tubes were withdrawn from the bath at appropriate times and analyzed by NMR at room

temperature. First order rate constants for disappearance of substrates in Table 1 were determined by standard least squares methods. Typical evolving spectra and data are shown after this section.

Solvolyses of mesylates, triflates, and trifluoroacetates in CD_3CO_2D. Product analyses. A solution of about 5 mg of the appropriate substrate and 1.5 equivalents of 2,6-lutidine in about 400 mg of CD_3CO_2D was placed in a 3 mm NMR tube and the tube was kept at the appropriate temperature for 10 half-lives. The tube was then analyzed by ^{1}H NMR spectroscopy. Acetate products **21**, **34**, **38**-**41**, **50**, **51**, **70**, **71**, **78**, and **79** were identified by ^{1}H NMR spectral comparison with authentic samples of H_3 analogs in CD_3CO_2D. Bicyclobutanes **55** [8], **72b** [9], and **72c** [10] were identified by ^{1}H NMR spectral comparison with authentic samples. Authentic samples of acetates **70a**-H_3, **70b**-H_3, **70c**-H_3, **78**-H_3, and **79**-H_3 were prepared as described below.

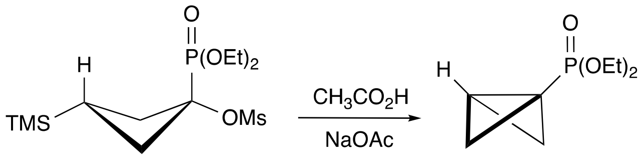

76 **77**

Solvolysis of mesylate 76 in CH_3CO_2H. A solution of 26 mg (0.317 mmol) of sodium acetate in 3.9 g of acetic acid was added to 55 mg (0.154 mmol) of mesylate **76**. The mixture was swirled and after 80 min at room temperature, 10 mL of ether and 5 mL of pentane were added. The mixture was cooled in an ice bath and 4 mL of water was added. The mixture was stirred as 2.6 g of NaOH was added in small portions. The mixture was periodically removed from the ice bath and after the NaOH had completely

dissolved, 2 mL of additional water was added followed by 100 mg of Na_2CO_3. The organic extract was dried over $MgSO_4$, filtered, and the solvents were removed using a rotary evaporator. The residue was chromatographed on 0.6 g of silica gel in a pipette. The bicyclobutane **77** (25 mg; 86% yield) eluted with 75% ether-15% pentane. ^1H NMR of **77** (600 MHz, $CDCl_3$) δ 4.12 (quintet, J = 7.3 Hz, 4 H), 2.13 (d of m, J = 22.8 Hz, 1 H), 1.95 (t, J = 3.3 Hz, 2 H), 1.33 (t, J = 7.2 Hz, 6 H), 0.93 (t, J = 2.3 Hz, 2 H). ^{13}C NMR of **77** (150 MHz, $CDCl_3$) δ 62.0 (d, J = 6.0 Hz), 34.5 (d, J = 5.7 Hz), 16.4 (d, J = 6.4 Hz), 7.9 (d, J = 8.0 Hz), -2.6 (d, J = 233 Hz). HRMS (ESI) (M + H^+) calculated for $C_8H_{15}F_3O_3P$ 191.0832, found 191.0828.

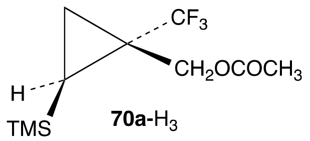

70a-H_3

Preparation of acetate 70a-H_3. A solution of 23.6 mg (0.111 mmol) of alcohol **59** and 22.5 mg (0.220 mmol) of acetic anhydride in 0.5 mL of CH_2Cl_2 was stirred as 7.8 mg (0.064 mmol) of dimethylaminopyridine was added in a single portion. After 2 h at room temperature the mixture was transferred to a separatory funnel using 7 mL of pentane. The mixture was then washed successively with water, dilute HCl solution, water, and K_2CO_3 solution. After drying over a mixture of Na_2SO_4 and $MgSO_4$, the solution was filtered and the solvent was removed using a rotary evaporator to give 24.3 mg (85% yield) of acetate **70a**-H_3 as an oil. ^1H NMR of **70a**-H_3 (600 MHz, $CDCl_3$) δ 4.46 (d, J = 12.8 Hz, 1 H), 3.93 (d, J = 12.8 Hz, 1 H), 2.08 (s, 3 H), 1.28 (d of d, J = 11.1, 4.6 Hz, 1 H), 0.70 (m, 1 H), 0.35 (d of d, J = 11.1, 8.8 Hz, 1 H), 0.09 (s, 9 H). ^{13}C NMR of **70a**-H_3 (150 MHz, $CDCl_3$) δ 170.7, 126.5 (q, J = 275 Hz), 63.7 (q, J = 0.9 Hz), 26.9 (q, J = 32.5 Hz), 20.9, 12.0 (q, J = 2.4 Hz), 8.0, -1.0. HRMS (ESI) (M + H^+) calculated for $C_{10}H_{18}F_3O_2Si$ 255.1023, found 255.1001.

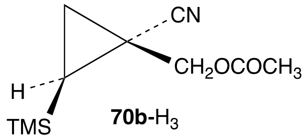

70b-H_3

Preparation of acetate 70b-H₃. The synthesis of acetate **70b**-H_3 from alcohol **65** was analogous to the preparation of **70a**-H_3. The yield of **70b**-H_3 was 88%. ^{1}H NMR of **70b**-H_3 (600 MHz, $CDCl_3$) δ 4.17 (d, *J* = 12.1 Hz, 1 H), 3.93 (d, *J* = 12.1 Hz, 1 H), 2.13 (s, 3 H), 1.60 (d of d of d, *J* = 11.1, 4.6, 0.7 Hz, 1 H), 0.94 (d of d, *J* = 9.3, 4.6 Hz, 1 H), 0.62 (d of d, *J* = 11.1, 9.3 Hz, 1 H), 0.10 (s, 9 H). ^{13}C NMR of **70b**-H_3 (150 MHz, $CDCl_3$) δ 170.5, 122.7, 65.3, 20.7, 16.8, 14.3, 14.0, -1.0.

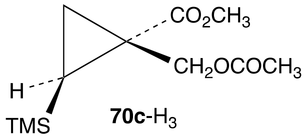

70c-H_3

Preparation of acetate 70c-H₃. The synthesis of acetate **70c**-H_3 from alcohol **66** was analogous to the preparation of **70a**-H_3. The yield of **70c**-H_3 was 94%. ^{1}H NMR of **70c**-H_3 (600 MHz, $CDCl_3$) δ 4.38 (d, *J* = 11.9 Hz, 1 H), 4.10 (d, *J* = 11.9 Hz, 1 H), 3.69 (s, 1 H), 2.07 (s, 3 H), 1.54 (d of d, *J* = 10.8, 3.4 Hz, 1 H), 0.84 (d of d, *J* = 9.0, 3.4 Hz, 1 H), 0.70 (d of d, *J* = 10.9, 9.0 Hz, 1 H), 0.08 (s, 9 H). ^{13}C NMR of **70c**-H_3 (150 MHz, $CDCl_3$) δ 174.5, 171.0, 65.5, 52.3, 27.4, 21.0, 18.2, 15.6, -0.8.

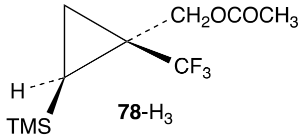

78-H_3

Preparation of acetate 78-H₃. The synthesis of acetate **77**-H_3 from alcohol **58** was analogous to the preparation of **70a**-H_3. The yield of **78**-H_3 was 90%. ^{1}H NMR of **78**-H_3 (600 MHz, $CDCl_3$) δ 4.27 (d, *J* = 12.3 Hz, 1 H), 4.03 (d, *J* = 12.3 Hz, 1 H), 2.08 (s, 3 H), 1.05-

0.98 (m, 2 H), 0.11 (m, 1 H), 0.07 (q, J = 0.8 Hz, 9 H). ^{13}C NMR of **78**-H_3 (150 MHz, $CDCl_3$) δ 170.9, 127.1 (q, J = 175 Hz), 67.1 (q, J = 1.7 Hz), 27.4 (q, J = 33 Hz), 20.9, 12.5 (q, J = 2.2 Hz), 10.8, -1.1 (q, J = 2.0 Hz).

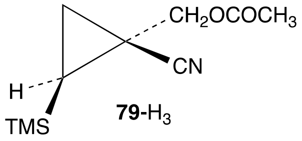

79-H_3

Preparation of acetate 79-H_3. The synthesis of acetate **79**-H_3 from alcohol **64** was analogous to the preparation of **70a**-H_3. The yield of **79**-H_3 was 94%. ^{1}H NMR of **79**-H_3 (600 MHz, $CDCl_3$) δ 4.07 (d, J = 11.7 Hz, 1 H), 4.01 (d, J = 11.7 Hz, 1 H), 2.12 (s, 3 H), 1.23 (d of d, J = 10.6, 4.7 Hz, 1 H), 1.19 (d of d, J = 9.0, 4.6 Hz, 1 H), 0.25 (d of d, J = 10.6, 9.0 Hz, 1 H), 0.15 (s, 9 H). ^{13}C NMR of **79**-H_3 (150 MHz, $CDCl_3$) δ 170.6, 121.2, 68.6, 20.8, 16.8, 13.6, 13.0, -2.2.

References for experimental section

1. Creary, X. *Org. Synth.* **1986**, *64*, 207-216.

2. (a) Paquette, L. A.; Ladouceur, G. *J. Org. Chem.* **1989**, *54*, 4278-4279. (b) Ilardi, E. A.; Stivala, C. E.; Zakarian, A. *Org. Lett*. **2008**, *10*, 1727-1730.

3. Jones, T. K.; Denmark, S. E. *Org. Synth*. **1990**, *7*, 524-527.

4. Kobayashi, S.; Imai, N.; Takahashi, H. Asymmetric preparation of metal-substituted cyclopropylmethanol derivatives. Jpn. Kokai Tokkyo Koho JP 07233175. Sep 5, 1995.

5. Shi, G.; Xu, Y. *J. Chem. Soc., Chem. Commun.* **1989**, 607-608.

6. This diazooester was prepared using the same procedure that was used to prepare the corresponding ethyl ester. See, Goddard-Borger, E. D.; Stick, R. V. *Org. Lett.* **2007**, *9*, 3797-3800.

7. Douglass, I. B.; Norton, R. V. *Org. Synth.* **1973***, 5*, 709-715.

8. Mercadante, M. A.; Kelly, C. B.; Hamlin, T. A.; Chiaie, K. R. D; Drago, M. D.; Duffy, K. K.; Dumas, M. T.; Fager, D. C.; Glod, B. L. C.; Hansen, K. E.; Hill, C. R.; Leising, R. M.; Lynes, C. L.; MacInnis, A. E.; McGohey, M. R.; Murray, S. A.; Piquette, M. C.; Roy, S. L.; Smith, R. M.; Sullivan, K. R.; Truong, B. H.; Vailonis, K. M.; Gorbatyuk, V.; Leadbeater, N. E.; Tilley, L. J. Chem. Sci. 2014, 5, 3983-3994.

9. (a) Pomerantz, M; Hillenbrad, D. F. Tetrahedron 1975, 31, 217-220. (b) Azran, C.; Hoz, S. *Tetrahedron* **1995**, *51*, 11421-11430.

10. Hall Jr., H. K.; Smith, C. D.; Blanchard Jr., E. P.; Cherkofsky, S. C.; Sieja, J. B. *J. Am. Chem. Soc.* **1971**, *93*, 121-130.

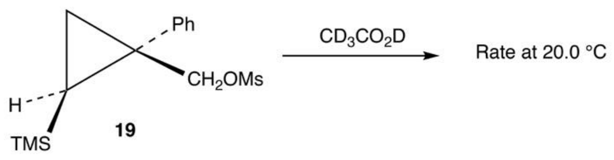

Time (min)	Area of Mesylate **19**
0	75.70
4	64.82
8	55.53
12	47.31
16	40.45
20	34.66
24	29.66
28	25.34
32	21.69
36	18.57
40	15.95

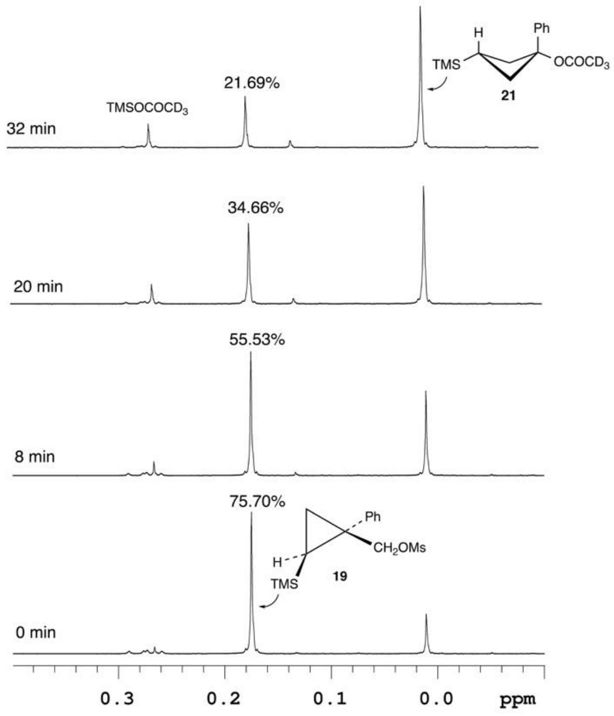

Evolving partial ^{1}H NMR spectra during reaction of **19** in CD_3CO_2D

First order plot of ln [area of **19**] vs. Time

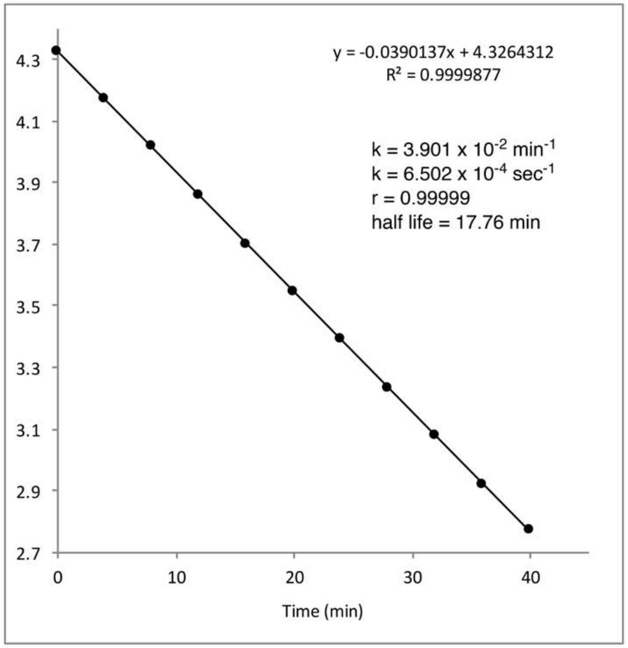

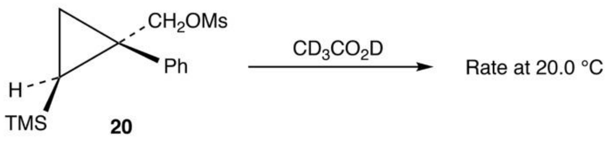

Time (min)	Area of Mesylate **20**
0	60.22
3	48.15
6	38.44
9	30.58
12	24.30
15	19.42
18	15.43

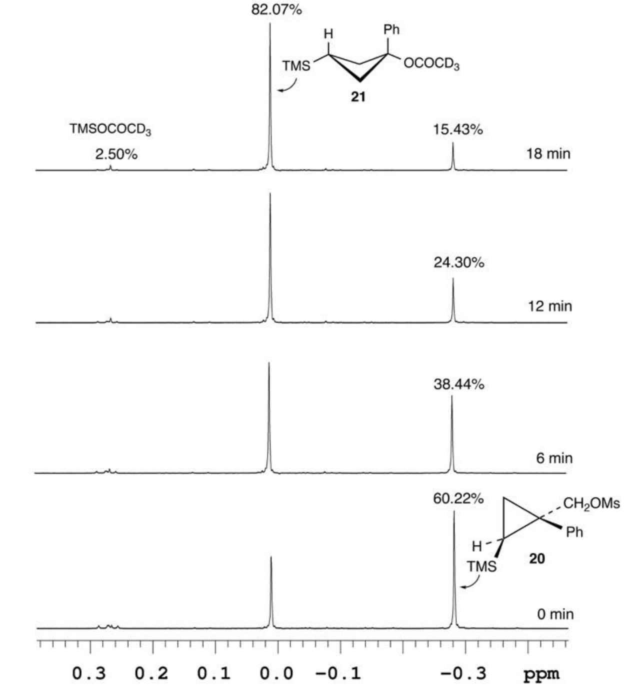

Evolving partial ^{1}H NMR spectra during reaction of **20** in CD_3CO_2D

First order plot of ln [% **20**] vs. Time

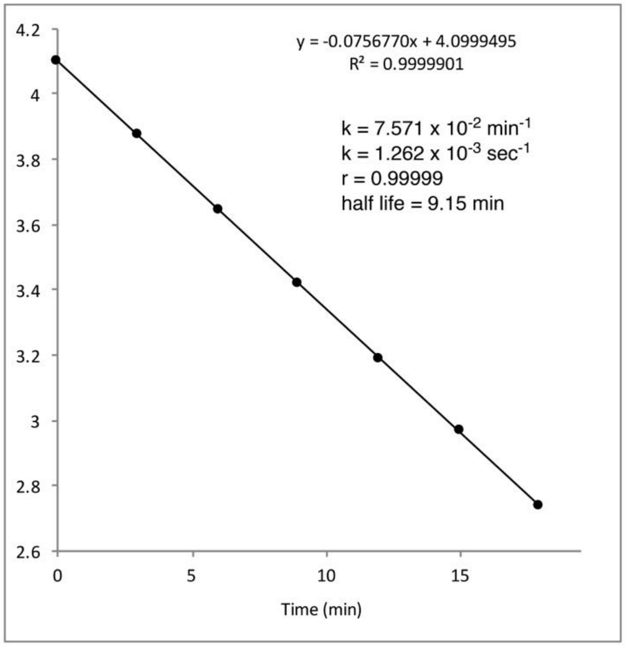

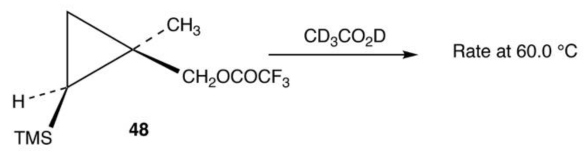

Time (hr)	Area of Trifluoroacetate **48**
0	89.88
2	74.64
4	62.10
6	51.93
8.3	41.73
10.4	34.32
12.5	28.01

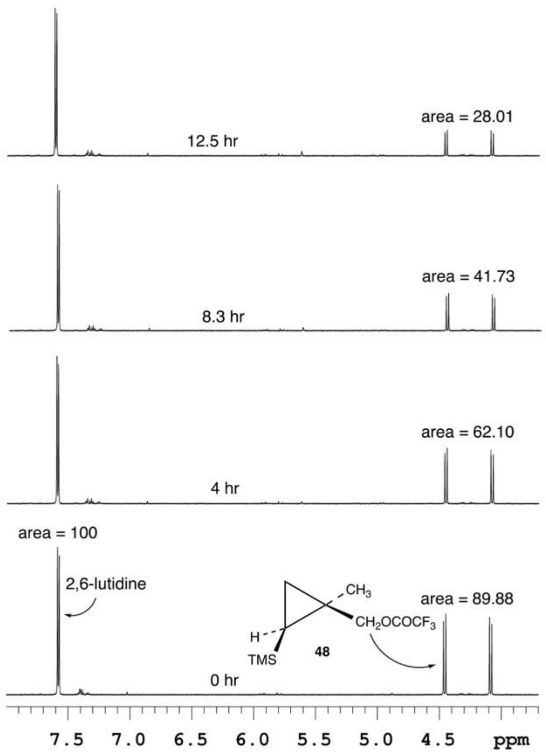

Evolving partial ^1H NMR spectra during reaction of **48** in CD_3CO_2D

First order plot of ln [area of **48**] vs. Time

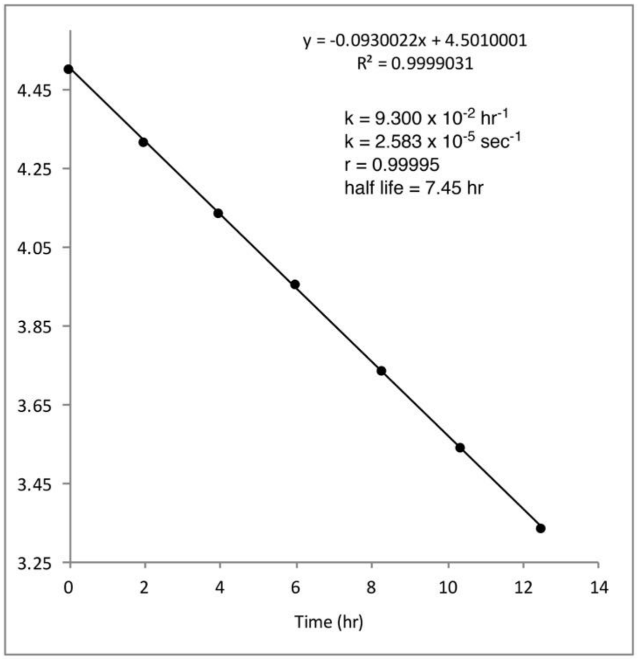

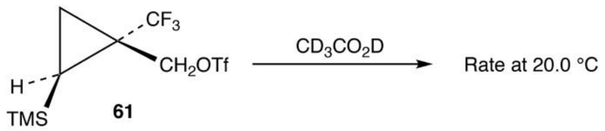

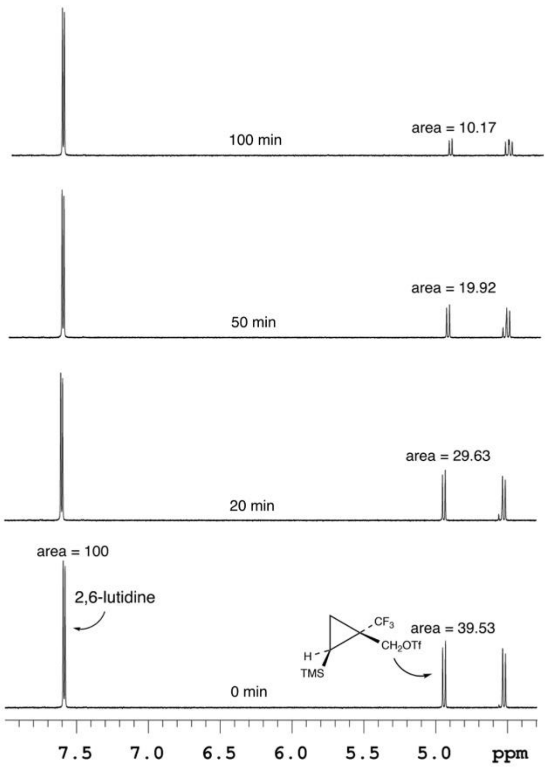

Evolving partial ^{1}H NMR spectra during reaction of **61** in CD_3CO_2D

Time (min)	Area of Triflate **61**
0	39.53
6	36.38
12	33.32
20	29.63
30	26.24
40	23.07
50	19.92
60	17.41
71	15.14
86	12.41
100	10.17

First order plot of ln [area of **61**] vs. Time

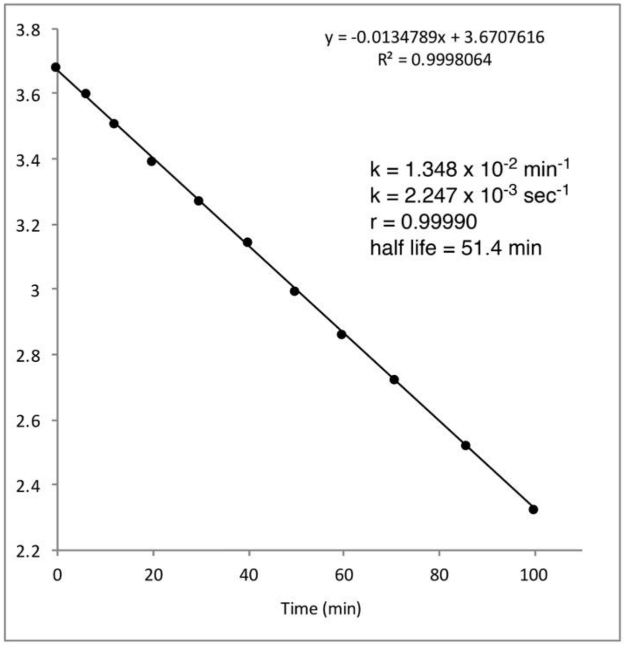

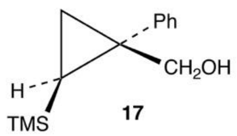

17

^{1}H NMR of **17** (600 MHz, $CDCl_3$)

7.37-7.34 (m, 2 H)
7.33-7.29 (m, 2 H)
7.24-7.20 (m, 1 H)
3.82 (d of d, J = 11.4, 1.1 Hz, 1 H)
3.64 (d, J = 11.4 Hz, 1 H)
1.43 (br, 1 H)
1.22 (d of d of d, J = 10.2, 3.9, 1.1 Hz, 1 H)
0.83 (d of d, J = 7.9, 3.9 Hz, 1 H)
0.14 (s, 9 H)
0.11 (d of d, J = 10.2, 7.9 Hz, 1 H)

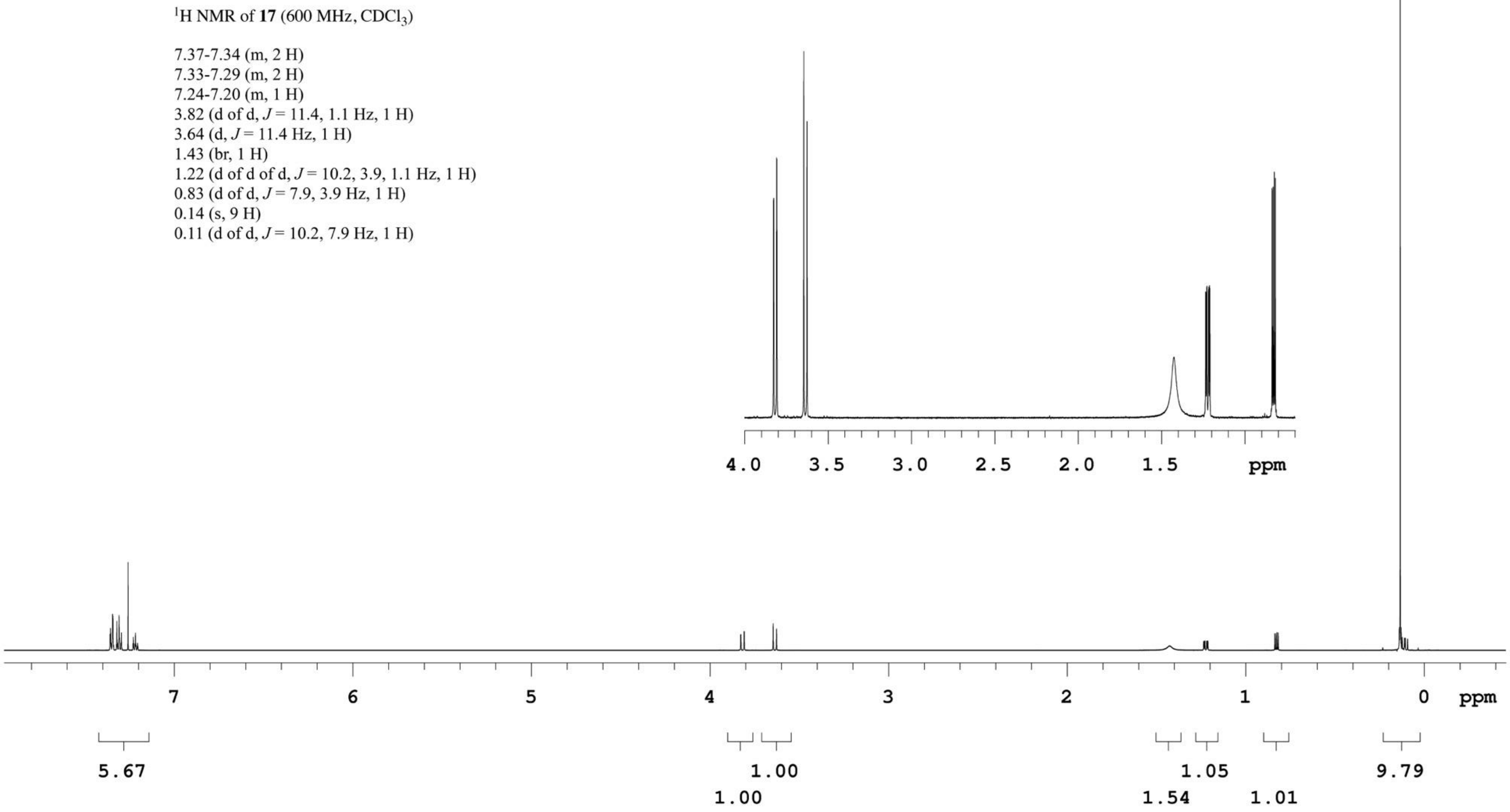

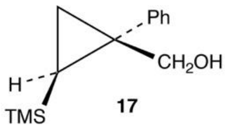

17

^{13}C NMR of **17** (150 MHz, $CDCl_3$)

144.5
129.0
128.4
126.6
69.0
33.7
15.5
12.9
-0.3.

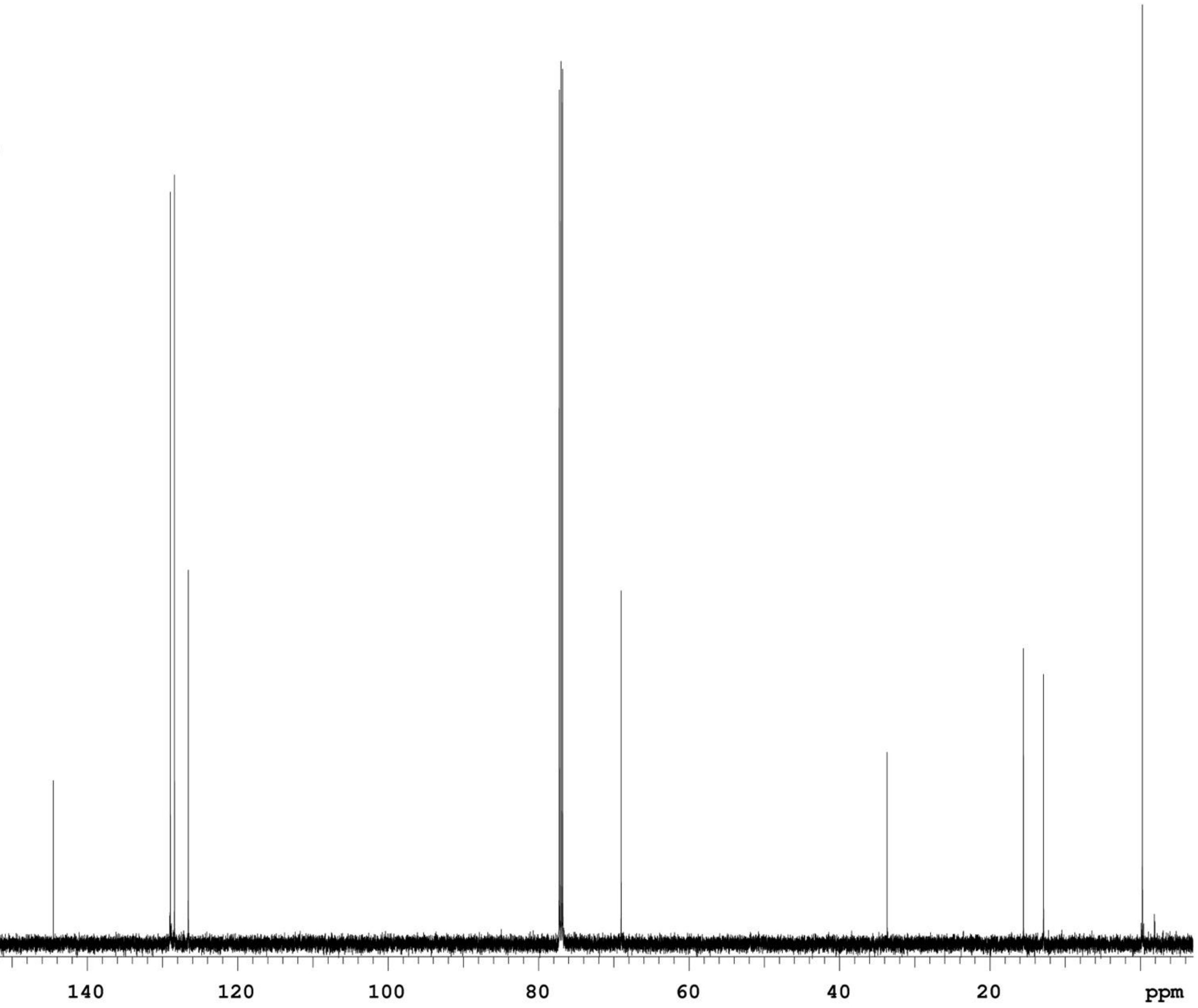

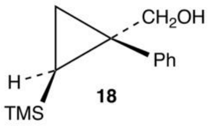

18

^{1}H NMR of **18** (600 MHz, $CDCl_3$)

7.38-7.33 (m, 2 H)
7.32-7.27 (m, 2 H)
7.24-7.21 (m, 1 H)
3.93 (d of d, J = 11.2, 1.0 Hz, 1 H)
3.33 (d, J = 11.2 Hz, 1 H)
1.45 (br s, 1 H)
1.02-0.98 (m, 2 H)
0.06 (d of d, J = 9.6, 8.1 Hz, 1 H)
-0.30 (s, 9 H)

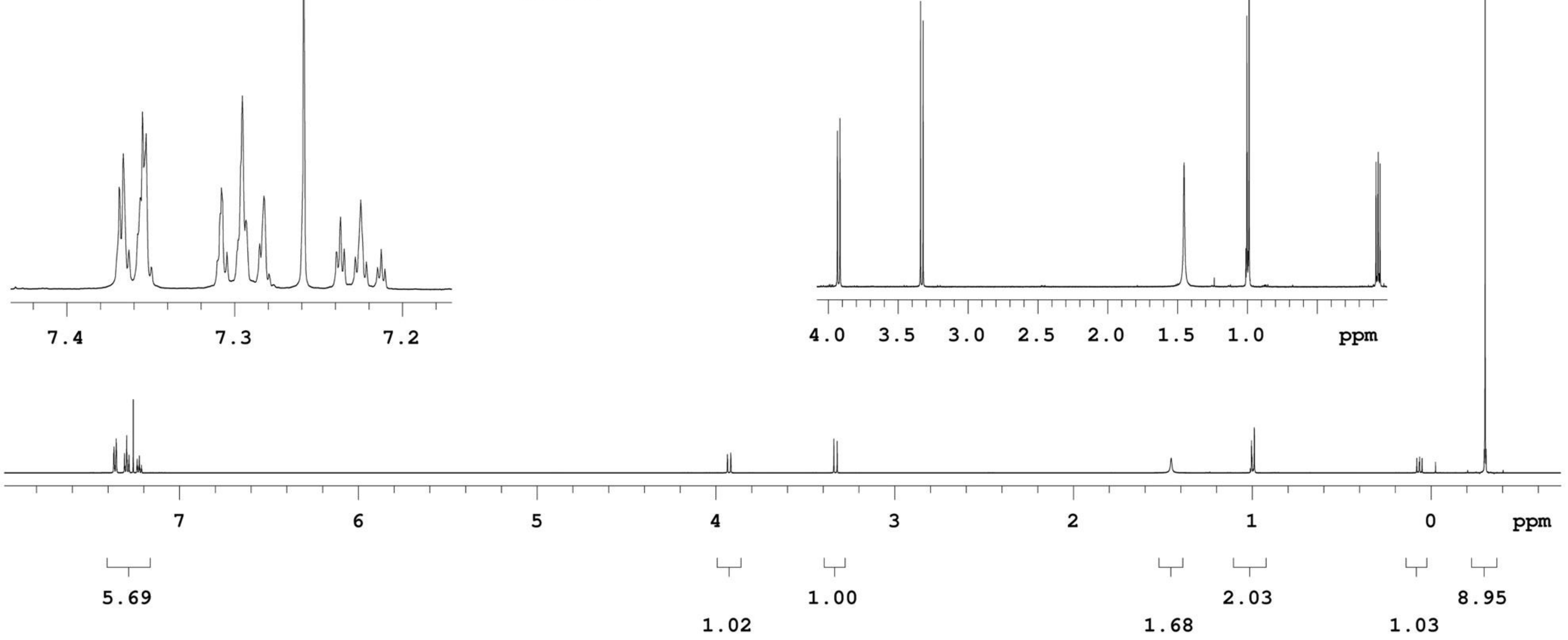

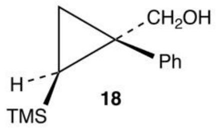

18

^{13}C NMR of **18** (150 MHz, $CDCl_3$)

140.9
130.5
128.2
126.9
73.3
33.7
12.6
10.4
-1.6.

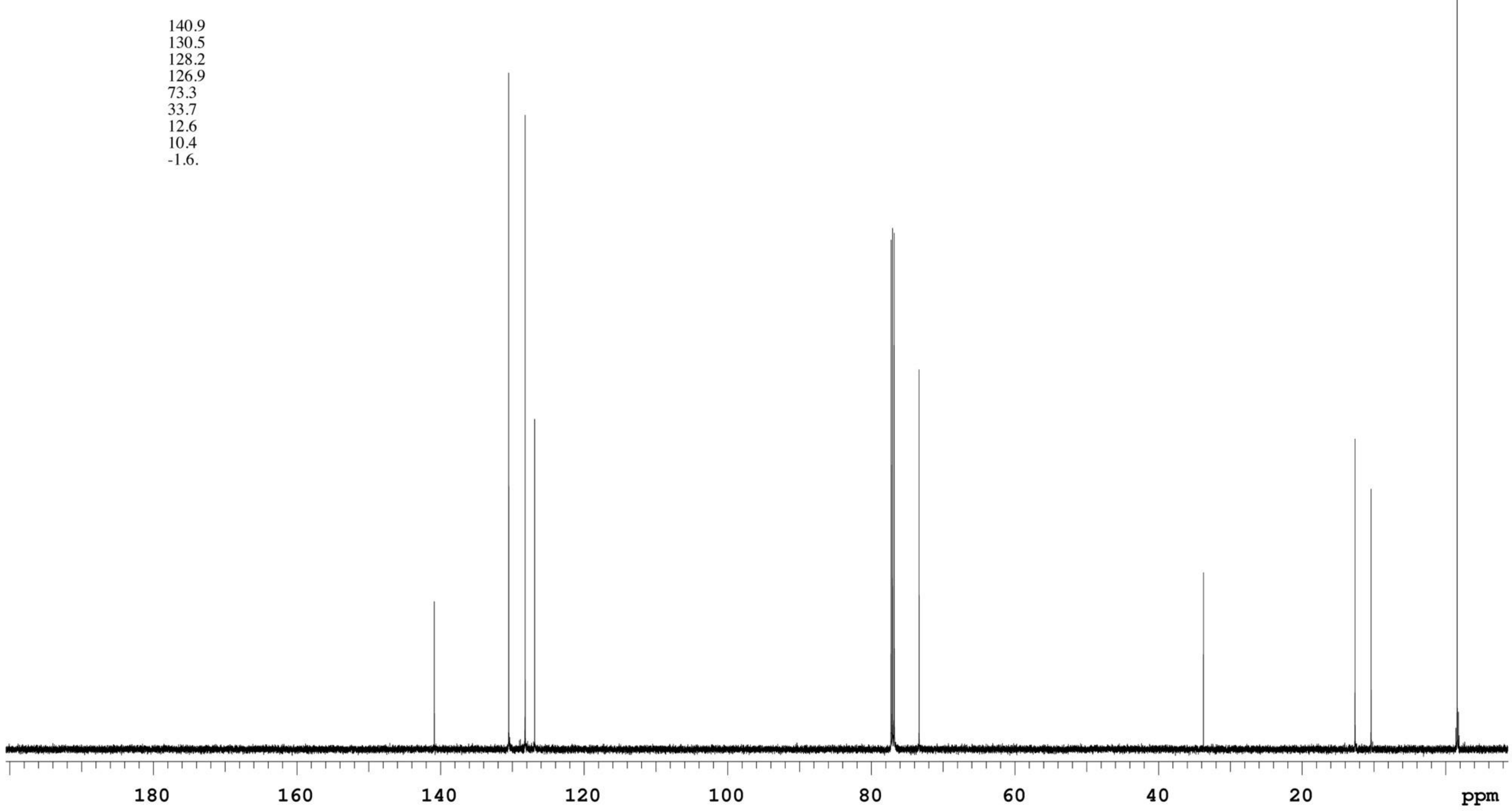

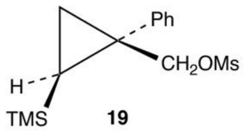

19

^{1}H NMR of **19** (600 MHz, $CDCl_3$)

7.36-7.33 (m, 2 H)
7.32-7.29 (m, 2 H)
7.25-7.21 (m, 1 H)
4.36 (d of d, J = 10.5, 0.7 Hz, 1 H)
4.33 (d, J = 10.5 Hz, 1 H)
2.63 (s, 3 H)
1.34 (d of d of d, J = 10.3, 4.2, 0.8 Hz, 1 H)
0.96 (d of d, J = 8.3, 4.2 Hz, 1 H)
0.26 (d of d, J = 10.3, 8.3 Hz, 1 H)
0.17 (s, 9 H)

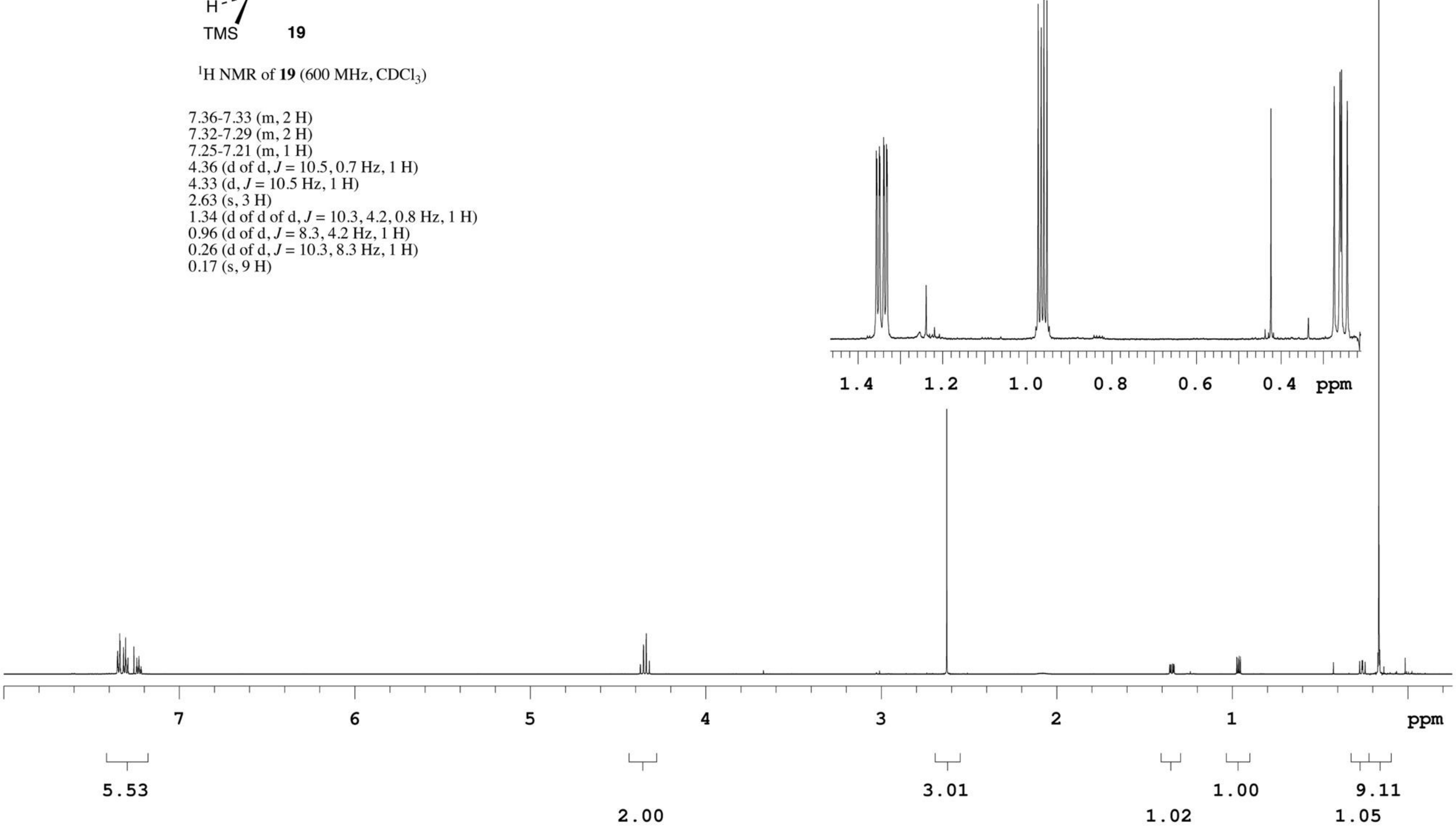

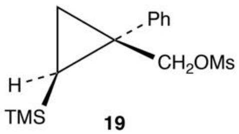

19

^{13}C NMR of **19** (150 MHz, $CDCl_3$)

143.4
129.1
128.4
127.0
76.9
37.2
30.4
16.0
13.6
-0.5

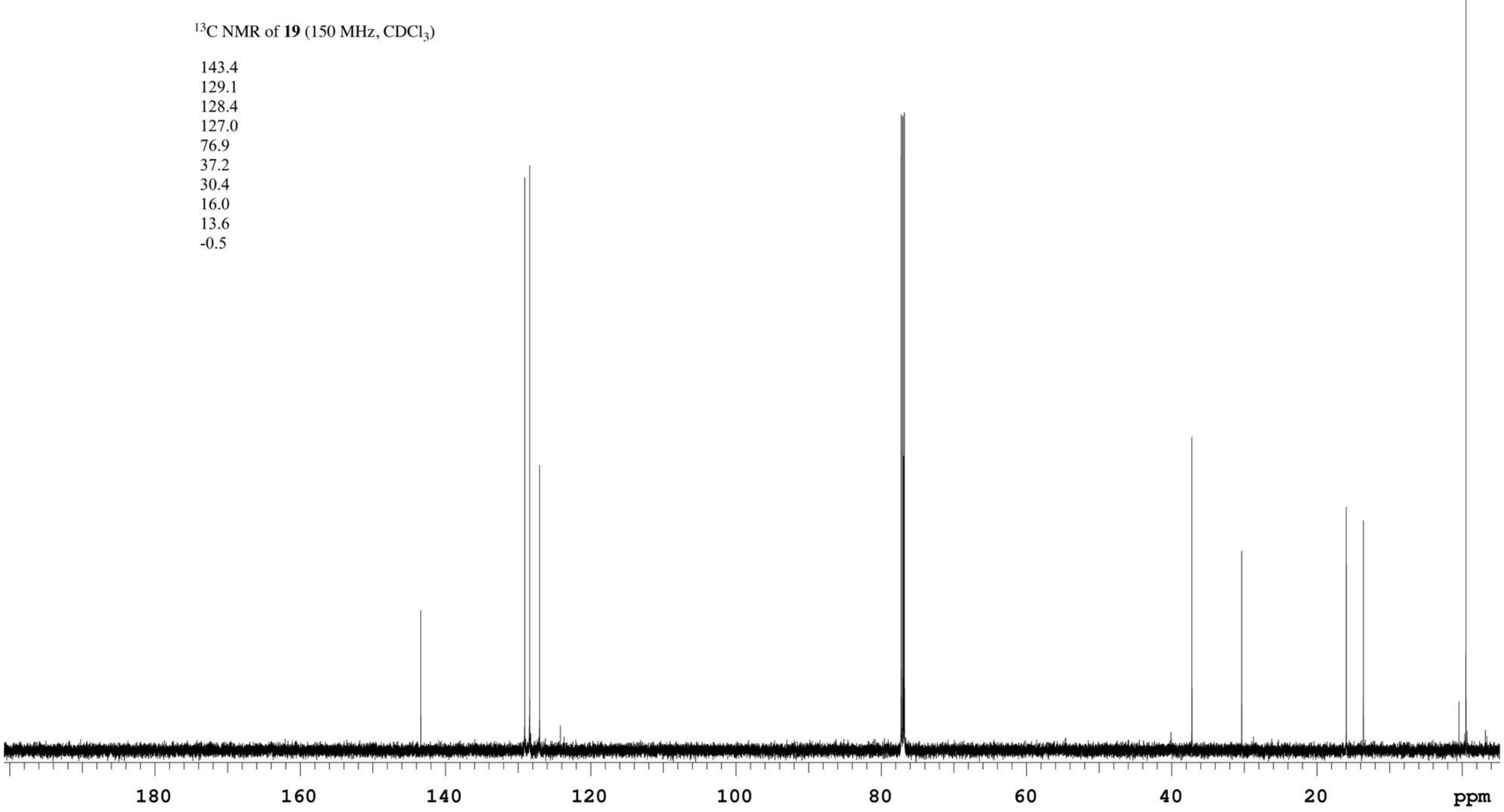

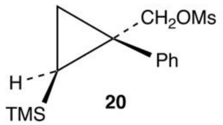

20

^{1}H NMR of **20** (600 MHz, $CDCl_3$)

7.38-7.35 (m, 2 H)
7.32-7.28 (m, 2 H)
7.26-7.22 (m, 1 H)
4.54 (d of d, J = 10.4, 1.2 Hz, 1 H)
3.98 (d, J = 10.4 Hz, 1 H)
2.60 (s, 3 H)
1.16-1.11 (m, 2 H)
0.20 (d of d, J = 10.5, 7.8 Hz, 1 H)
-0.29 (s, 9 H)

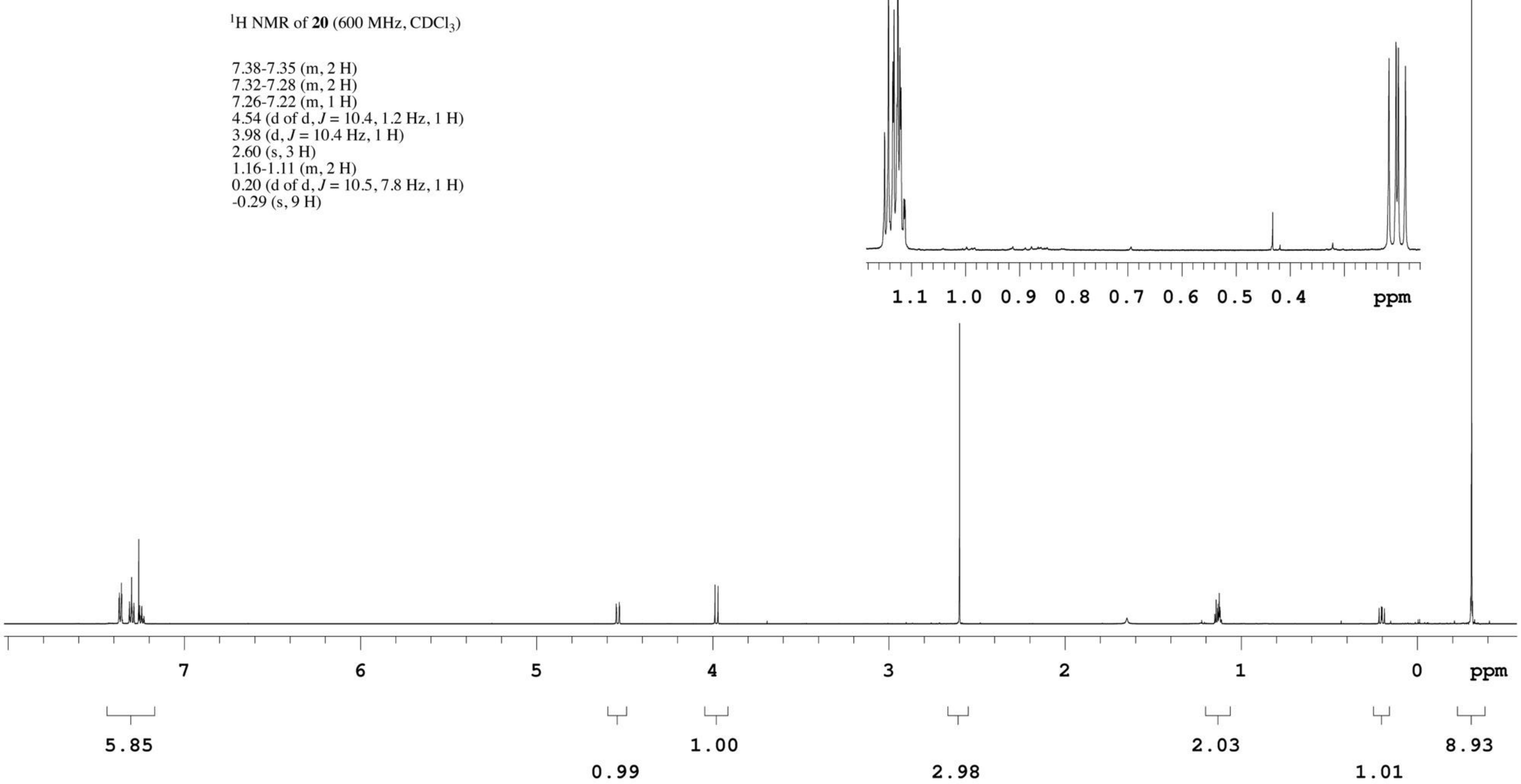

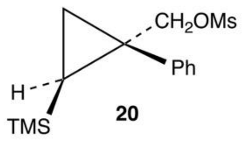

20

^{13}C NMR of **20** (150 MHz, $CDCl_3$)

139.4
130.5
128.3
127.4
81.1
37.1
30.2
13.4
11.3
-1.8

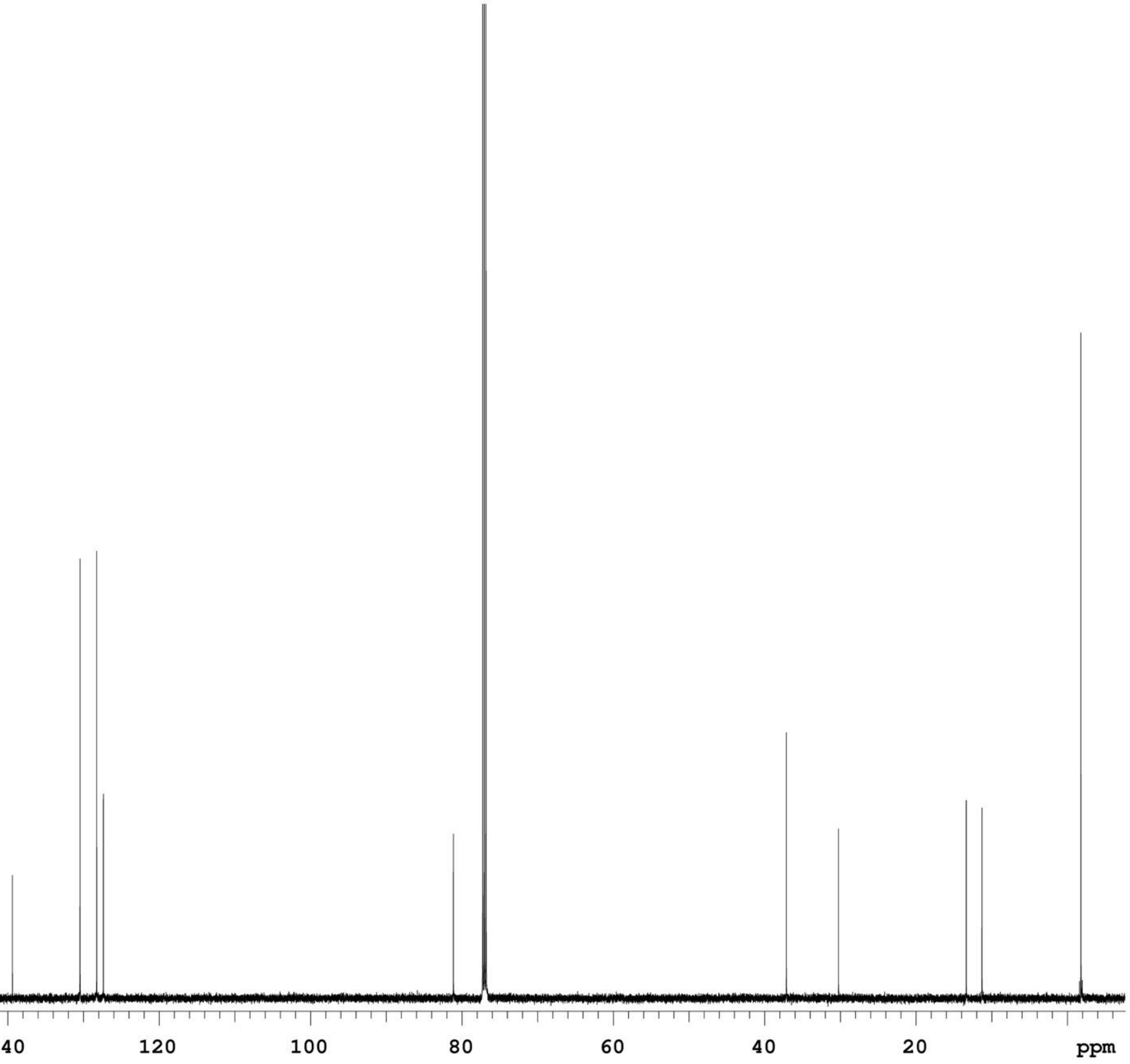

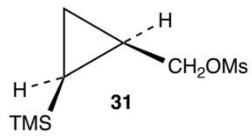

31

^{1}H NMR of **31** (600 MHz, $CDCl_3$)

4.20 (d of d, J = 10.6, 8.2 Hz, 1 H)
4.12 (d of d, J = 10.6, 7.6 Hz, 1 H)
3.03 (s, 3 H)
1.49 (m, 1 H)
0.98 (d of d of d, J = 10.0, 7.7, 4.2 Hz, 1 H)
0.37, (d of t, J = 8.0, 4.4 Hz, 1 H)
0.07 (s, 9 H)
-0.09 (d of t. J = 11.3, 9.7 Hz, 1 H)

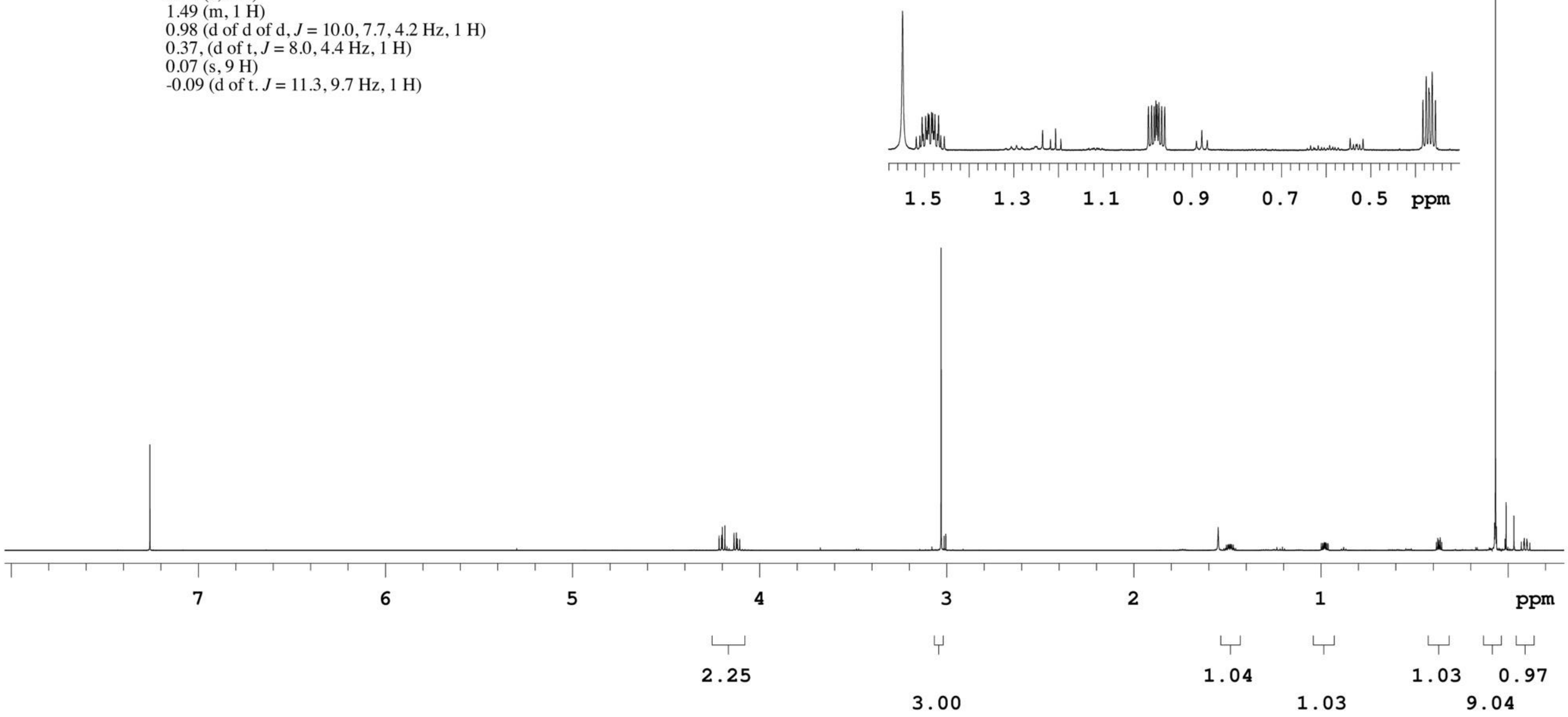

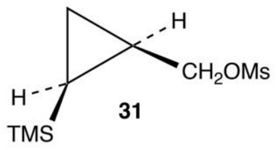

31

^{13}C NMR of **32** (150 MHz, $CDCl_3$)

73.5
38.0
15.6
8.2
3.4
-0.4.

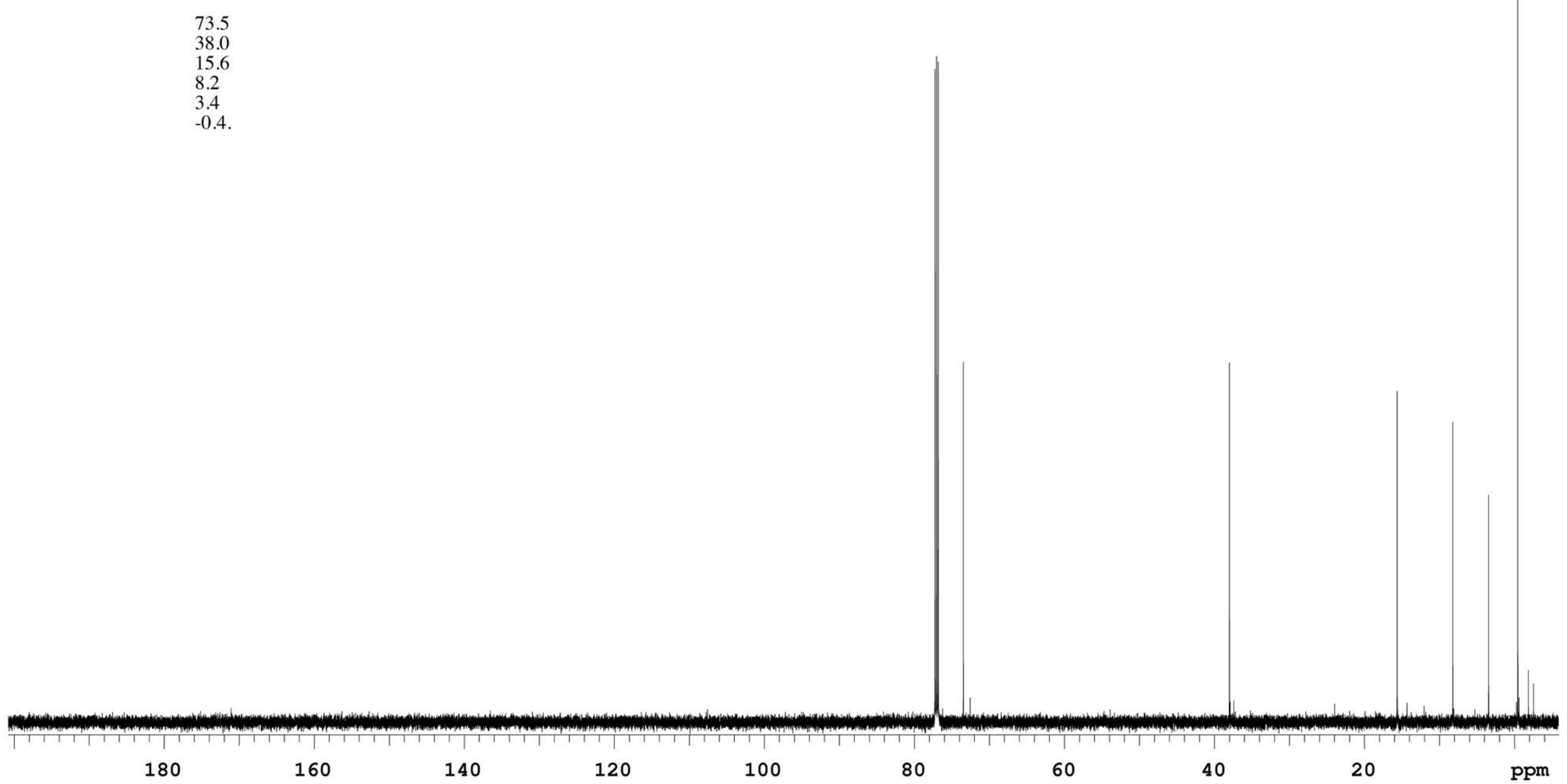

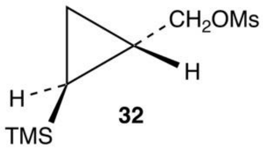

32

^{1}H NMR of **32** (600 MHz, $CDCl_3$)

4.12 (d of d, J = 10.5, 7.0 Hz, 1 H)
4.08 (d of d, J = 10.5, 7.5 Hz, 1 H)
3.02 (s, 3 H)
1.12 (m, 1 H)
0.65-0.57 (m, 2 H)
-0.03 (s, 9 H)
-0.32 (m, 1 H)

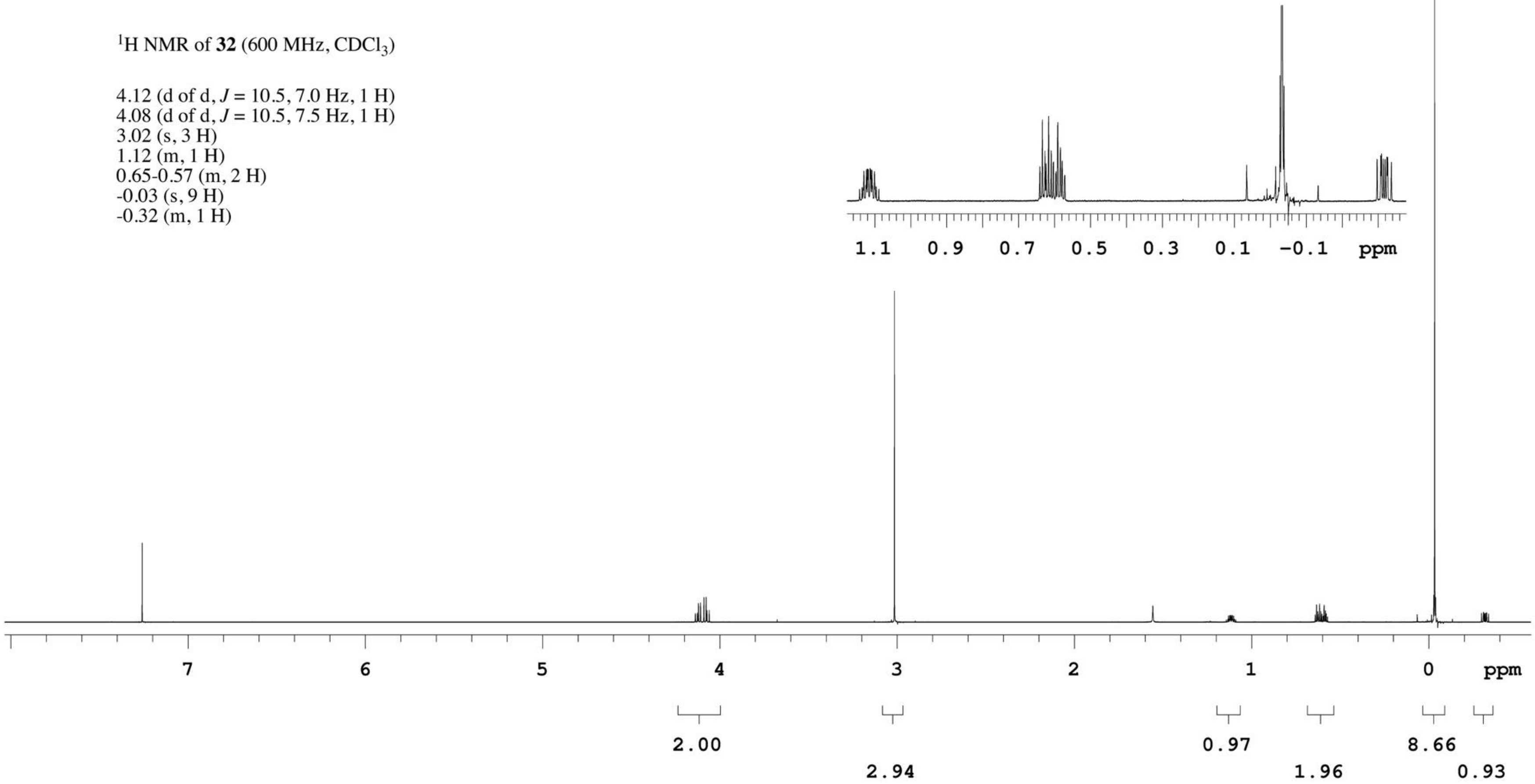

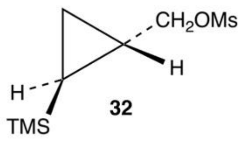

32

^{13}C NMR of **32** (150 MHz, $CDCl_3$)

76.4
37.8
14.1
8.1
3.5
-2.5

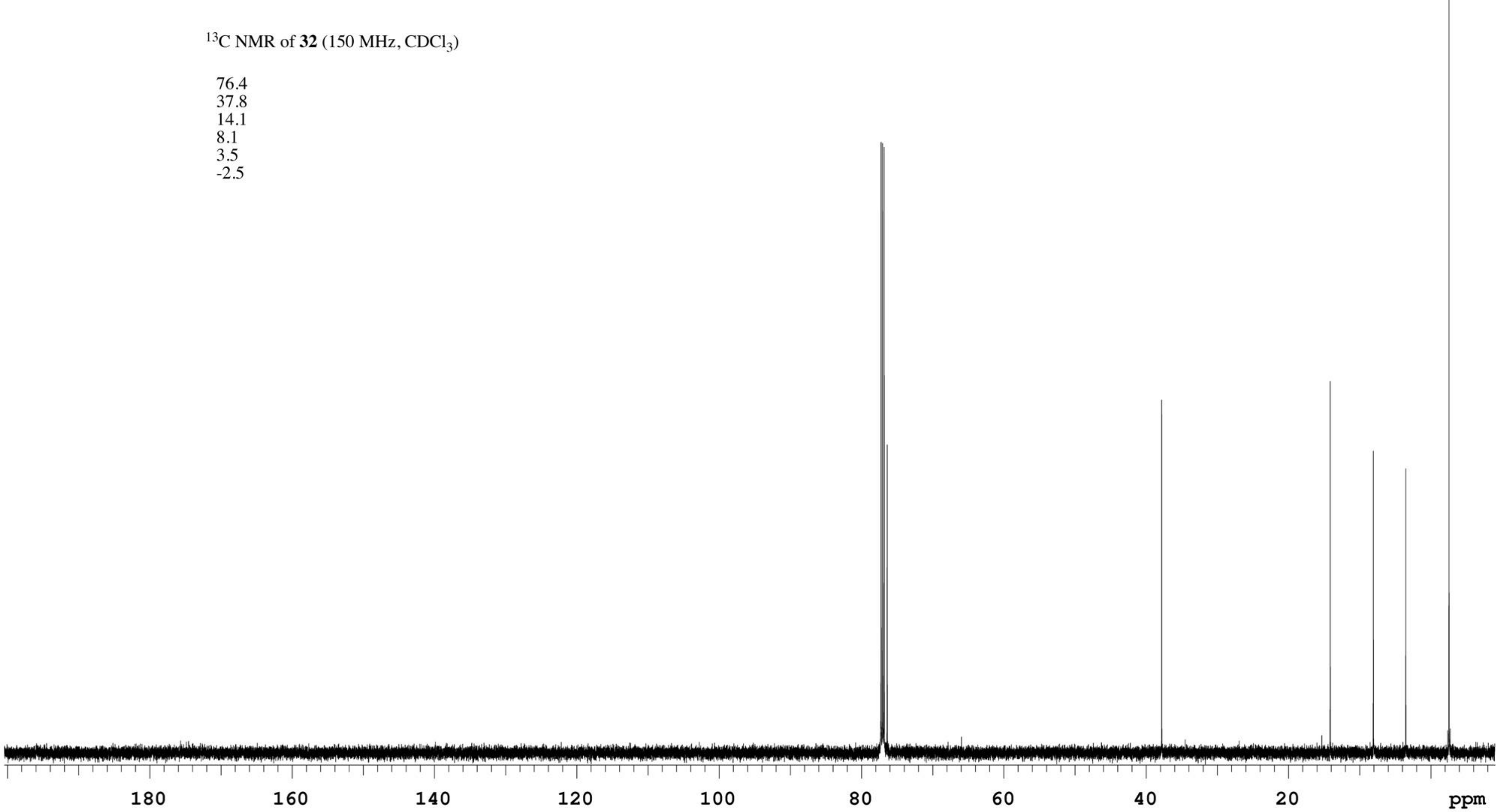

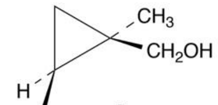

A

^{1}H NMR of **A** (600 MHz, DMSO-d_6)

4.48 (d of d, J = 5.9, 4.5 Hz, 1 H)
3.27 (d of d of d, J = 10.8, 5.9, 0.8 Hz, 1 H)
3.20 (d of d, J = 10.8, 4.5 Hz, 1 H)
1.10 (s, 3 H)
0.50 (d of d of d, J = 9.9, 3.3, 0.8 Hz, 1 H)
0.35 (d of d, J = 7.4, 3.3 Hz, 1 H)
-0.01 (s, 9 H)
-0.53 (d of d, J = 9.8, 7.4 Hz, 1 H)

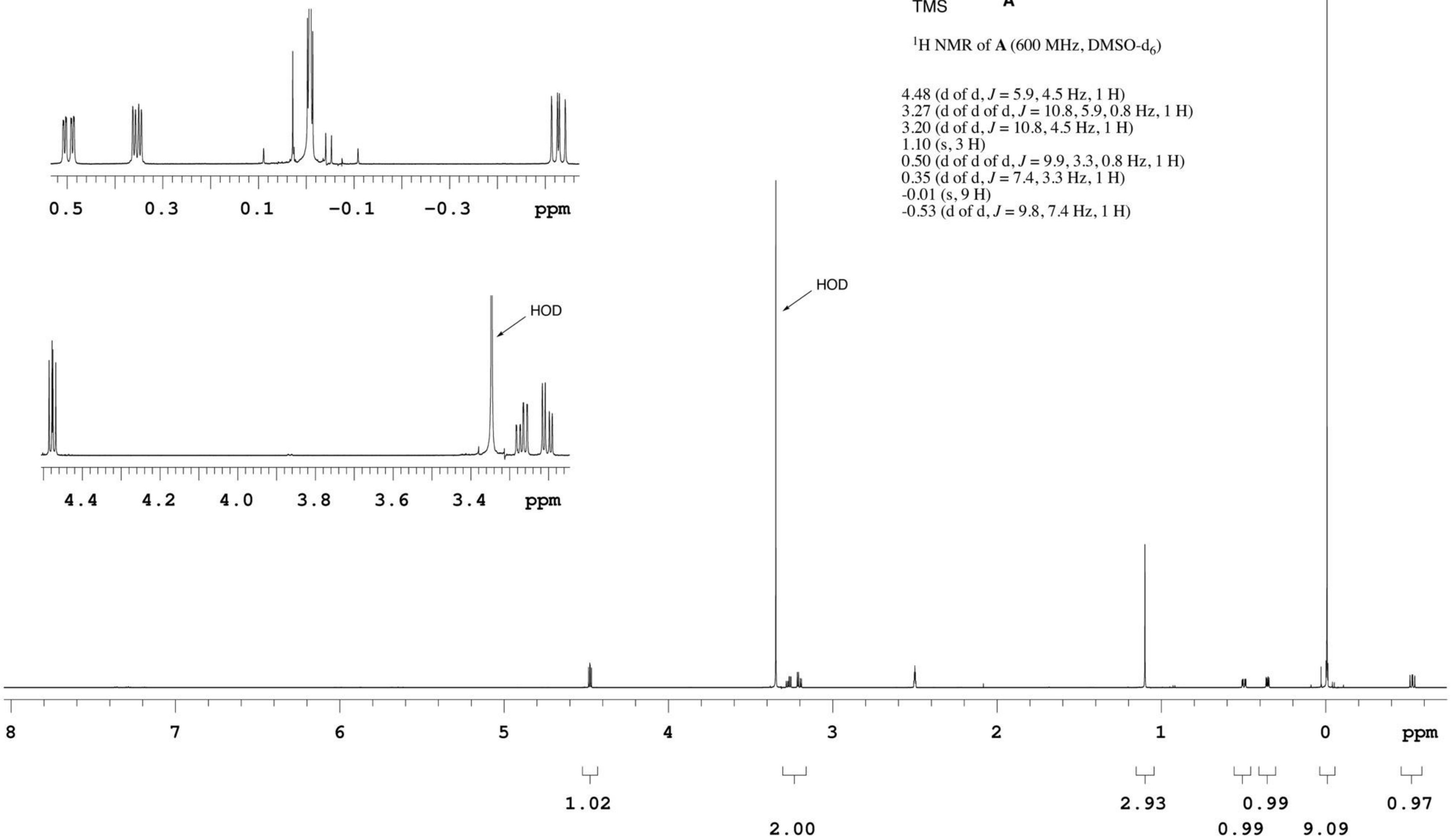

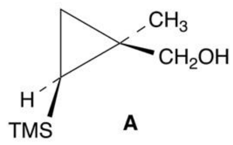

A

^{13}C NMR of **A** (150 MHz, DMSO-d_6)

66.7
23.9
23.6
15.8
11.2
0.0

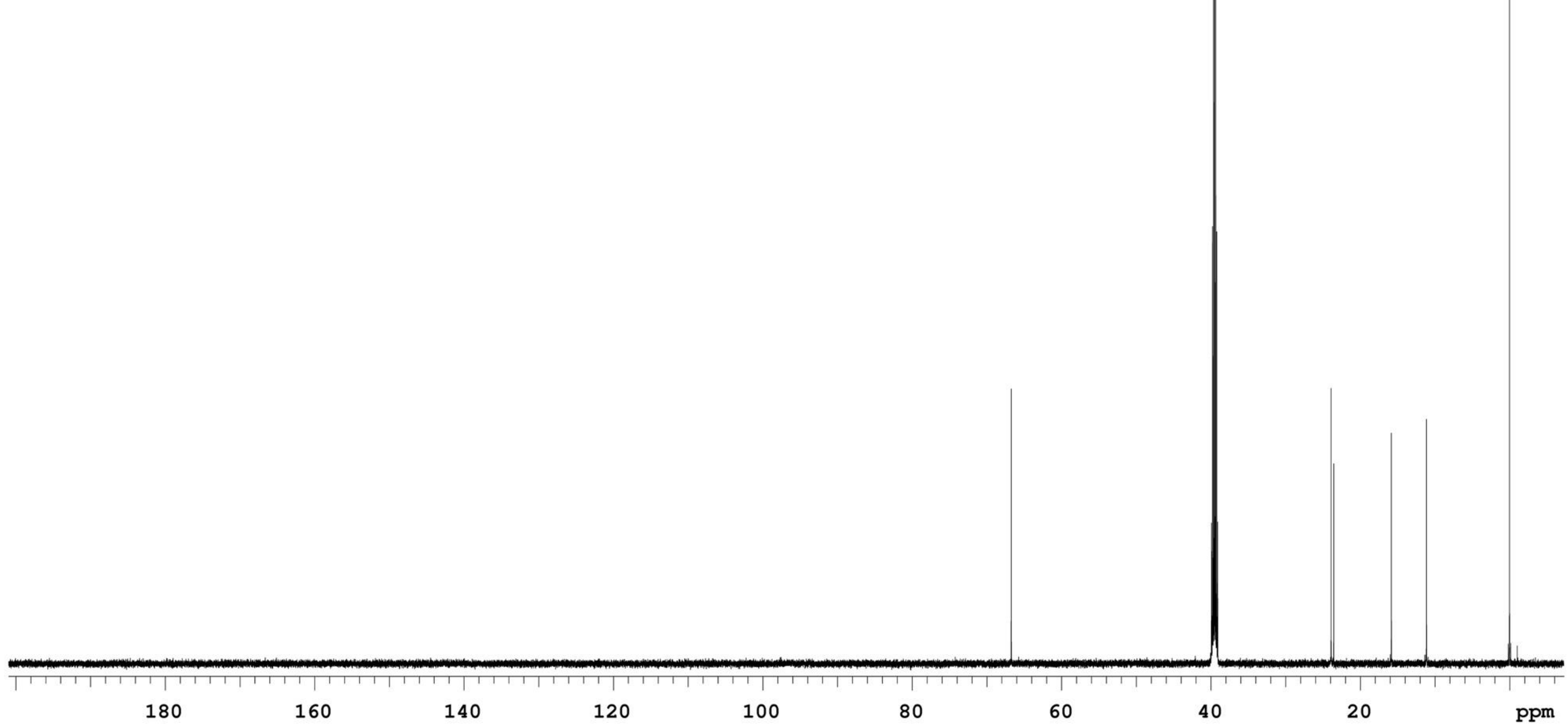

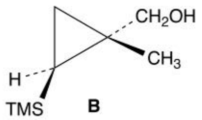

B

^{1}H NMR of **B** (600 MHz, $CDCl_3$)

3.35 (s, 2 H)
1.18 (s, 3 H)
0.65 (d of d, J = 10.4, 3.7 Hz, 1 H)
0.34 (d of d, J = 7.2, 3.7 Hz, 1 H)
0.04 (s, 9 H)
-0.42 (d of d, J = 10.5, 7.2 Hz, 1 H)

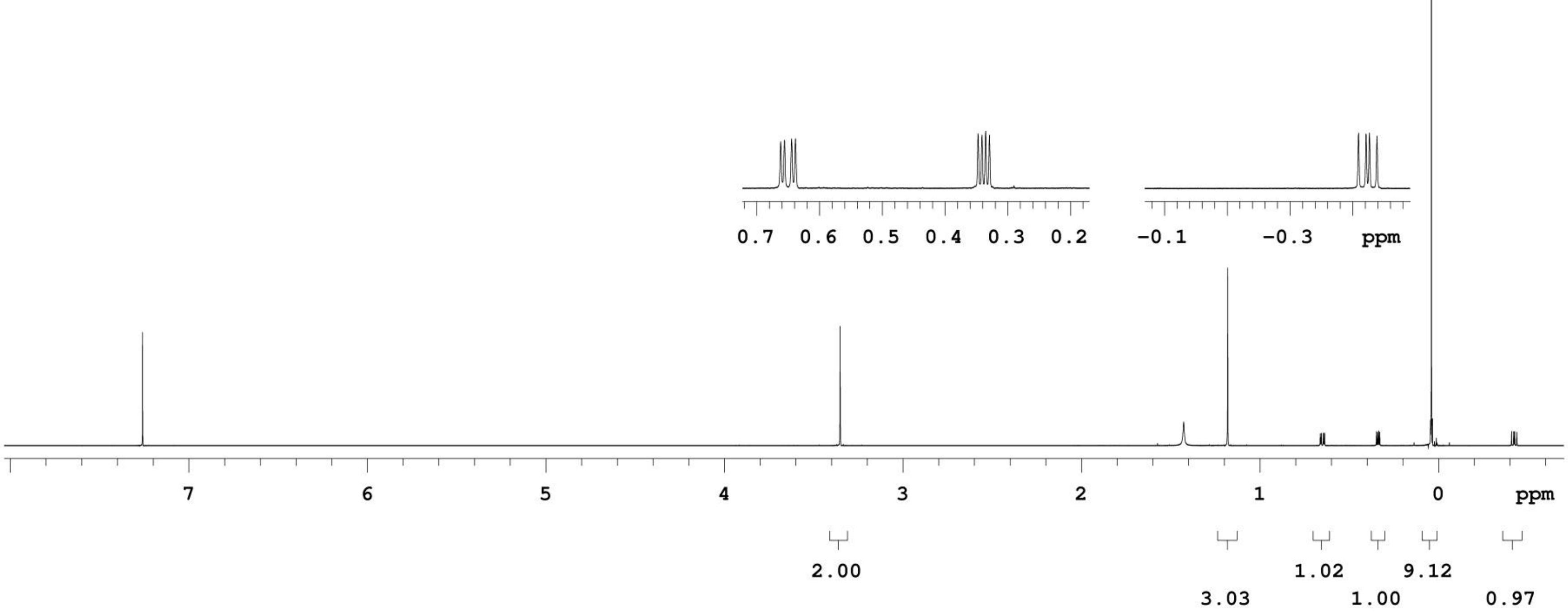

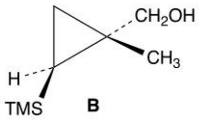

B

^{13}C NMR of **B** (150 MHz, $CDCl_3$)

73.6
23.4
18.3
15.3
9.2
-0.3

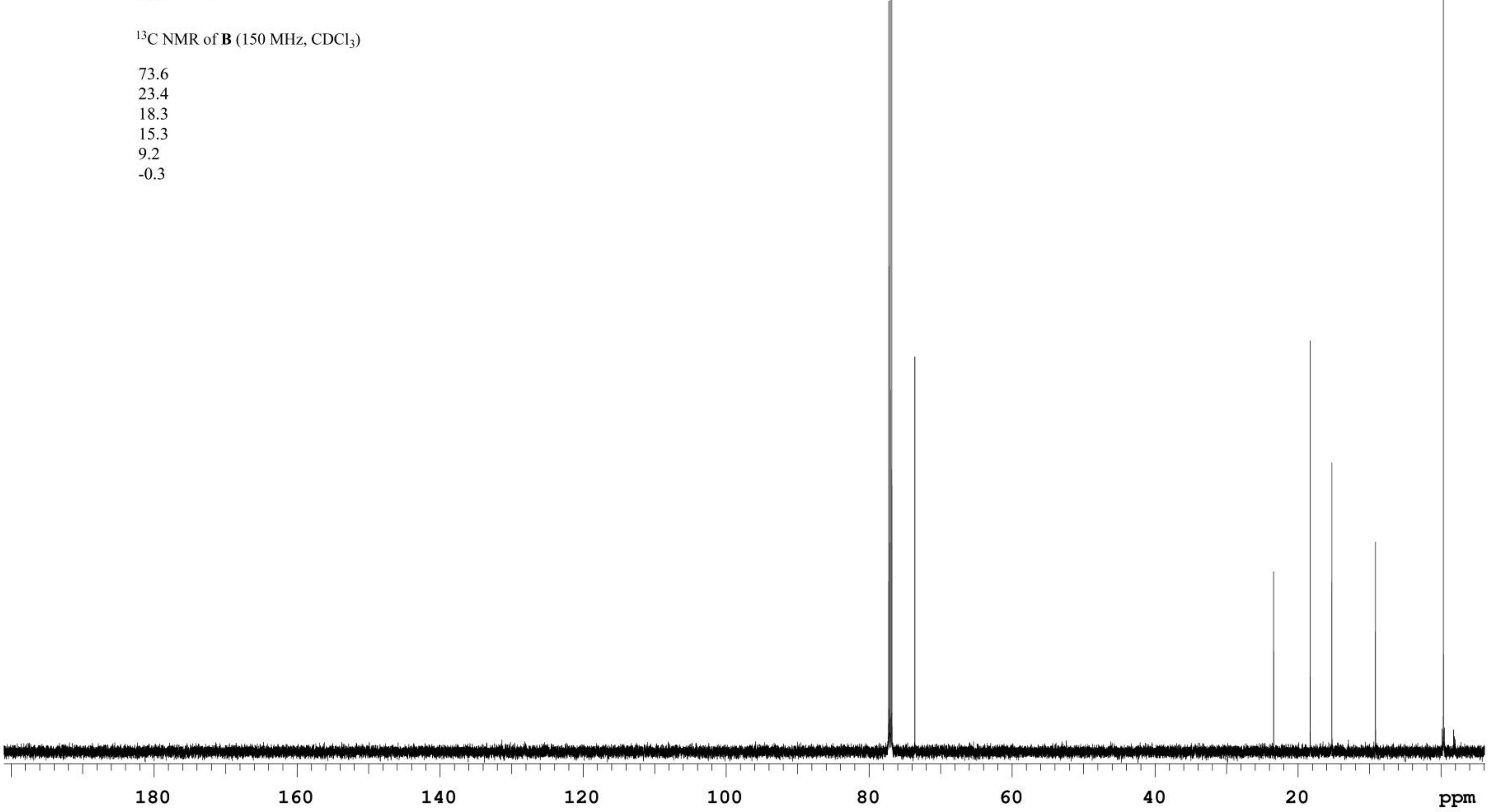

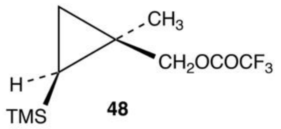

48

^{1}H NMR of **48** (600 MHz, $CDCl_3$)

4.37 (d, J = 10.8 Hz, 1 H)
4.05 (d, J = 10.8 Hz, 1 H)
1.21 (s, 3 H)
0.74 (d of d, J = 10,2, 4.2 Hz, 1 H)
0.54 (d of d, J = 7.8, 4.2 Hz, 1 H)
0.04 (s, 9 H)
-0.27 (d of d, J = 9.6, 7.8 Hz, 1 H)

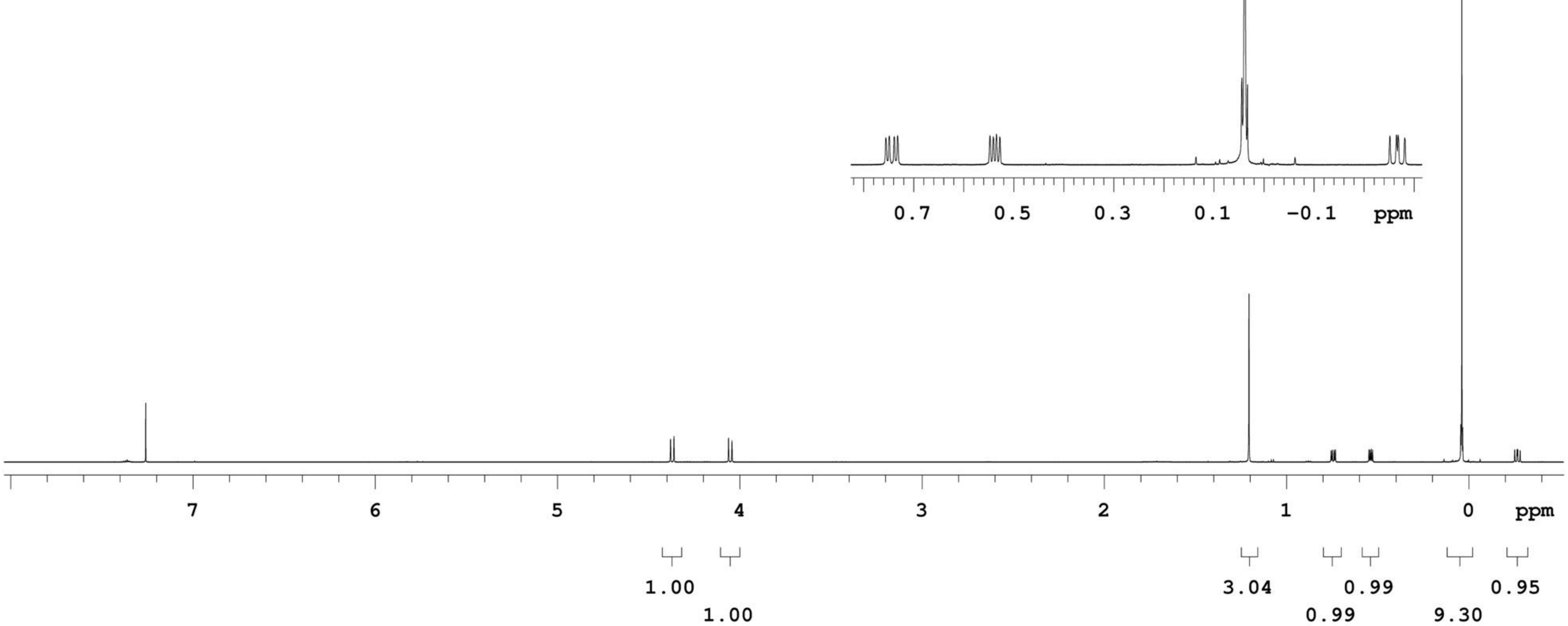

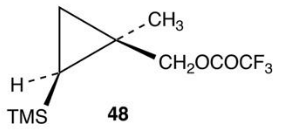

48

^{13}C NMR of **48** (150 MHz, $CDCl_3$)

157.7 (q, J = 42 Hz)
114.7 (q, J = 286 Hz)
74.9
23.8
20.5
16.3
12.8
-0.6.

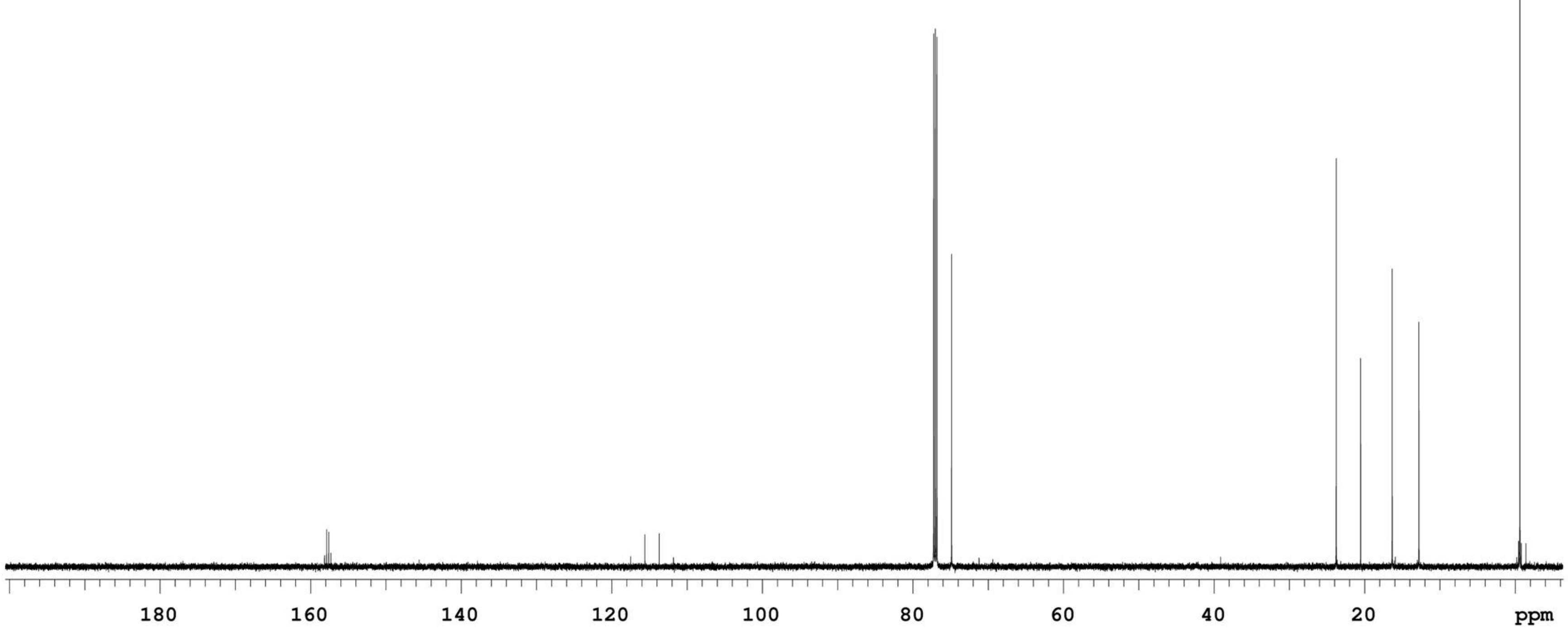

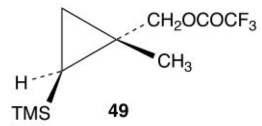

49

^{1}H NMR of **49** (600 MHz, $CDCl_3$)

4.17 (d, *J* = 10.8 Hz, 1 H)
4.09 (d, *J* = 10.8 Hz, 1 H)
1.18 (s, 3 H)
0.81 (d of d, *J* = 10.6, 4.1 Hz, 1 H)
0.46 (d of d, *J* = 7.6, 4.2 Hz, 1 H)
0.05 (s, 9 H)
-0.26 (d of d, *J* = 10.7, 7.6 Hz, 1 H)

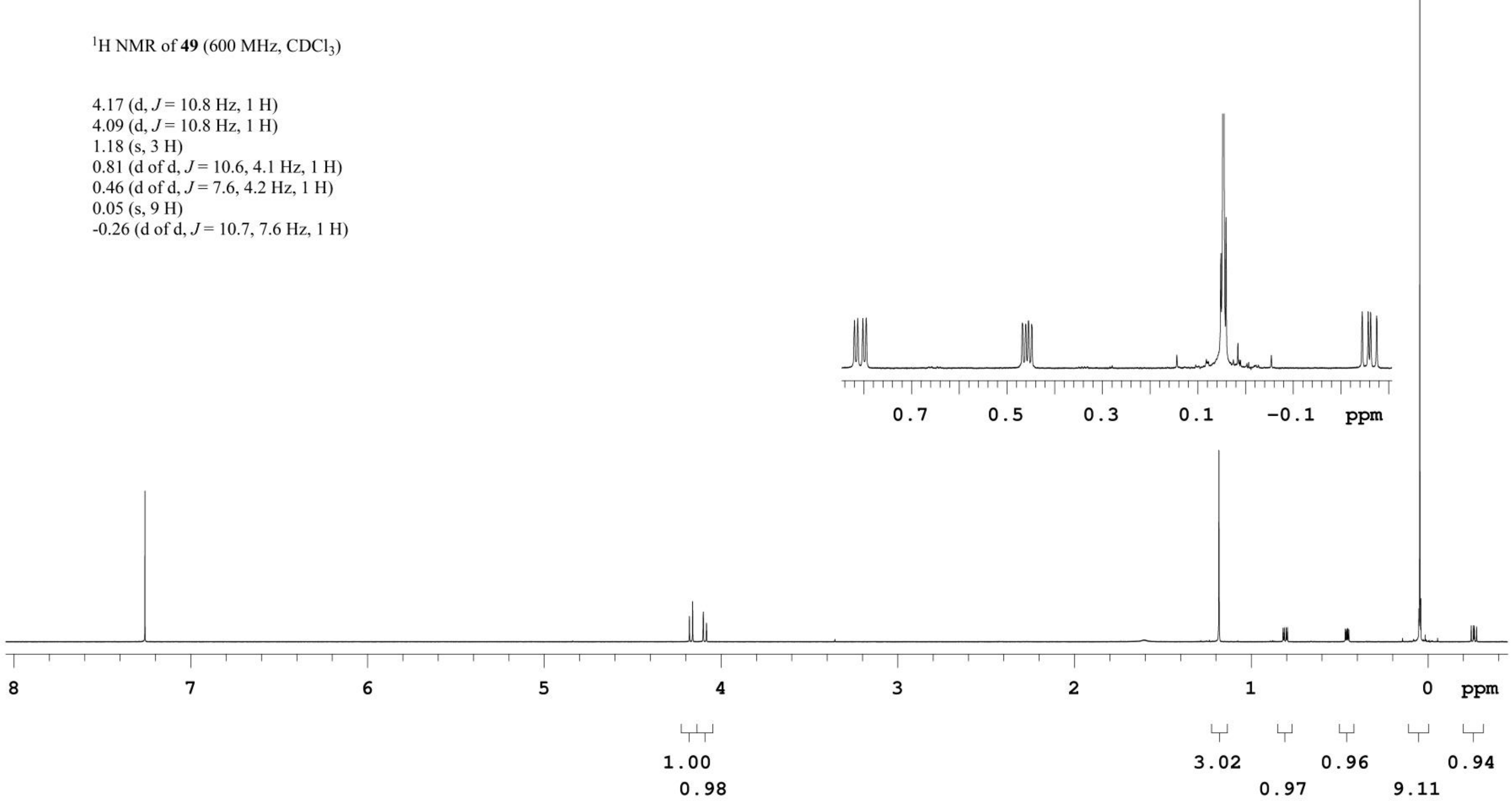

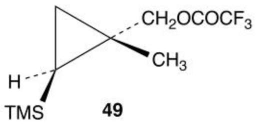

49

^{13}C NMR of **49** (150 MHz, $CDCl_3$)

157.7 (q, J = 42 Hz)
114.7 (q, J = 286 Hz)
78.6
20.0
18.4
15.9
10.4
-0.6

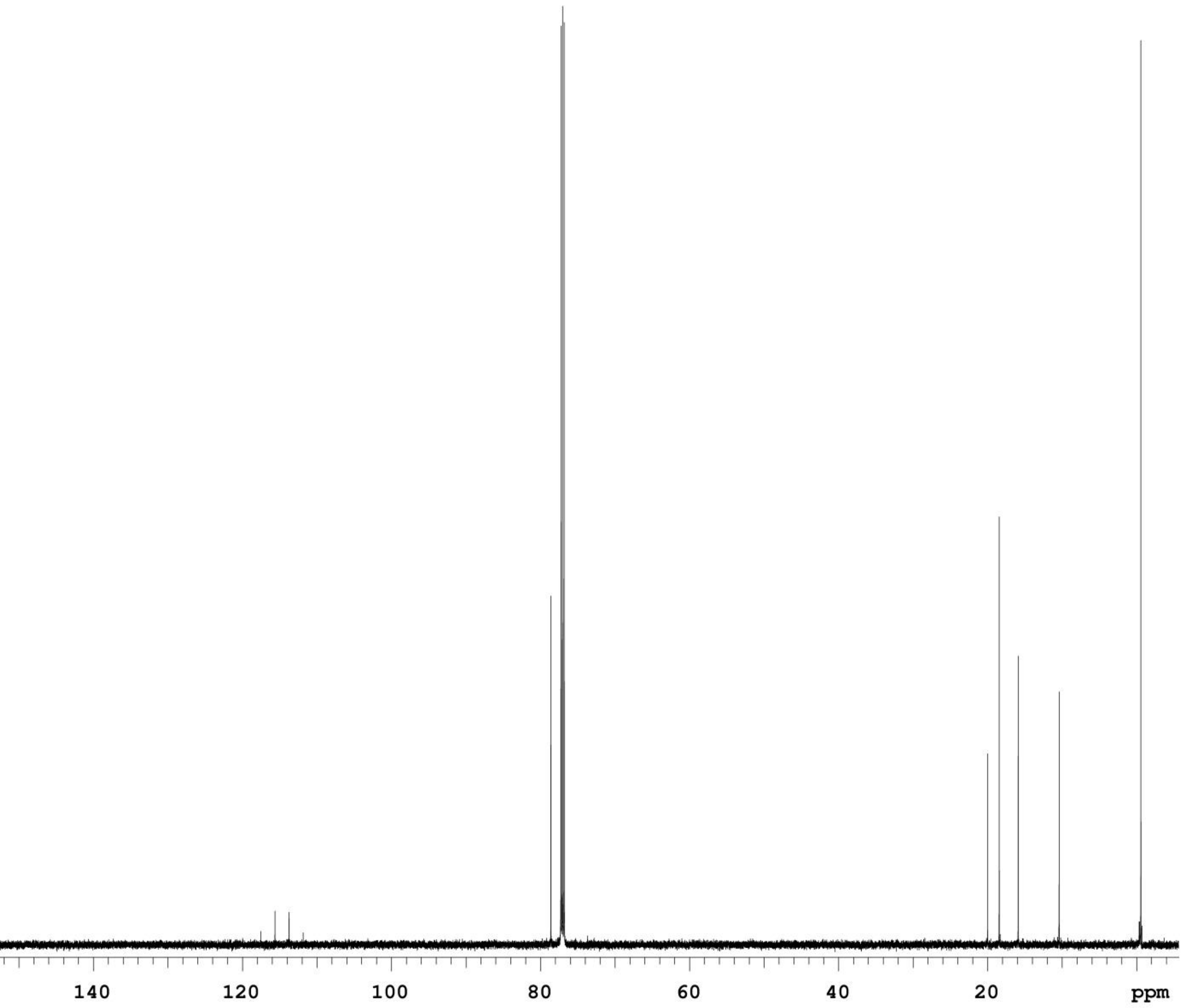

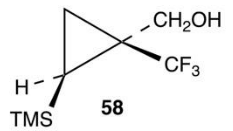

58

^{1}H NMR of **58** (600 MHz, $CDCl_3$)

3.85 (d of d, J = 12.4, 6.4 Hz, 1 H)
3.54 (d of d, J = 12.4, 6.6 Hz, 1 H)
1.72 (t, J = 6.5 Hz, 1 H)
0.99-0.90 (m, 2 H)
0.11-0.06 (m, 10 H)

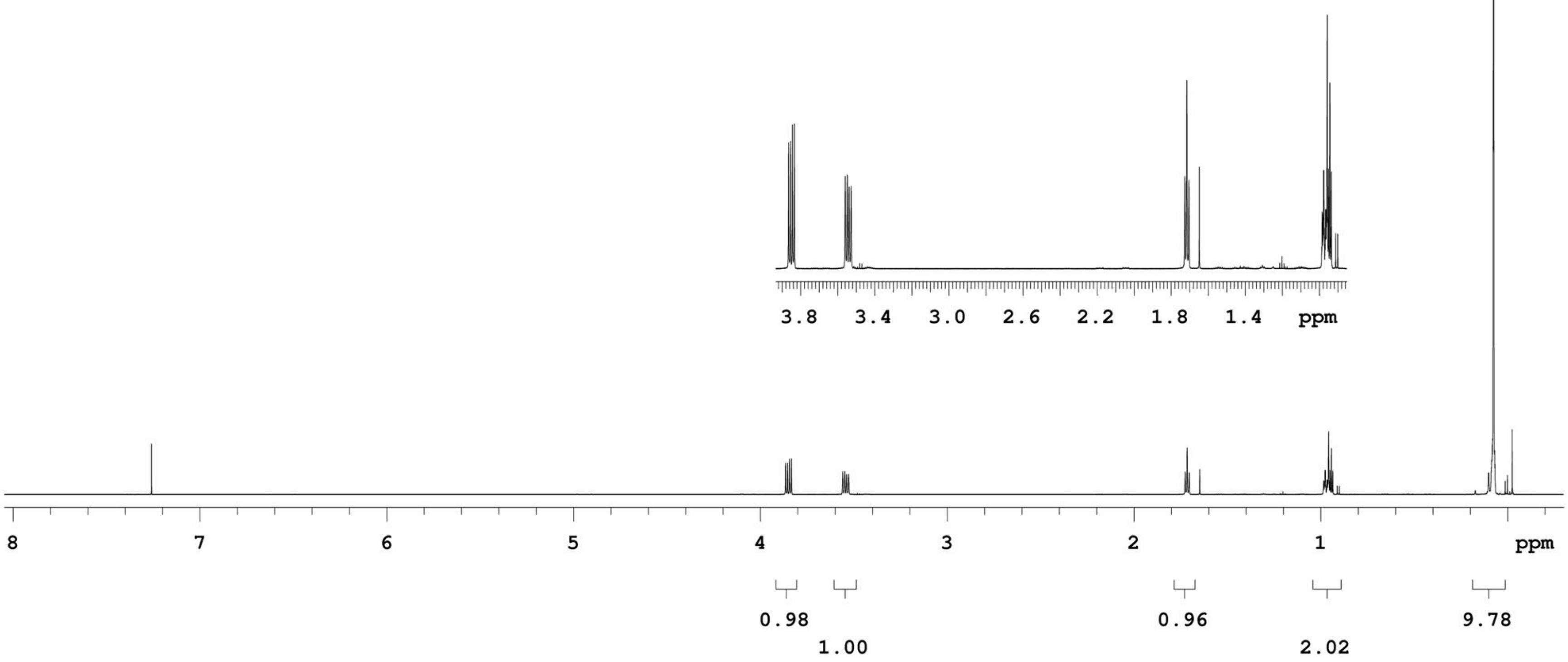

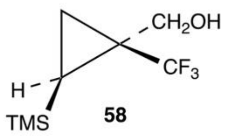

58

^{13}C NMR of **58** (150 MHz, $CDCl_3$)

127.9 (q, J = 275 Hz)
66.3 (q, J = 1.8 Hz)
30.1 (q, J = 31.3 Hz)
11.9 (q, J = 2.2 Hz)
9.7 (q, J = 1.2 Hz)
-1.0 (q, J = 2.1 Hz)

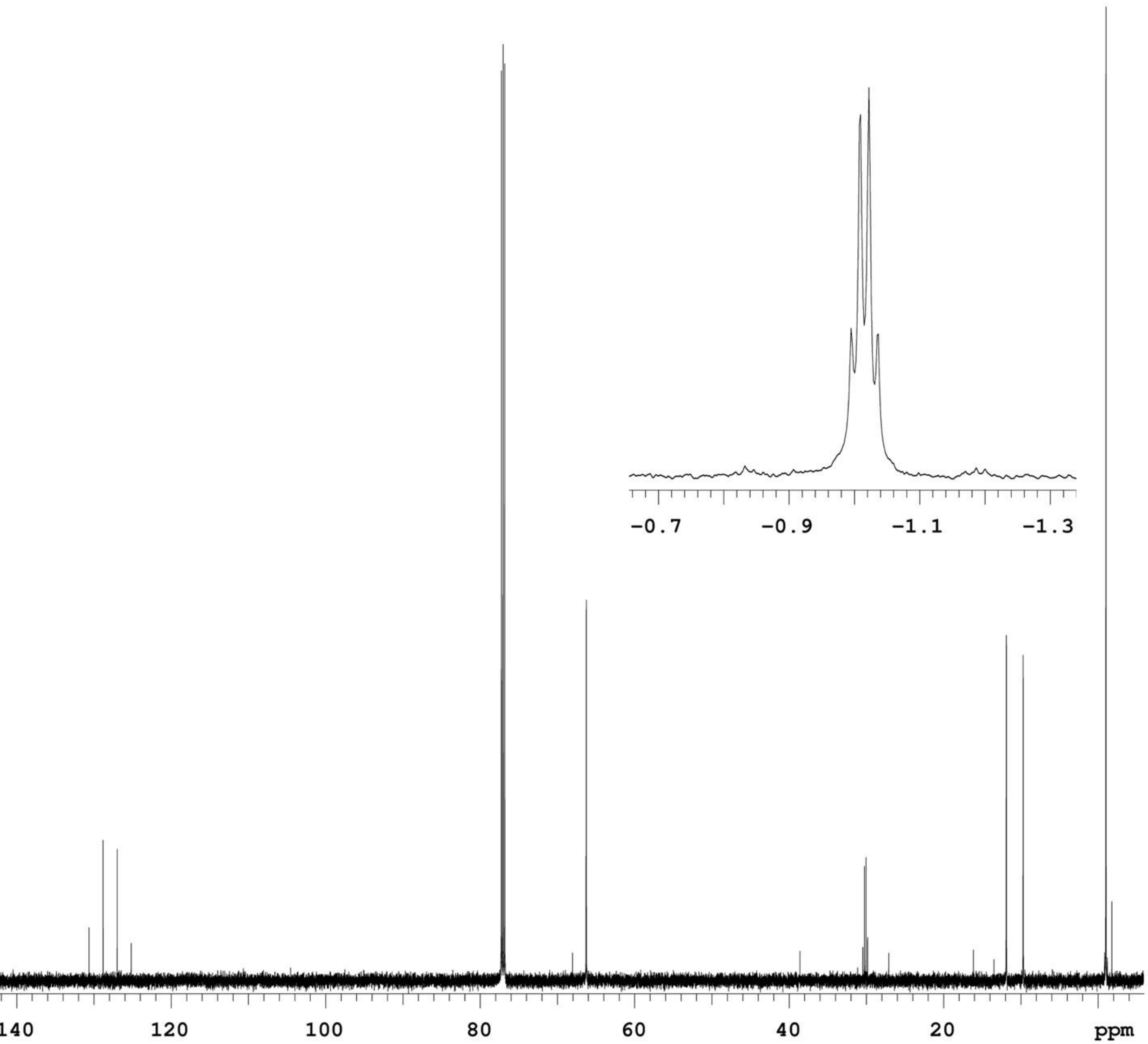

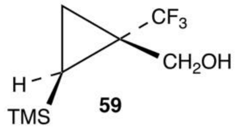

59

^{1}H NMR of **59** (600 MHz, $CDCl_3$)

3.86 (d of d, J = 12.9, 6.0 Hz, 1 H)
3.65 (d of d, J = 12.9, 6.8 Hz, 1 H)
1.59 (t of q, J = 6.4, 0.9 Hz, 1 H)
1.23 (d of d, J = 11.0, 4.5 Hz, 1 H)
0.67 (m, 1 H)
0.30 (d of d, J = 11.0, 8.6 Hz, 1 H)
0.10 (s, 9 H)

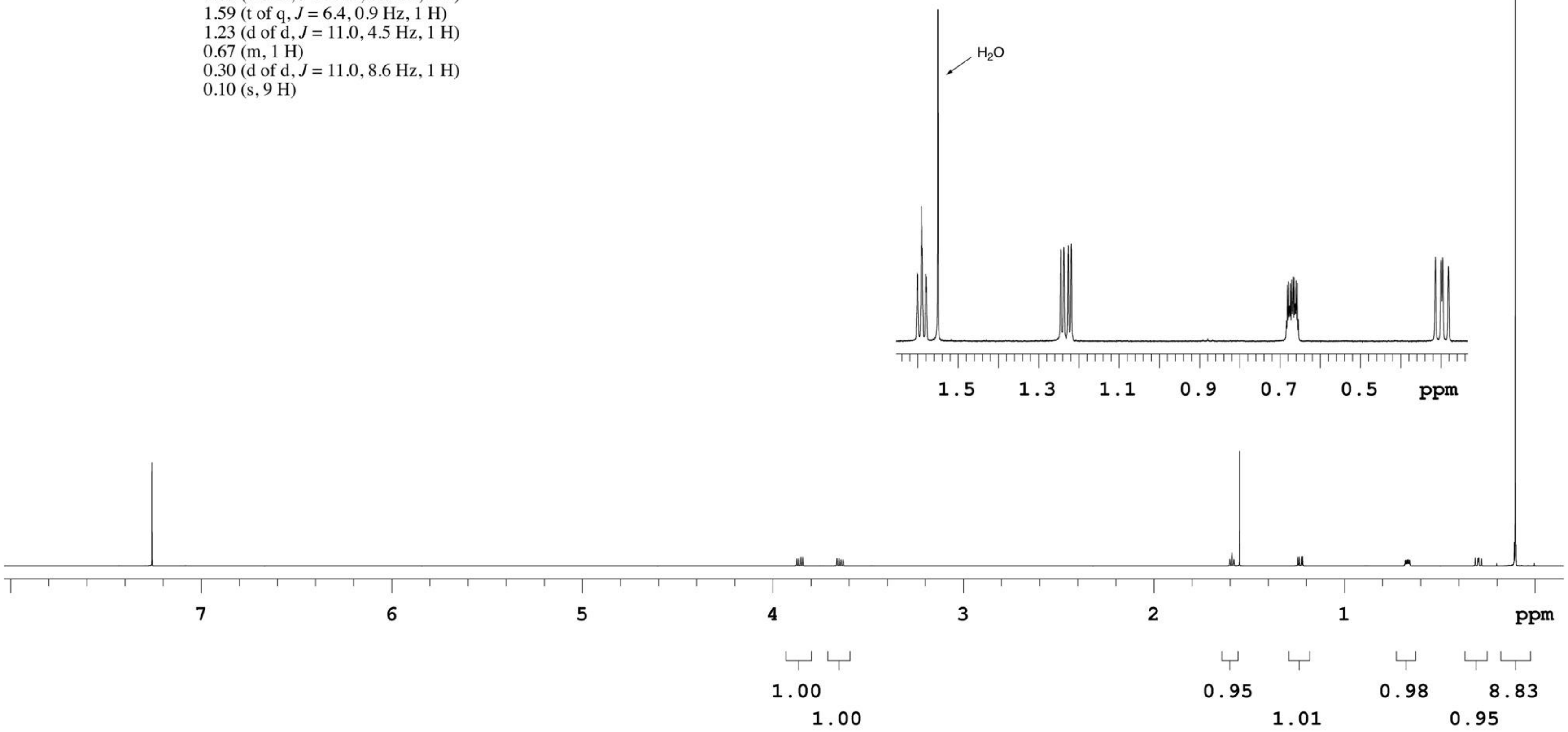

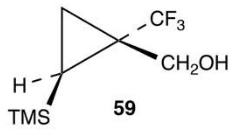

59

^{13}C NMR of **59** (150 MHz, $CDCl_3$)

127.5 (q, J = 275 Hz)
62.6 (q, J = 1.2 Hz)
29.0 (q, J = 30.4 Hz)
11.6 (q, J = 2.5 Hz)
7.8
-0.9.

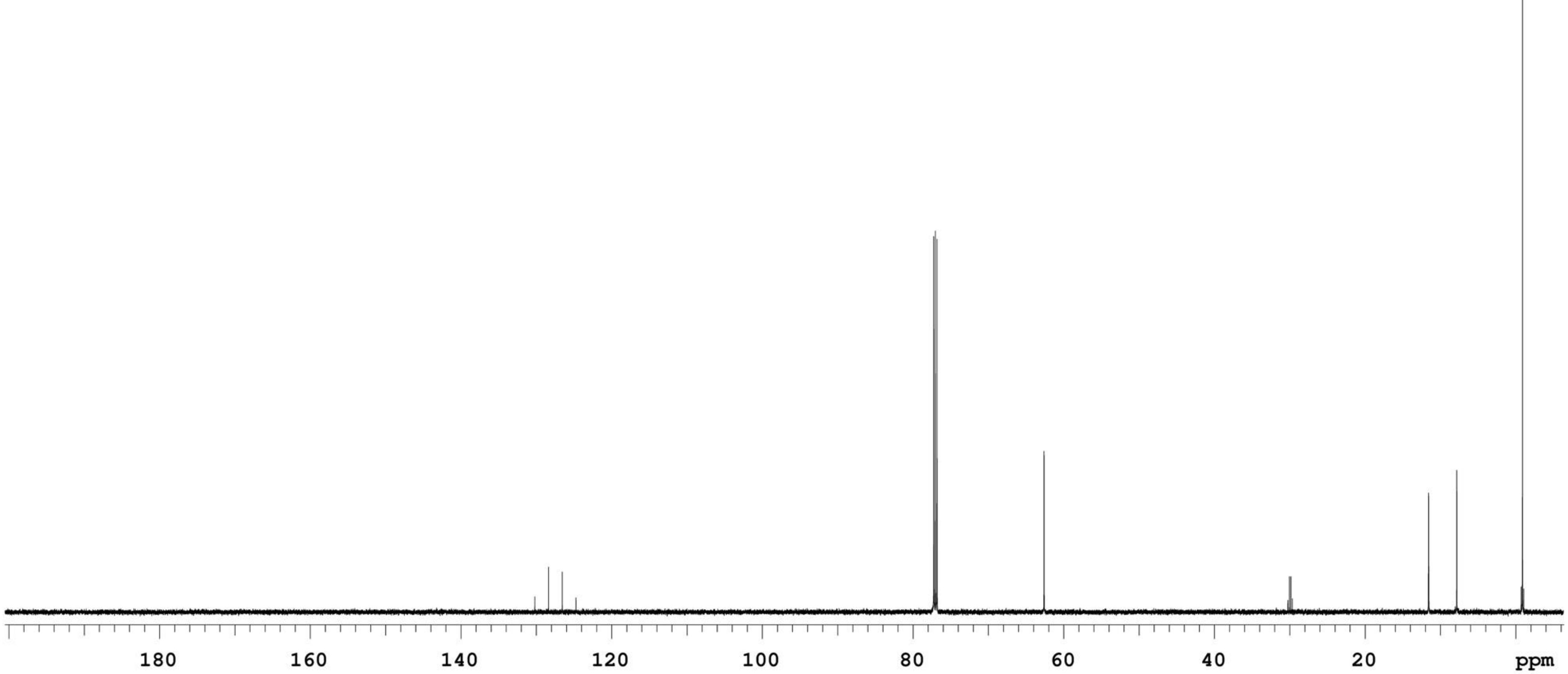

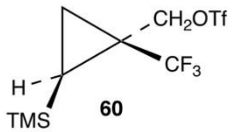

60

^{1}H NMR of **60** (600 MHz, $CDCl_3$)

4.71 (d, J = 11.5 Hz, 1 H)
4.35 (d, J = 11.5 Hz, 1 H)
1.21-1.15 (m, 2 H)
0.26 (t of m, J = 10.3 Hz, 1 H)
0.11 (q, J = 0.9 Hz, 9 H)

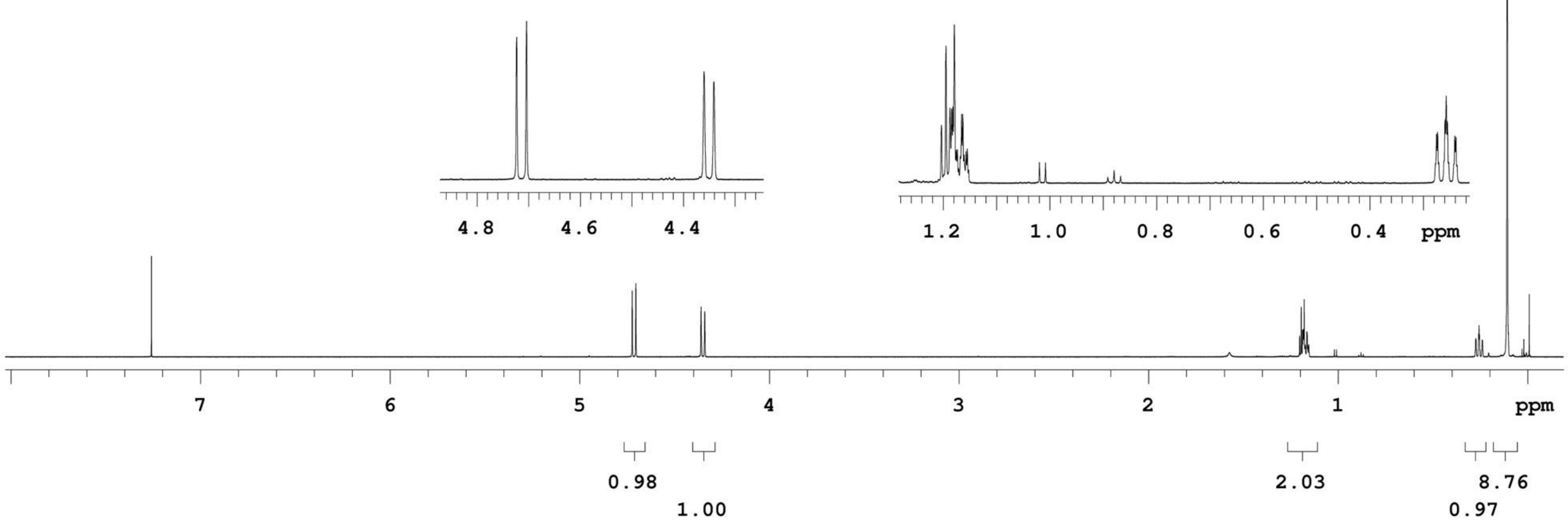

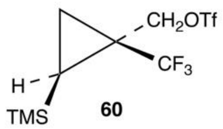

60

^{13}C NMR of **60** (150 MHz, $CDCl_3$)

126.2 (q, J = 275 Hz)
118.6 (q, J = 319 Hz)
79.9
27.8 (q, J = 34.4 Hz)
13.6 (q, J = 1.8 Hz)
12.2
-1.2 (q, J = 2.0 Hz)

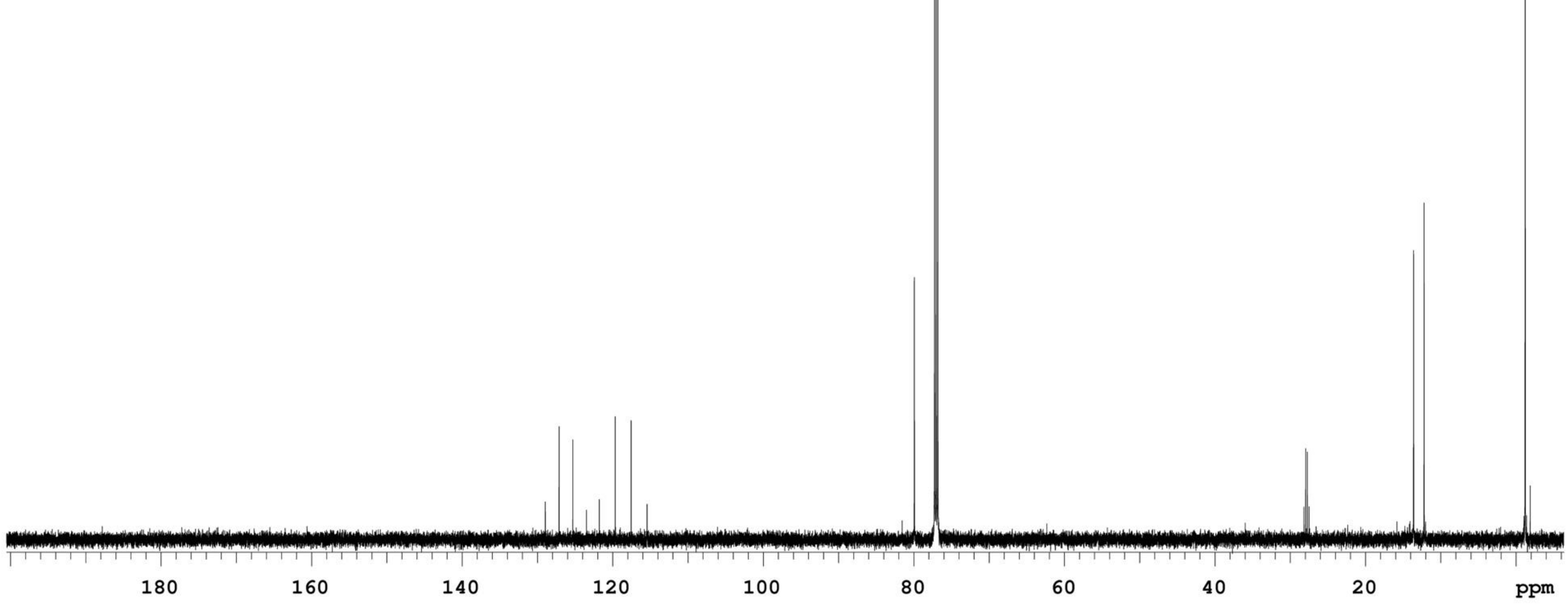

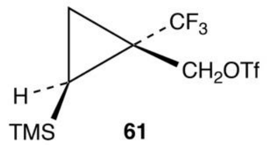

61

^{1}H NMR of **61** (600 MHz, $CDCl_3$)

4.71 (d, J = 11.6 Hz, 1 H)
4.50 (d, J = 11.6 Hz, 1 H)
1.47 (d of d, J = 11.2, 5.0 Hz, 1 H)
0.86 (m, 1 H)
0.53 (d of d, J = 11.1, 9.1 Hz, 1 H)
0.14 (s, 9 H)

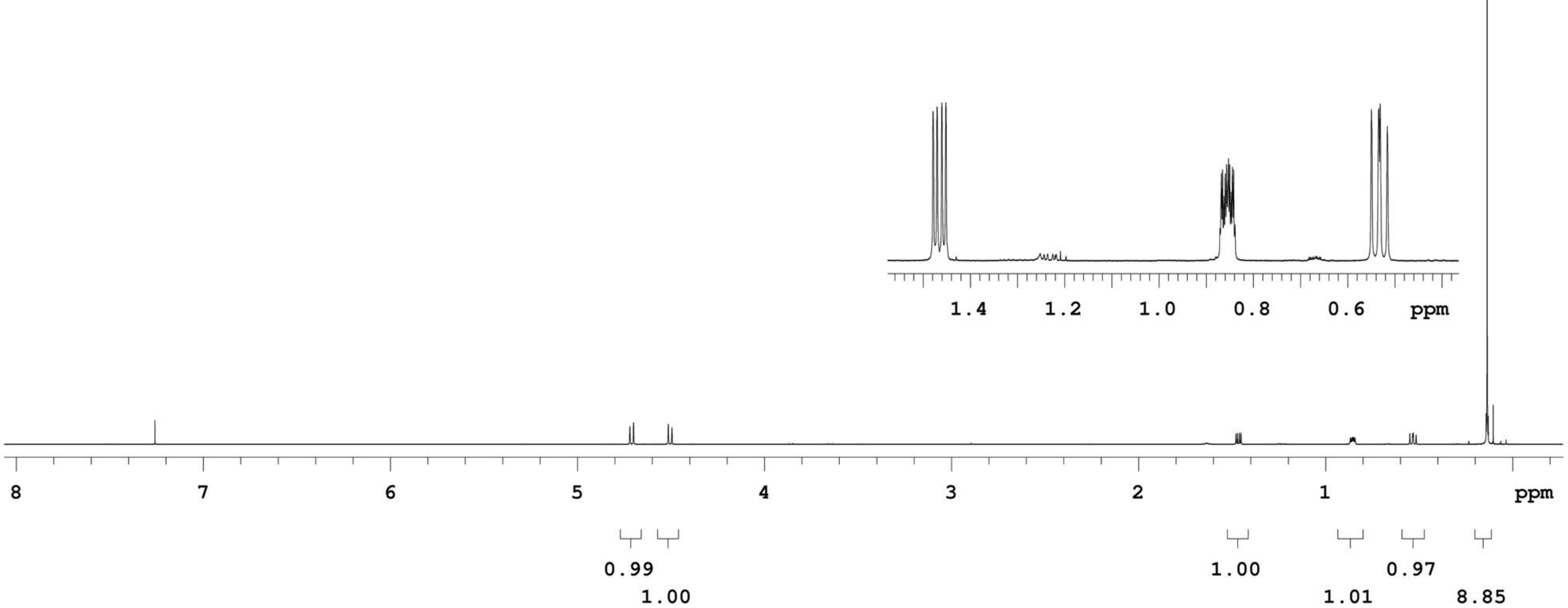

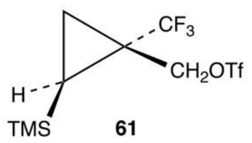

61

^{13}C NMR of **61** (150 MHz, $CDCl_3$)

125.7 (q, J = 275 Hz)
118.6 (q, J = 319 Hz)
76.2
27.5 (q, J = 33.5 Hz)
12.8 (q, J = 1.9 Hz)
9.6,
-1.2.

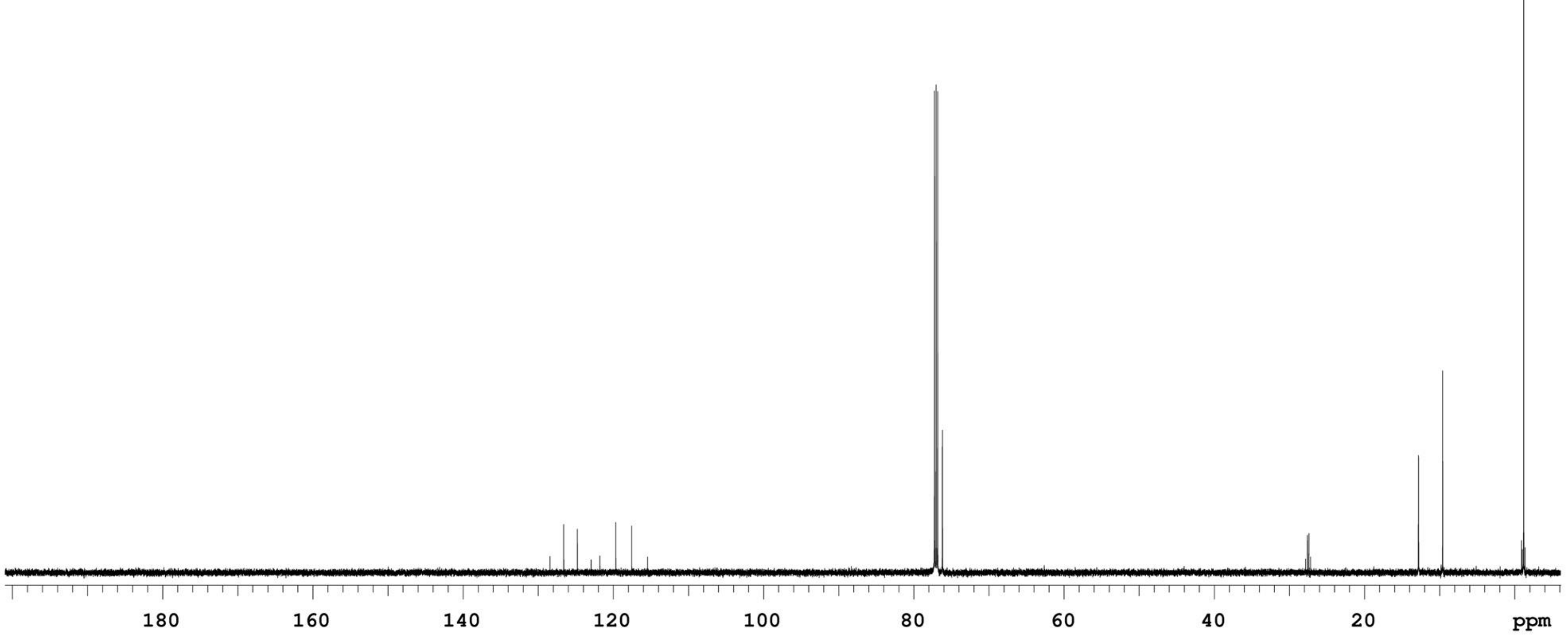

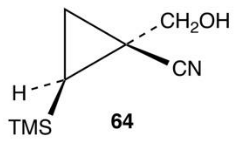

64

^{1}H NMR of **64** (600 MHz, $CDCl_3$)

3.63 (d of d, J = 11.5, 5.6 Hz, 1 H)
3.60 (d of d, J = 11.5, 5.6 Hz, 1 H)
1.97 (t, J = 6.1 Hz, 1 H)
1.18-1.12 (m, 2 H)
0.18 (d of d, J = 10.4, 8.8 Hz, 1 H)
0.16 (s, 9 H)

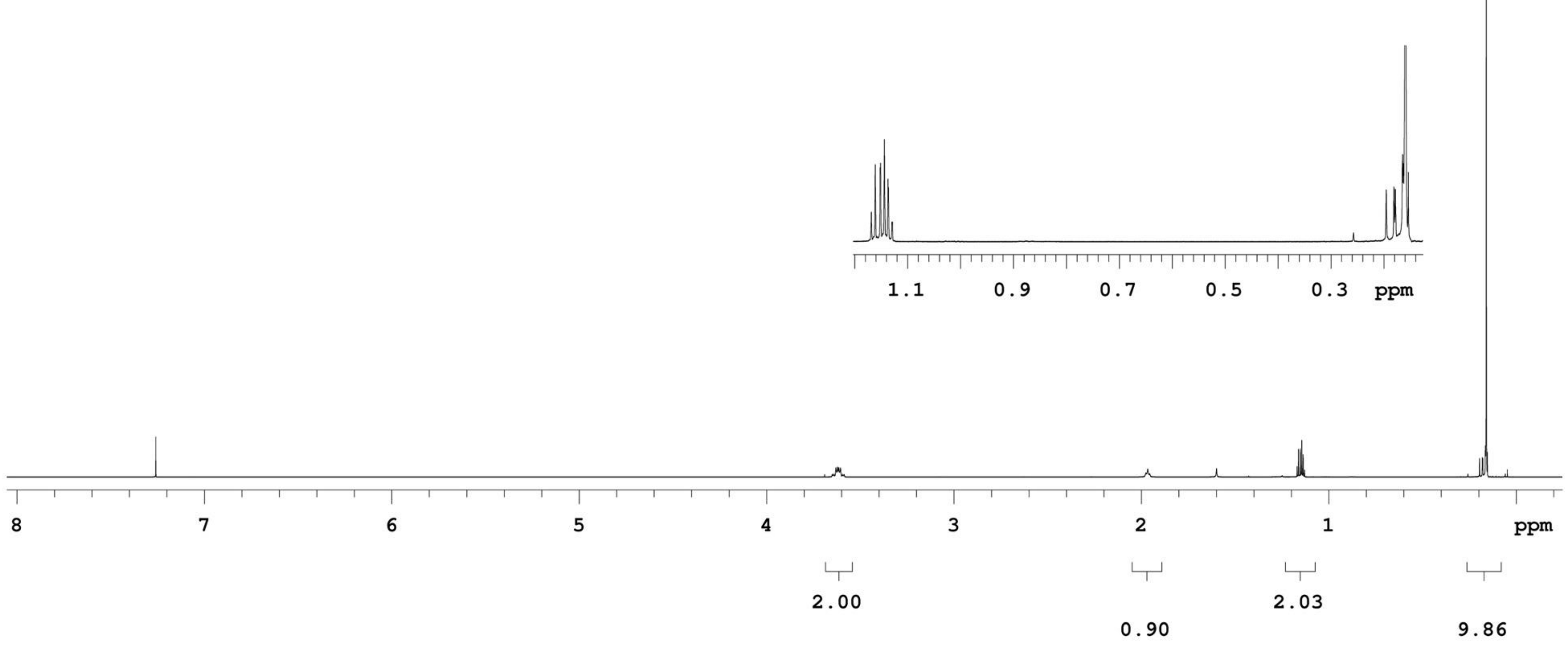

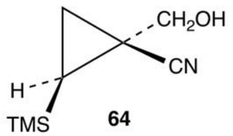

64

^{13}C NMR of **64** (150 MHz, $CDCl_3$)

122.0
68.0
16.7
15.9
11.7
-2.1

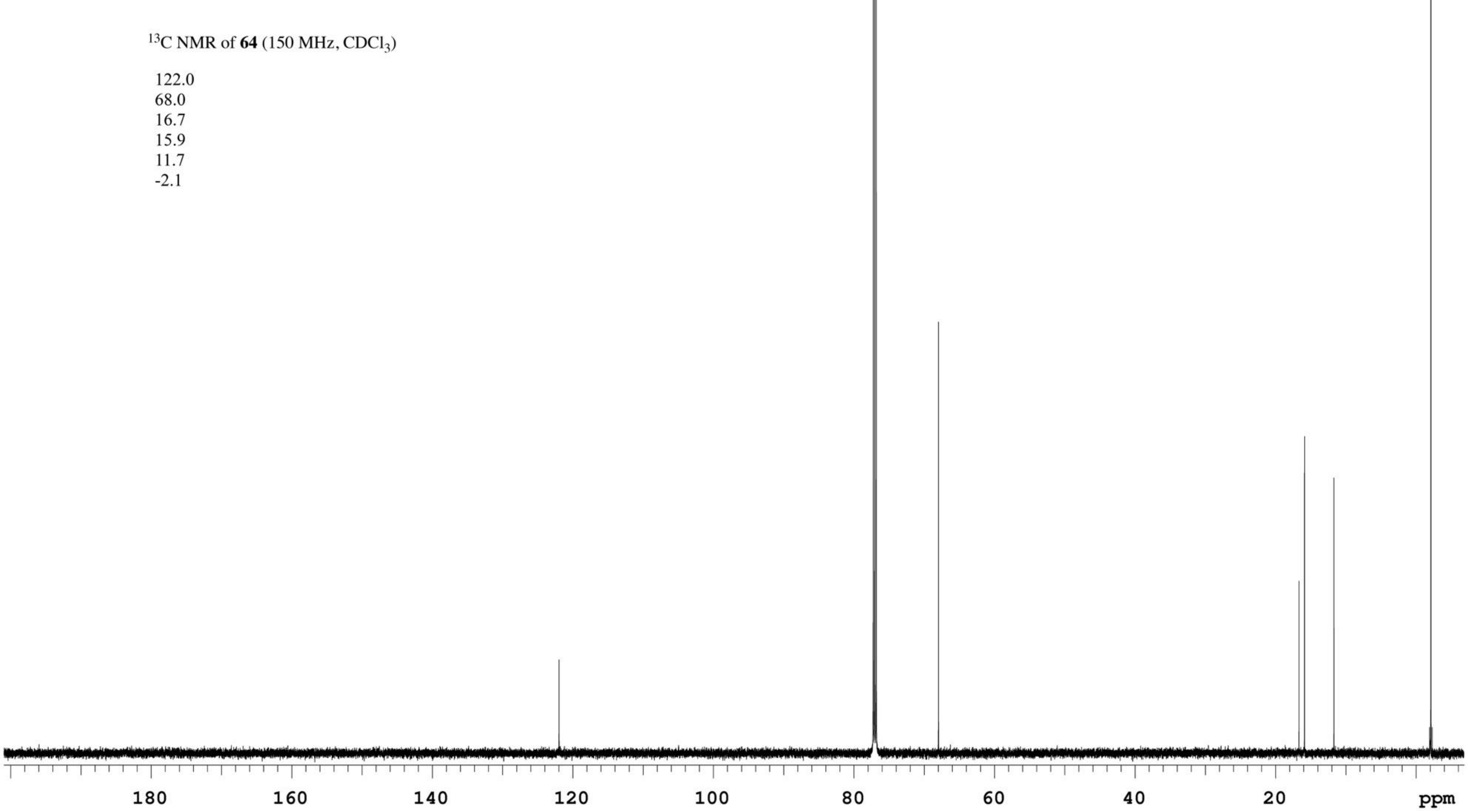

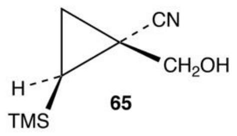

65

^{1}H NMR of **65** (600 MHz, $CDCl_3$)

3.66 (d of d, J = 11.8, 6.5 Hz, 1 H)
3.61 (d of d, J = 11.8, 5.8 Hz, 1 H)
2.08 (t, J = 6.2 Hz, 1 H)
1.54 (d of d of d, J = 11.0, 4.4, 0.7 Hz, 1 H)
0.87 (d of d, J = 9.1, 4.5 Hz, 1 H)
0.56 (d of d, J = 11.0, 9.1 Hz, 1 H)
0.10 (s, 9 H)

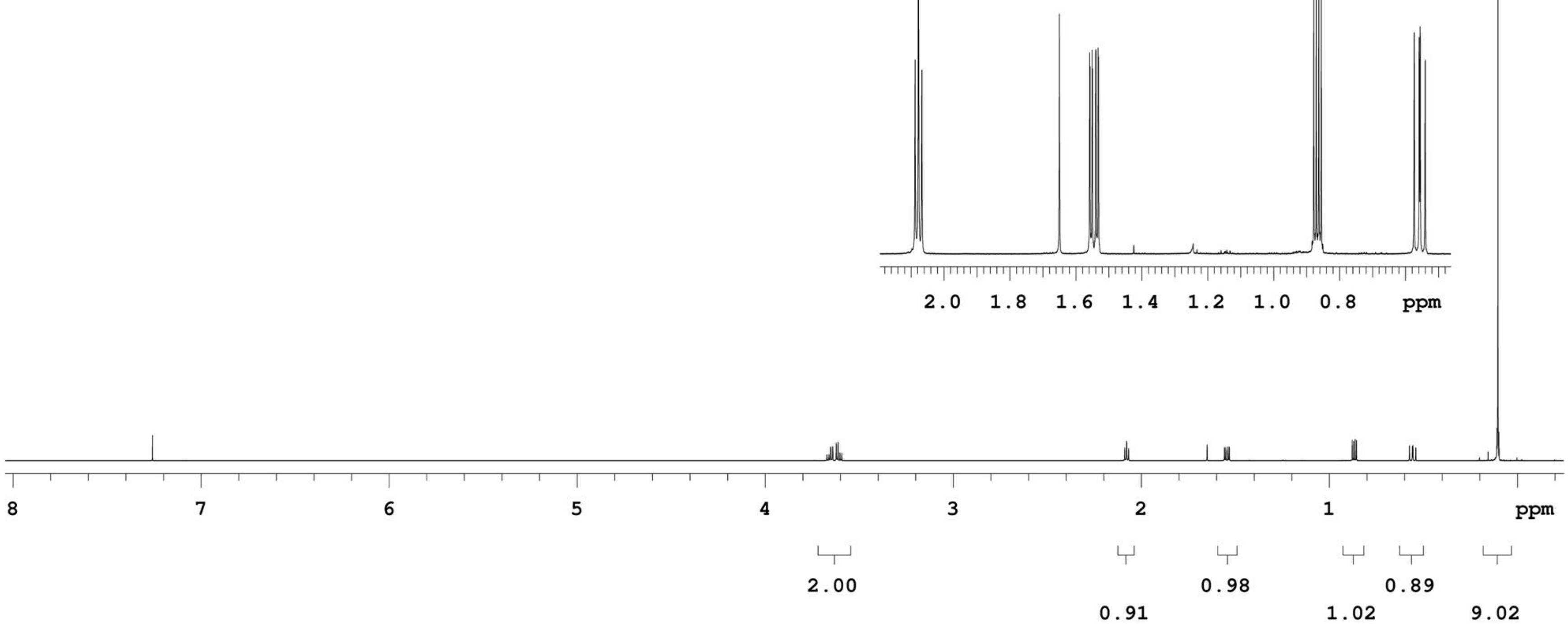

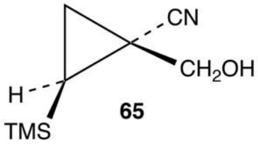

65

^{13}C NMR of **65** (150 MHz, $CDCl_3$)

123.5
64.4
17.5
16.2
13.7
-0.8

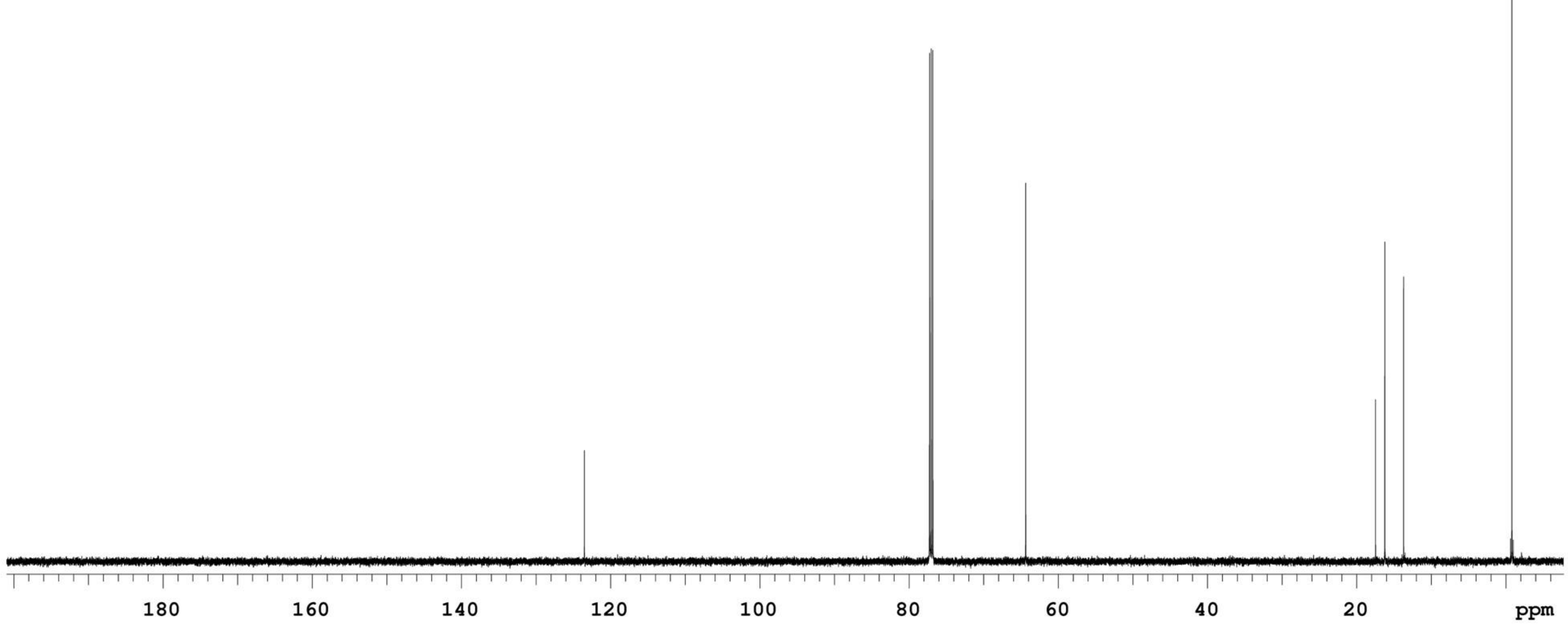

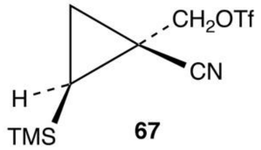

67

^{1}H NMR of **67** (600 MHz, $CDCl_3$)

4.53 (d, J = 10.9 Hz, 1 H)
4.35 (d, J = 10.9 Hz, 1 H)
1.43-1.37 (m, 2 H)
0.41 (d of d, J = 10.7, 9.4 Hz, 1 H)
0.19 (s, 1 H)

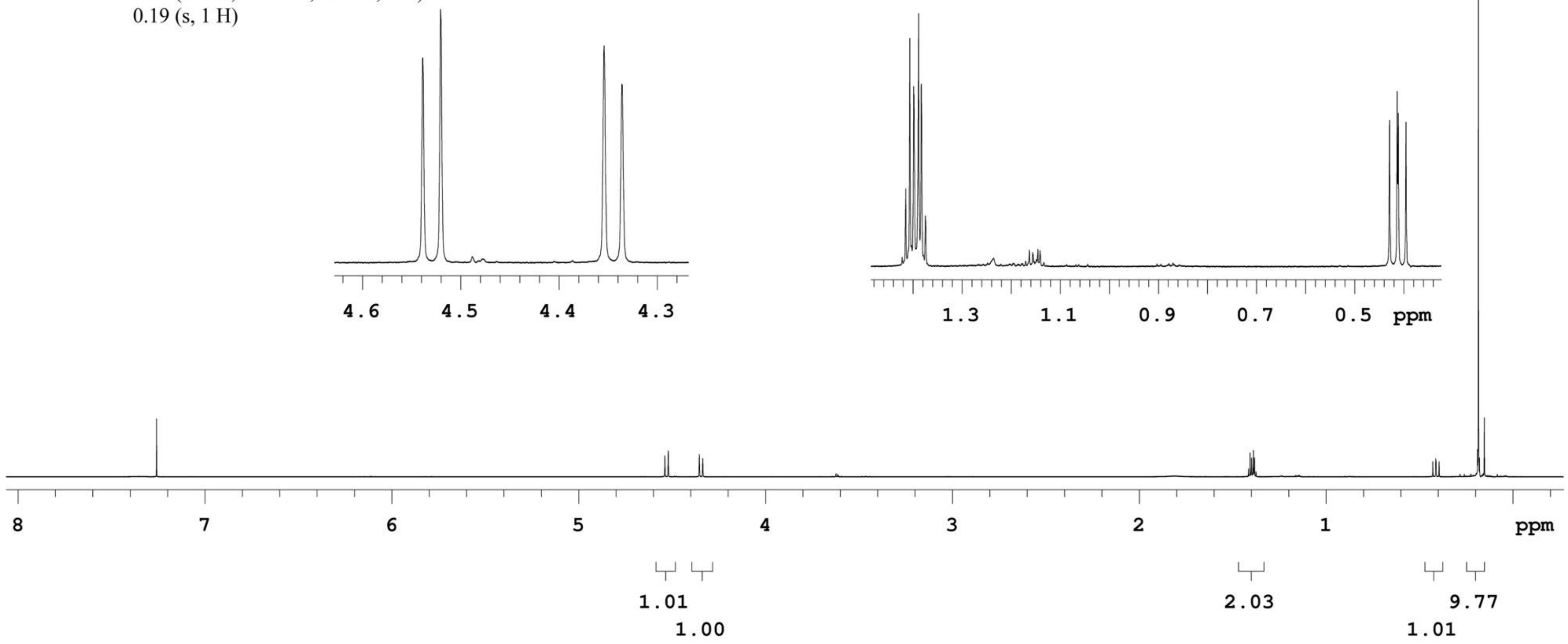

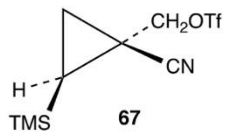

67

^{13}C NMR of **67** (150 MHz, $CDCl_3$)

119.3
118.5 (q, J = 320 Hz)
80.8
18.0
14.4
13.9
-2.3

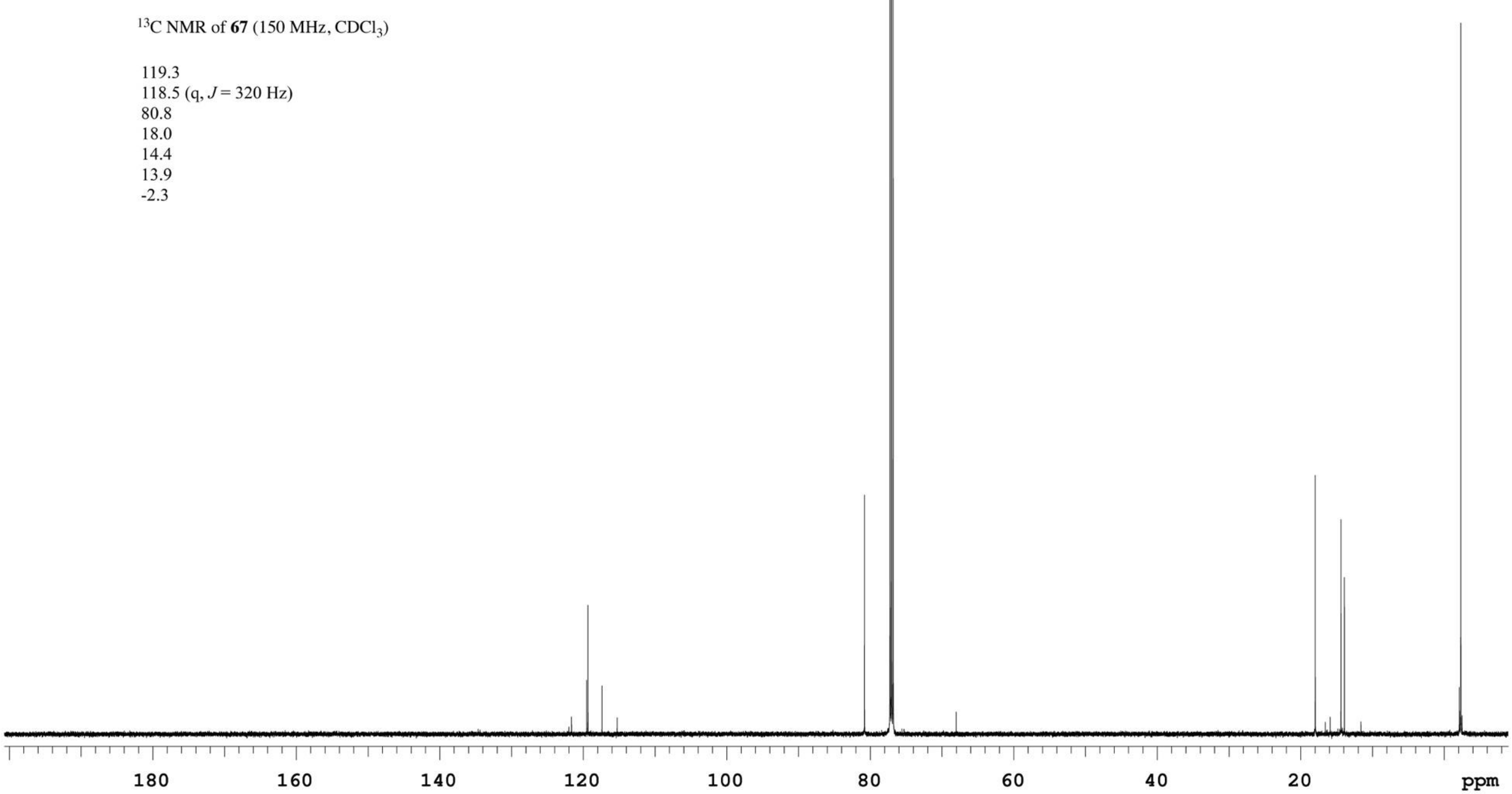

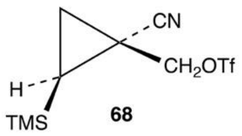

68

^{1}H NMR of **68** (600 MHz, $CDCl_3$)

4.49 (d, J = 11.0 Hz, 1 H)
4.44 (d, J = 11.0 Hz, 1 H)
1.78 (d of d of d, J = 11.0
4.9, 0.6 Hz, 1 H)
1.09 (d of d, J = 9.6, 4.9 Hz, 1 H)
0.82 (d of d, J = 11.0, 9.6 Hz, 1 H)
0.15 (s, 9 H)

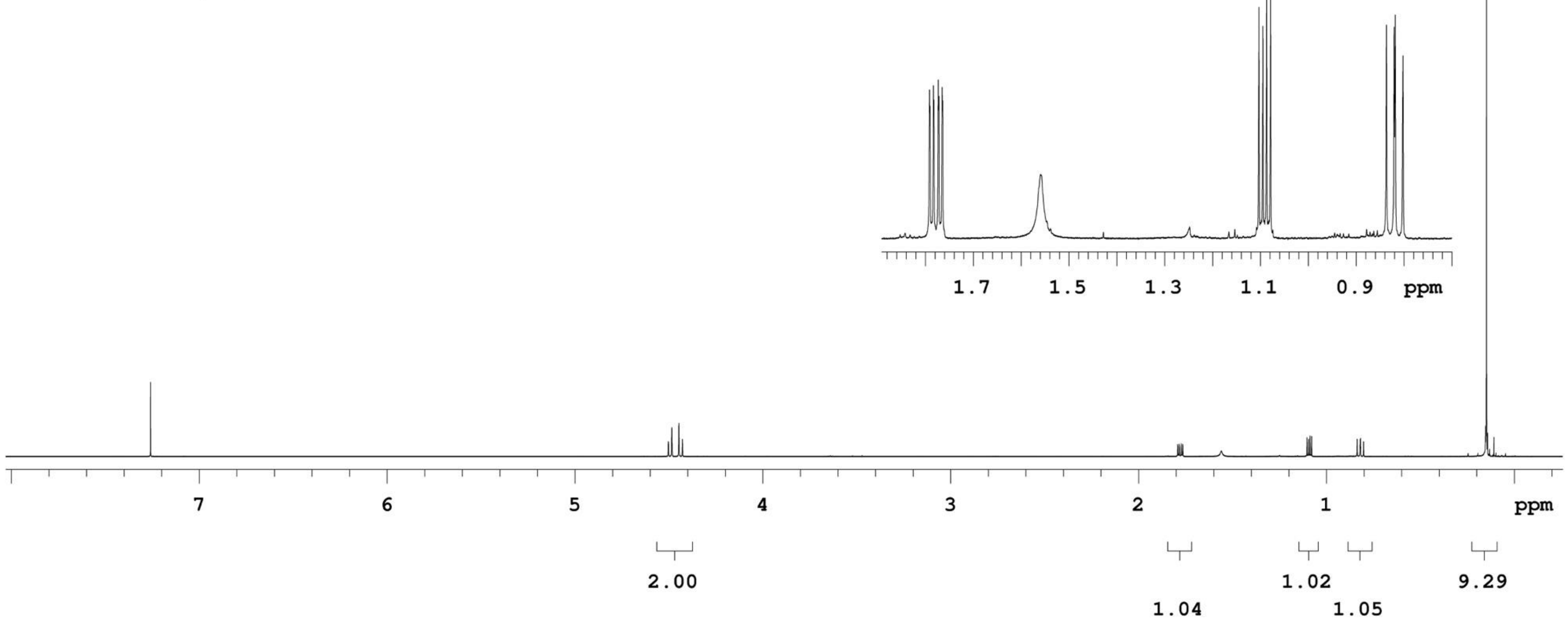

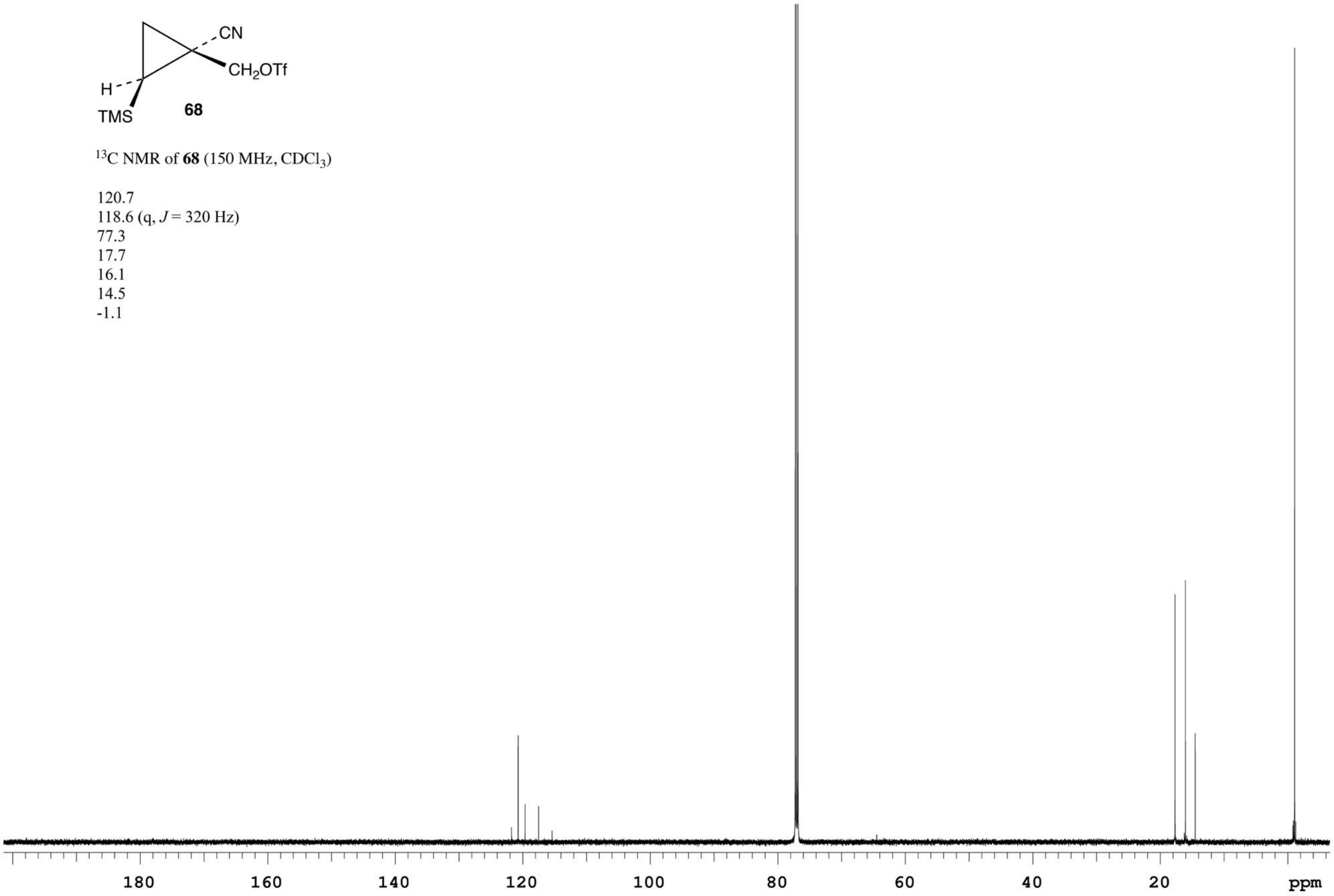

^{13}C NMR of **68** (150 MHz, $CDCl_3$)

120.7
118.6 (q, J = 320 Hz)
77.3
17.7
16.1
14.5
-1.1

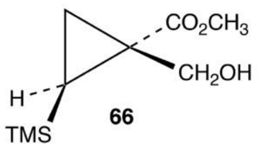

66

^{1}H NMR of **66** (600 MHz, $CDCl_3$)

3.72 (d of d, J = 12.2, 7.0 Hz, 1 H)
3.70 (s, 3 H)
3.64 (d of d, J = 12.2, 6.9 Hz, 1 H)
2.59 (t, J = 6.9 Hz, 1 H)
1.50 (d of d of d, J = 10.9, 3.4, 0.8 Hz, 1 H)
0.82 (d of d, J = 8.9, 3.4 Hz, 1 H)
0.62 (d of d, J = 10.8, 8.9 Hz, 1 H)
0.09 (s, 9 H)

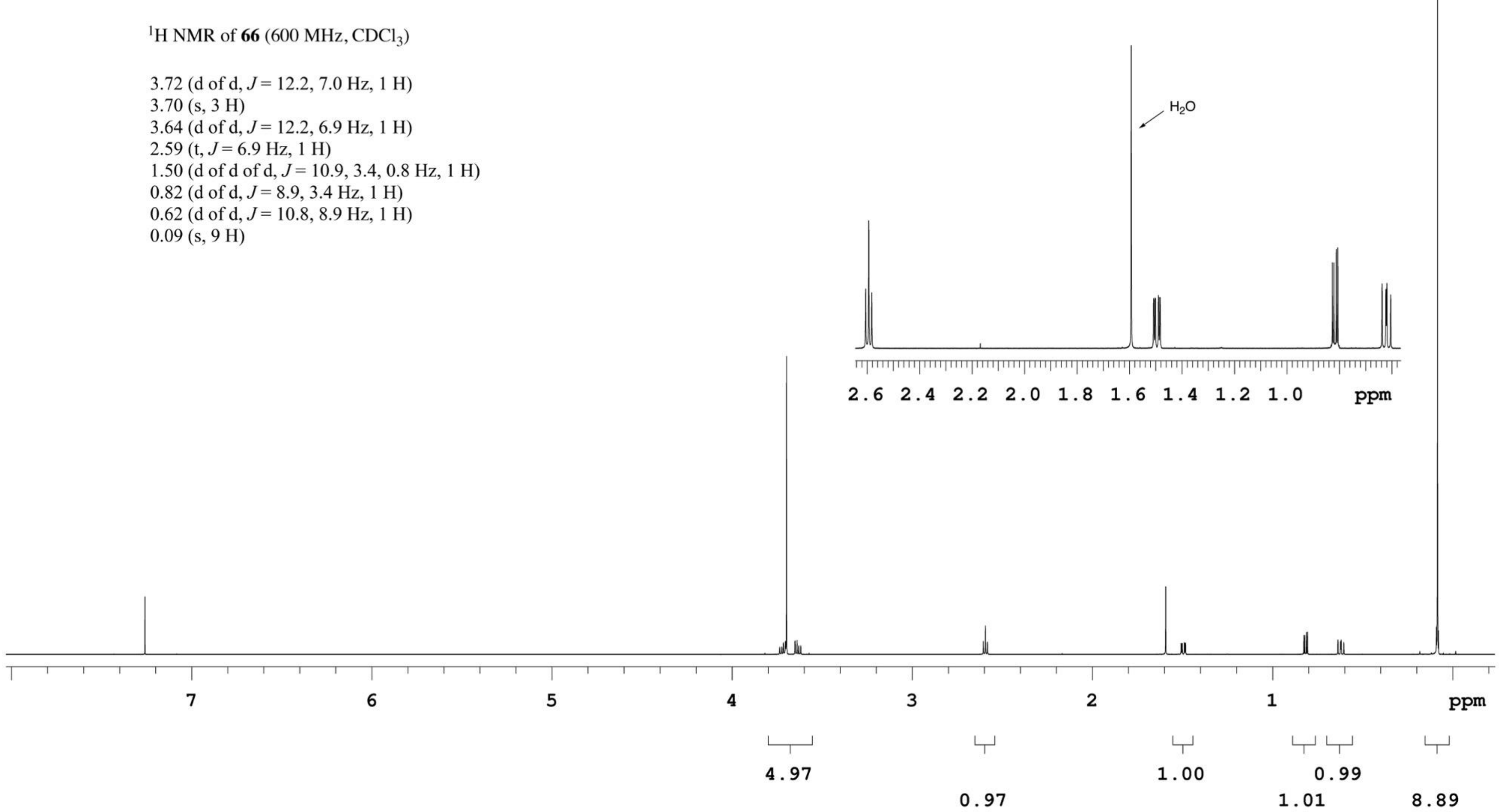

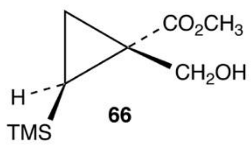

66

^{13}C NMR of **66** (150 MHz, $CDCl_3$)

176.1
64.2
52.1
30.5
18.0
15.4
-0.7

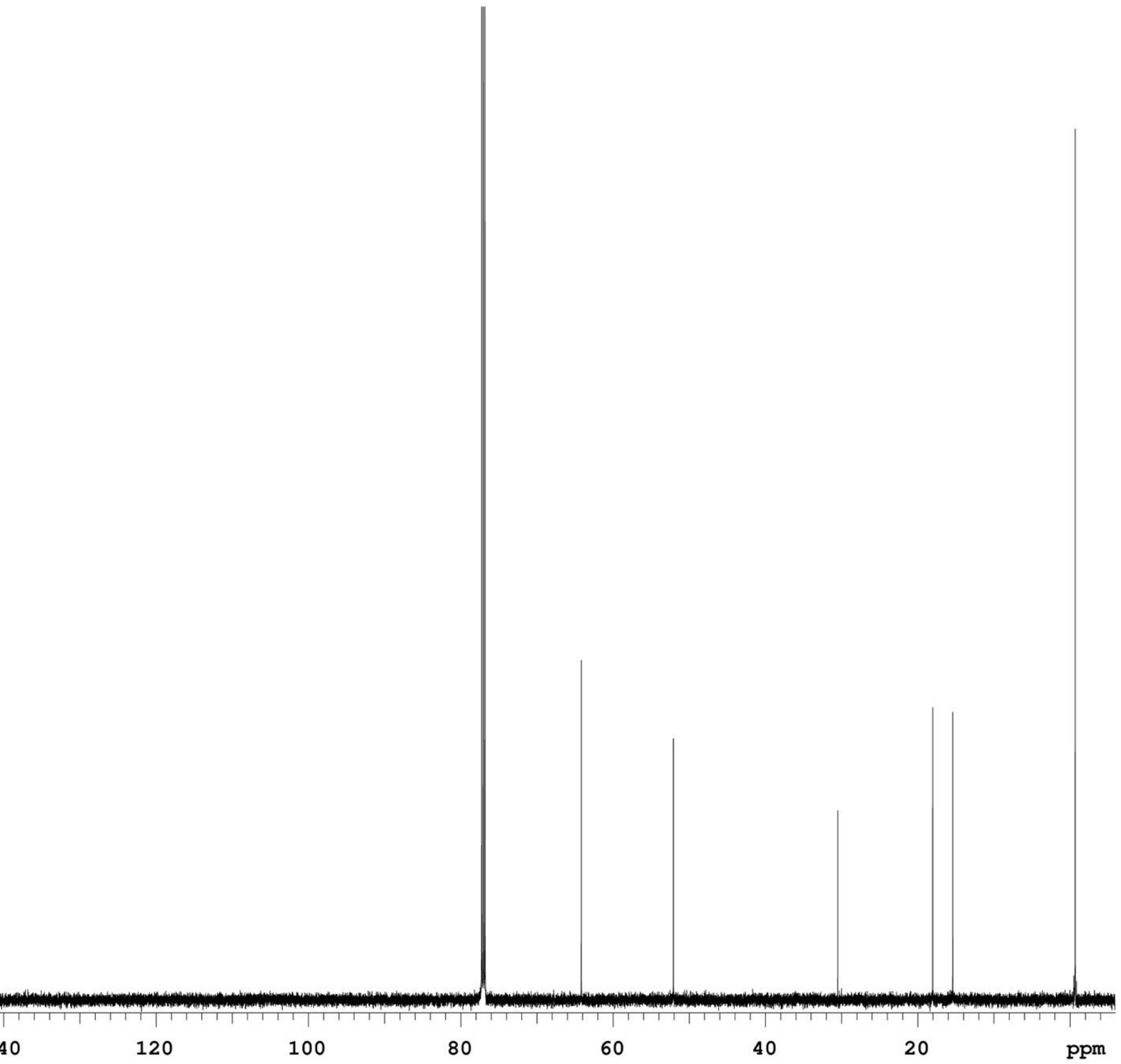

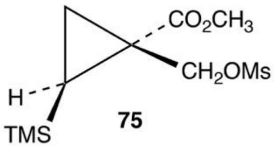

75

^{1}H NMR of **75** (600 MHz, $CDCl_3$)

4.40 (d, J = 10.6 Hz, 1 H)
4.33 (d, J = 10.6 Hz, 1 H)
3.70 (s, 3 H)
3.08 (s, 3 H)
1.63 (d of d of d, J = 10.9, 3.7, 0.4 Hz, 1 H)
0.95 (d of d, J = 9.2, 3.7 Hz, 1 H)
0.78 (d of d, J = 11.0, 9.2 Hz, 1 H)
0.11 (s, 9 H)

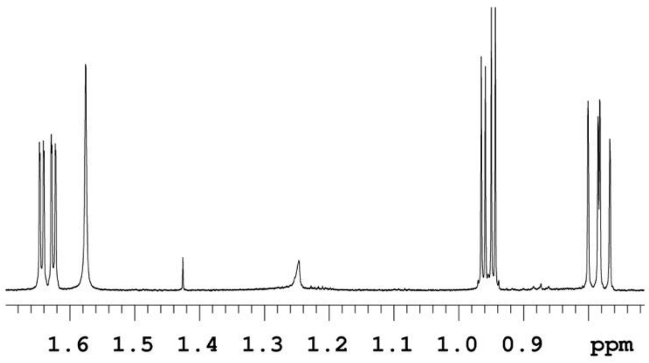

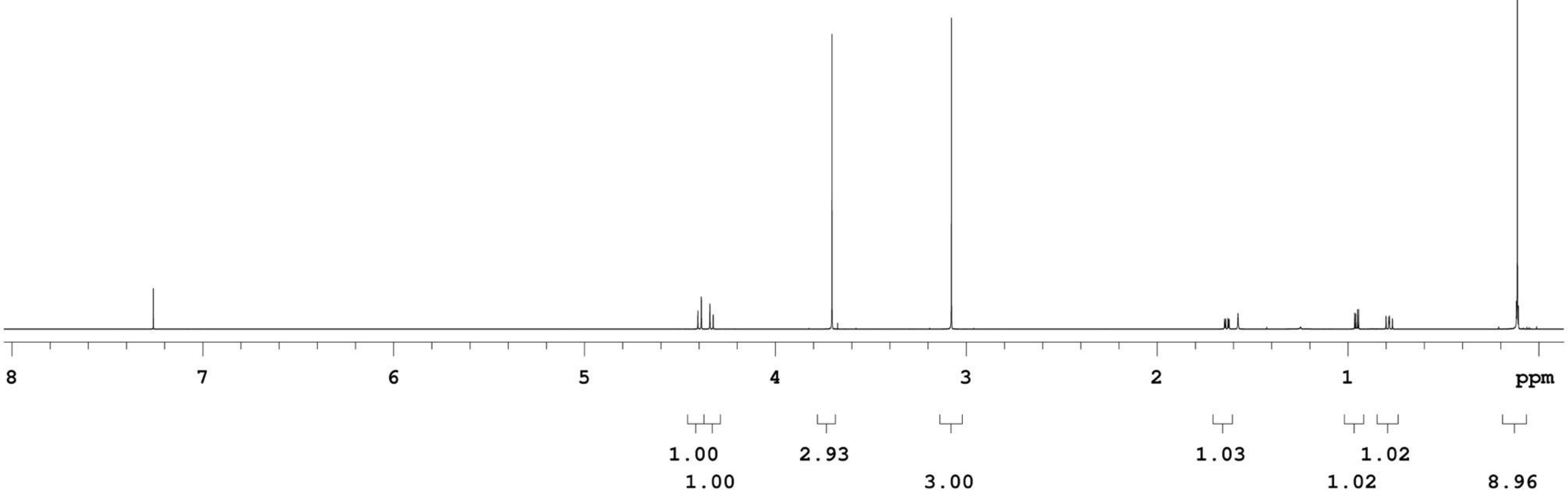

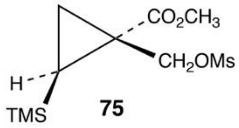

75

^{13}C NMR of **75** (150 MHz, $CDCl_3$)

173.6
71.6
52.4
37.3
27.5
18.5
16.3
-0.9

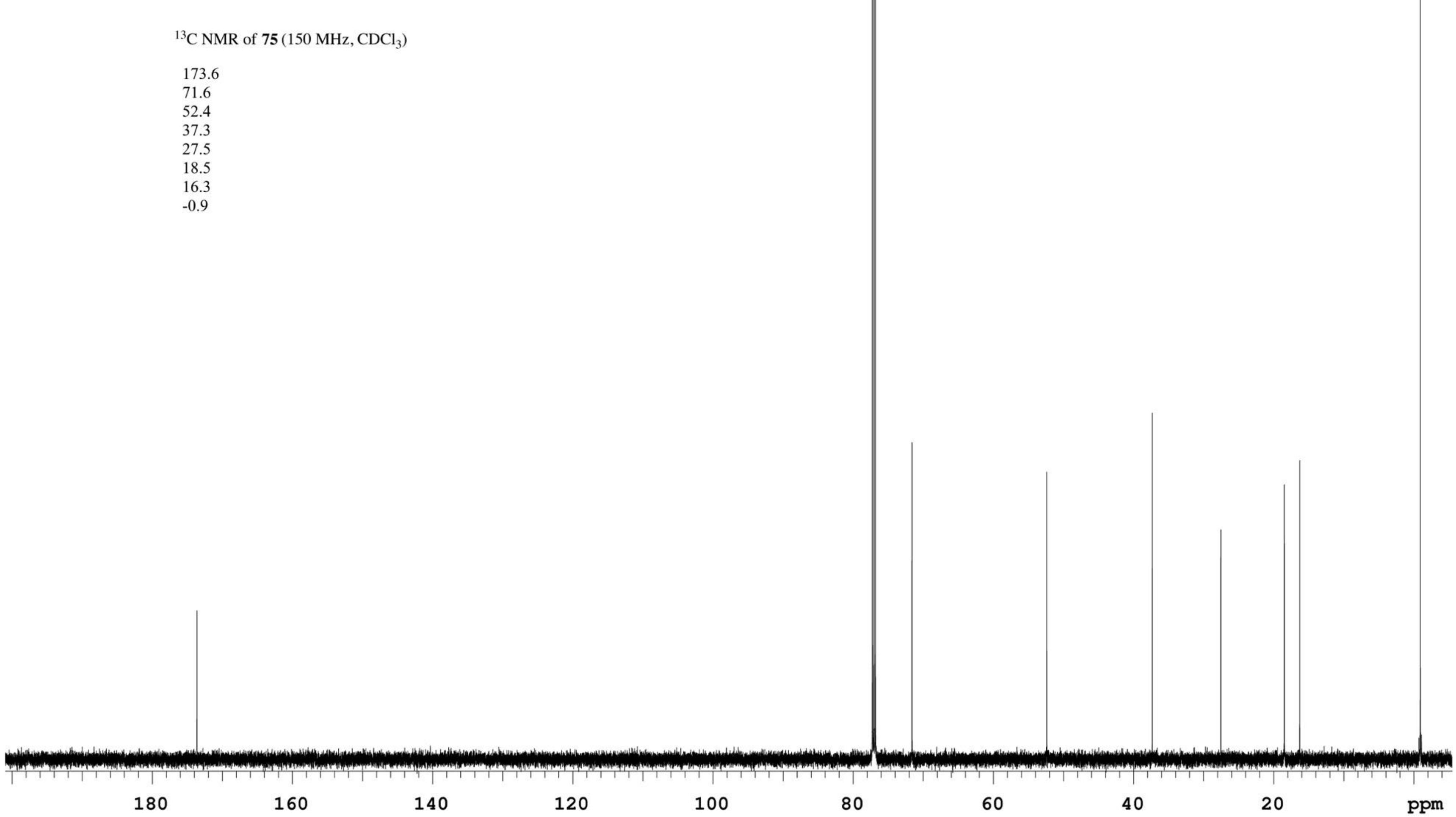

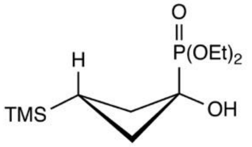

^1H NMR (600 MHz, $CDCl_3$)

4.19 (quintet, J = 7.2 Hz, 4 H)
3.32 (br (1 H)
2.55 (m, 2 H)
2.00 (m, 2 H)
1.51 (quintet, J = 10.2 Hz, 1 H)
1.34 (t, J = 7.1 Hz, 6 H)
-0.04 (s, 9 H

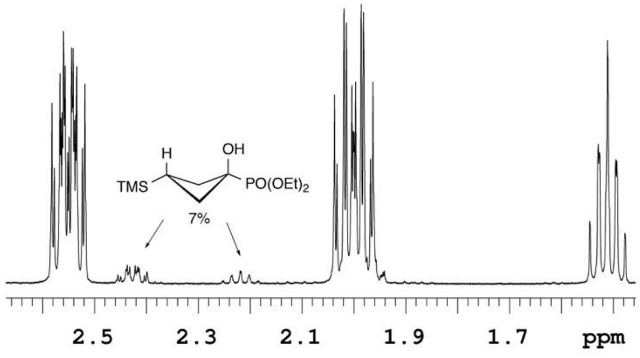

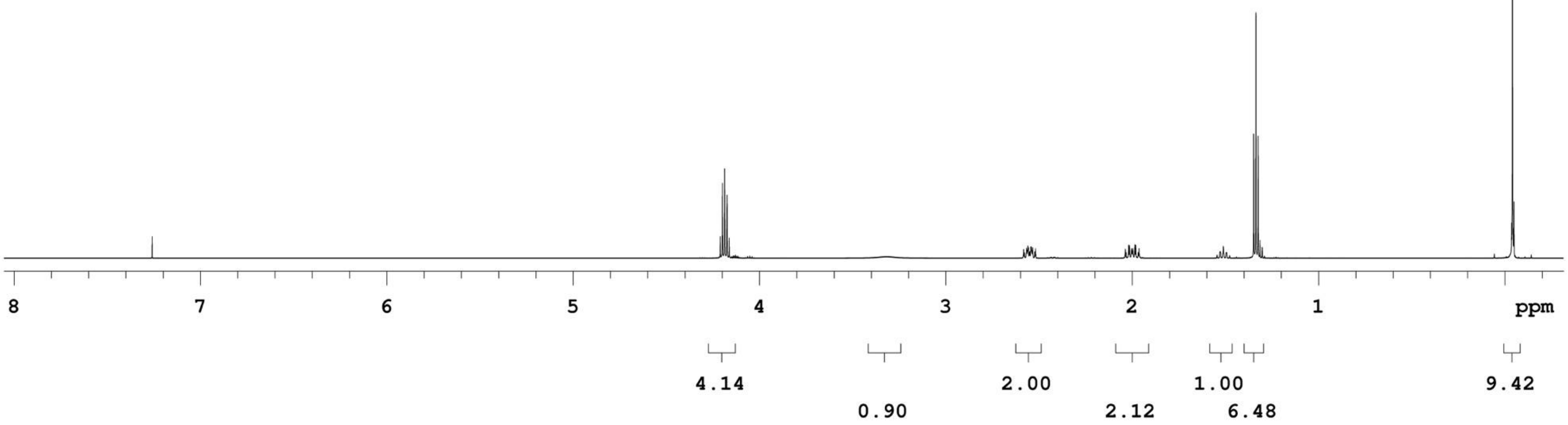

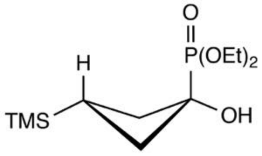

^{13}C NMR (150 MHz, $CDCl_3$)

71.1 (d, J = 156 Hz)
62.8 (d, J = 7.2 Hz)
33.7 (d, J = 1.2 Hz)
16.5 (d, J = 5.5 Hz)
12.5 (d, J = 3.4 Hz)
-3.6

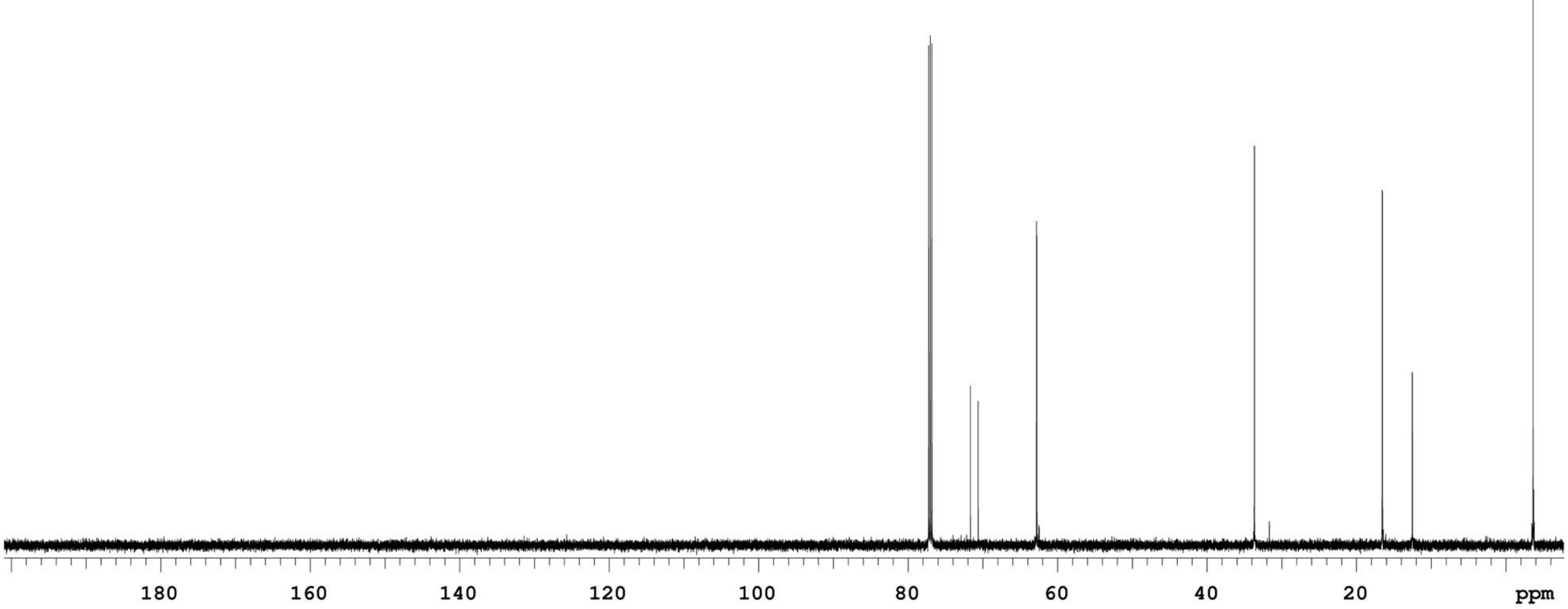

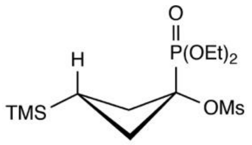

76

^{1}H NMR of **76** (600 MHz, $CDCl_3$)

4.26 (m, 4 H)
3.09 (s, 3 H)
2.80-2.65 (m, 4 H)
1.72 (quintet of m, J = 10.2 Hz, 1 H)
1.38 (t, J = 7.2 Hz, 6 H)
0.00 (s, 9 H)

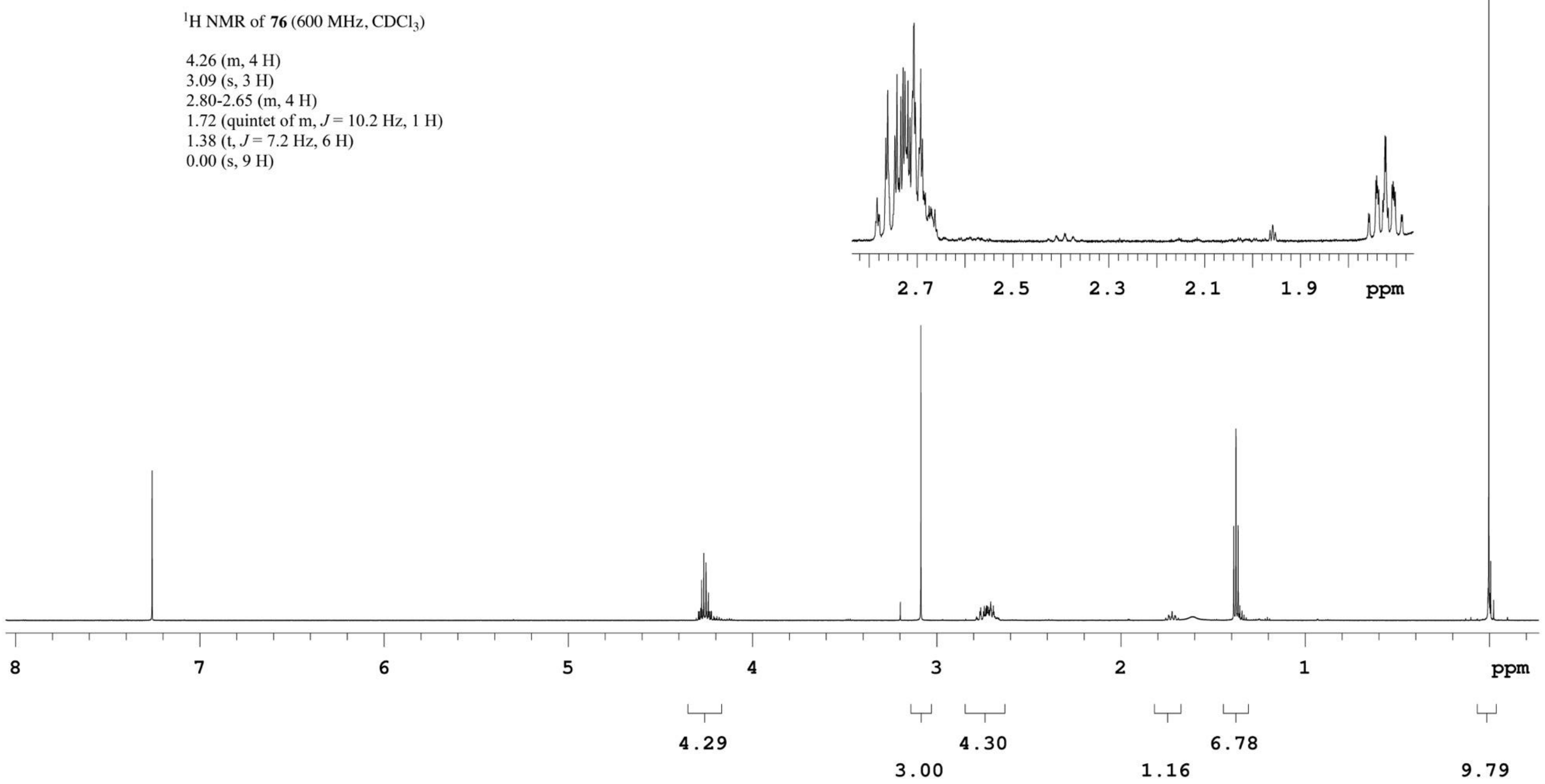

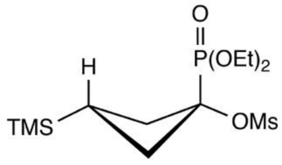

76

^{13}C NMR of **76** (150 MHz, $CDCl_3$)

82.6 (d, J = 164 Hz)
63.6 (d, J = 7.0 Hz)
40.4
33.6
16.5 (d, J = 5.7 Hz)
14.5 (d, J = 1.5 Hz)
-3.6

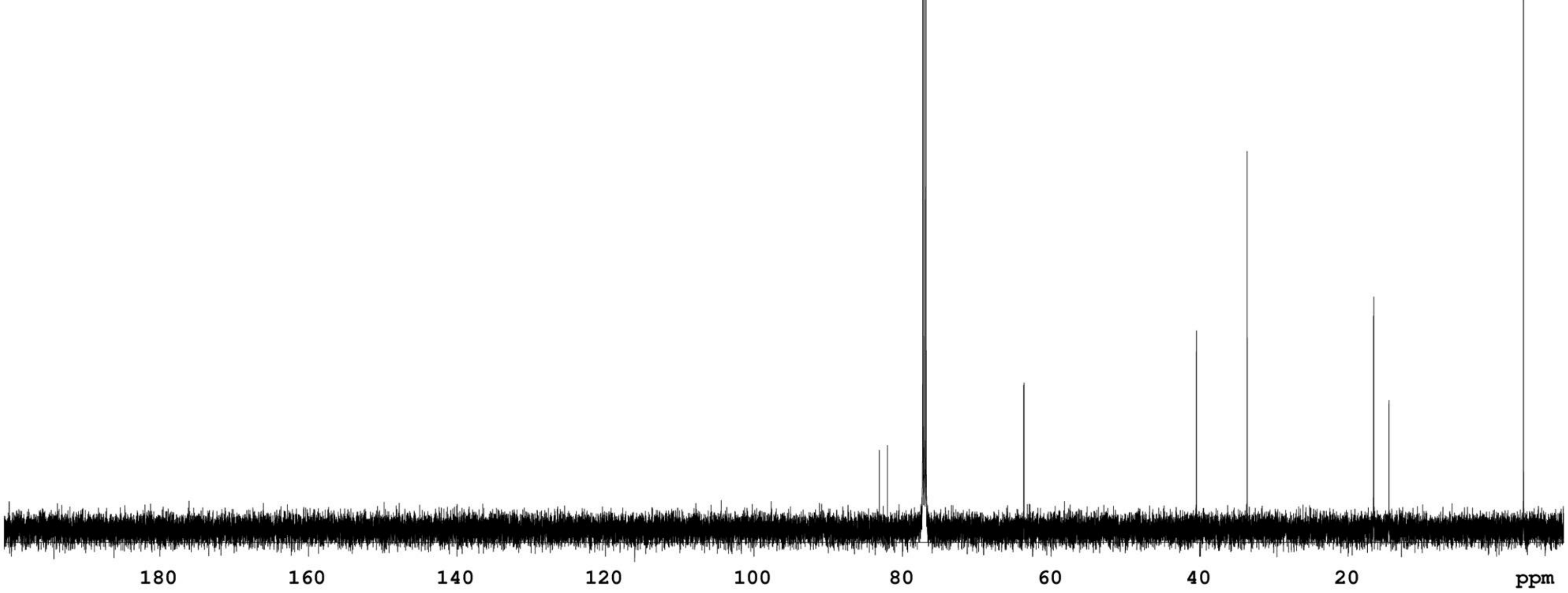

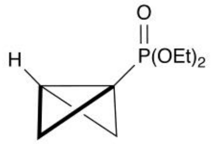

77

^{1}H NMR of **77** (600 MHz, $CDCl_3$)

4.12 (quintet, J = 7.3 Hz, 4 H)
2.13 (d of m, J = 22.8 Hz, 1 H)
1.95 (t, J = 3.3 Hz, 2 H)
1.33 (t, J = 7.2 Hz, 6 H)
0.93 (t, J = 2.3 Hz, 2 H)

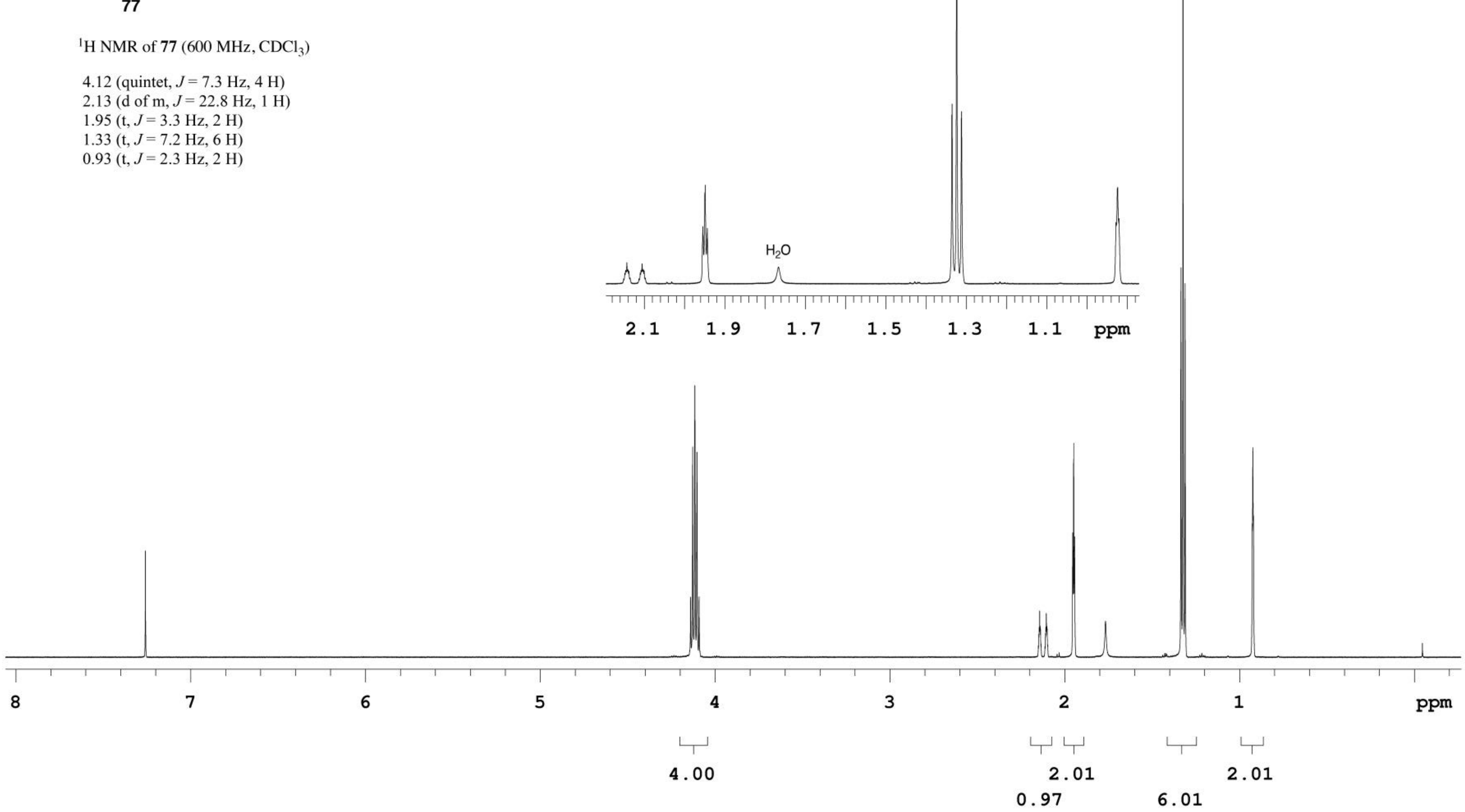

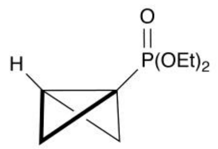

77

^{13}C NMR of **77** (150 MHz, $CDCl_3$)

62.0 (d, J = 6.0 Hz)
34.5 (d, J = 5.7 Hz)
16.4 (d, J = 6.4 Hz)
7.9 (d, J = 8.0 Hz)
-2.6 (d, J = 233 Hz)

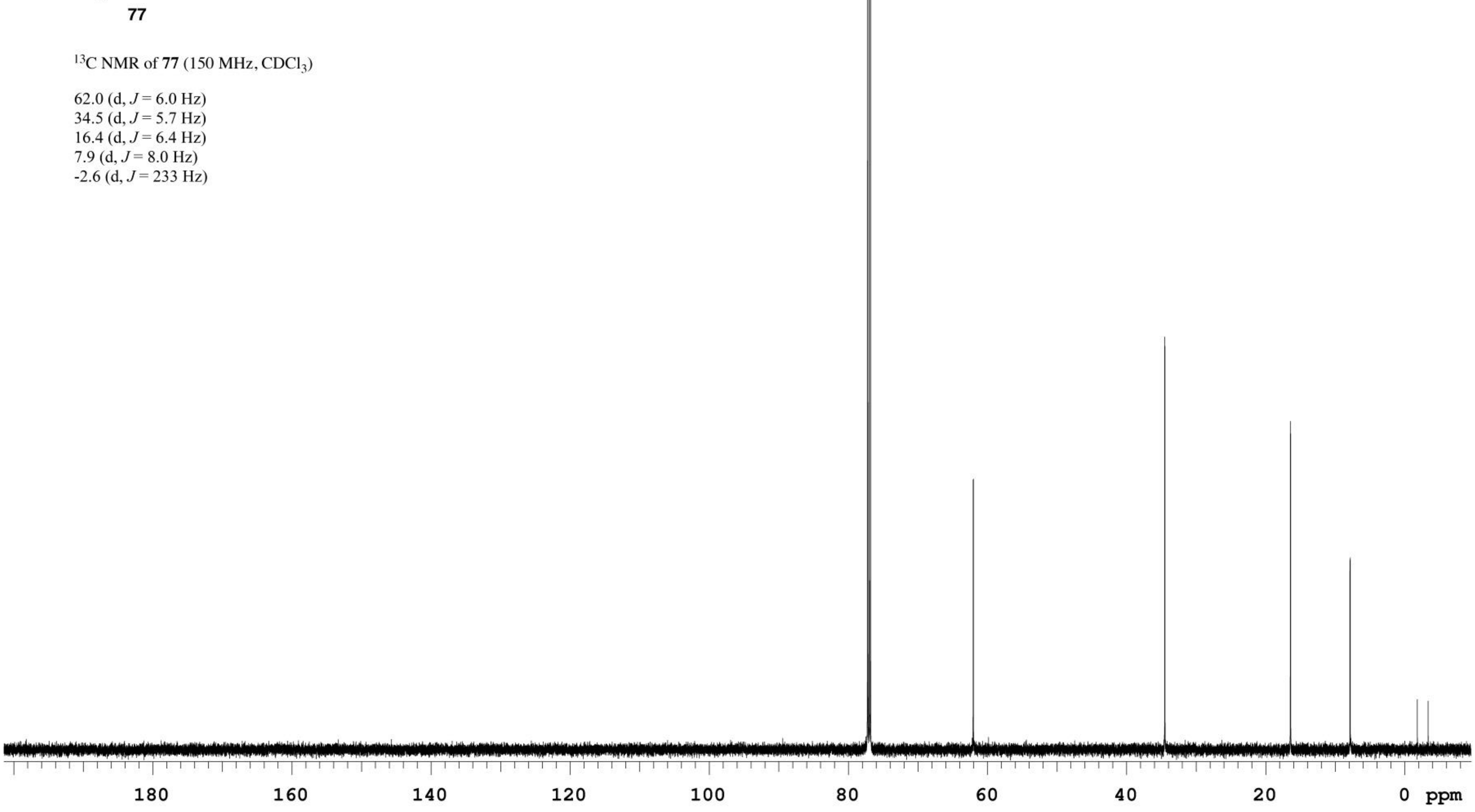

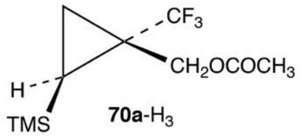

70a-H_3

^{1}H NMR of **70**-H_3 (600 MHz, $CDCl_3$)

4.46 (d, J = 12.8 Hz, 1 H)
3.93 (d, J = 12.8 Hz, 1 H)
2.08 (s, 3 H)
1.28 (d of d, J = 11.1, 4.6 Hz, 1 H)
0.70 (m, 1 H)
0.35 (d of d, J = 11.1, 8.8 Hz, 1 H)
0.09 (s, 9 H)

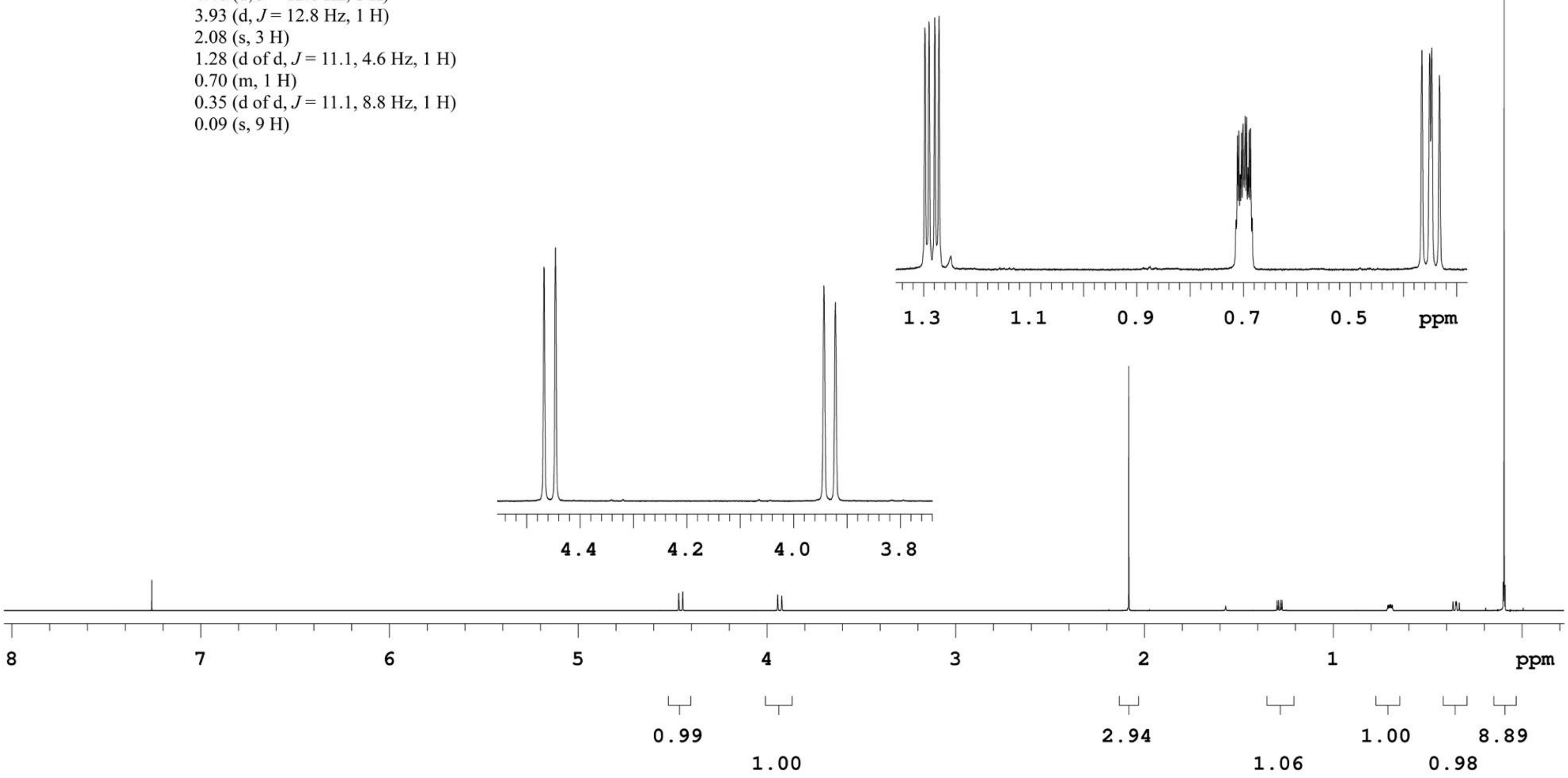

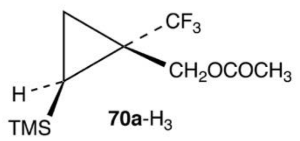

70a-H_3

^{13}C NMR of **70**-H_3 (150 MHz, $CDCl_3$)

170.7
126.5 (q, J = 275 Hz)
63.7 (q, J = 0.9 Hz)
26.9 (q, J = 32.5 Hz)
20.9
12.0 (q, J = 2.4 Hz)
8.0
-1.0

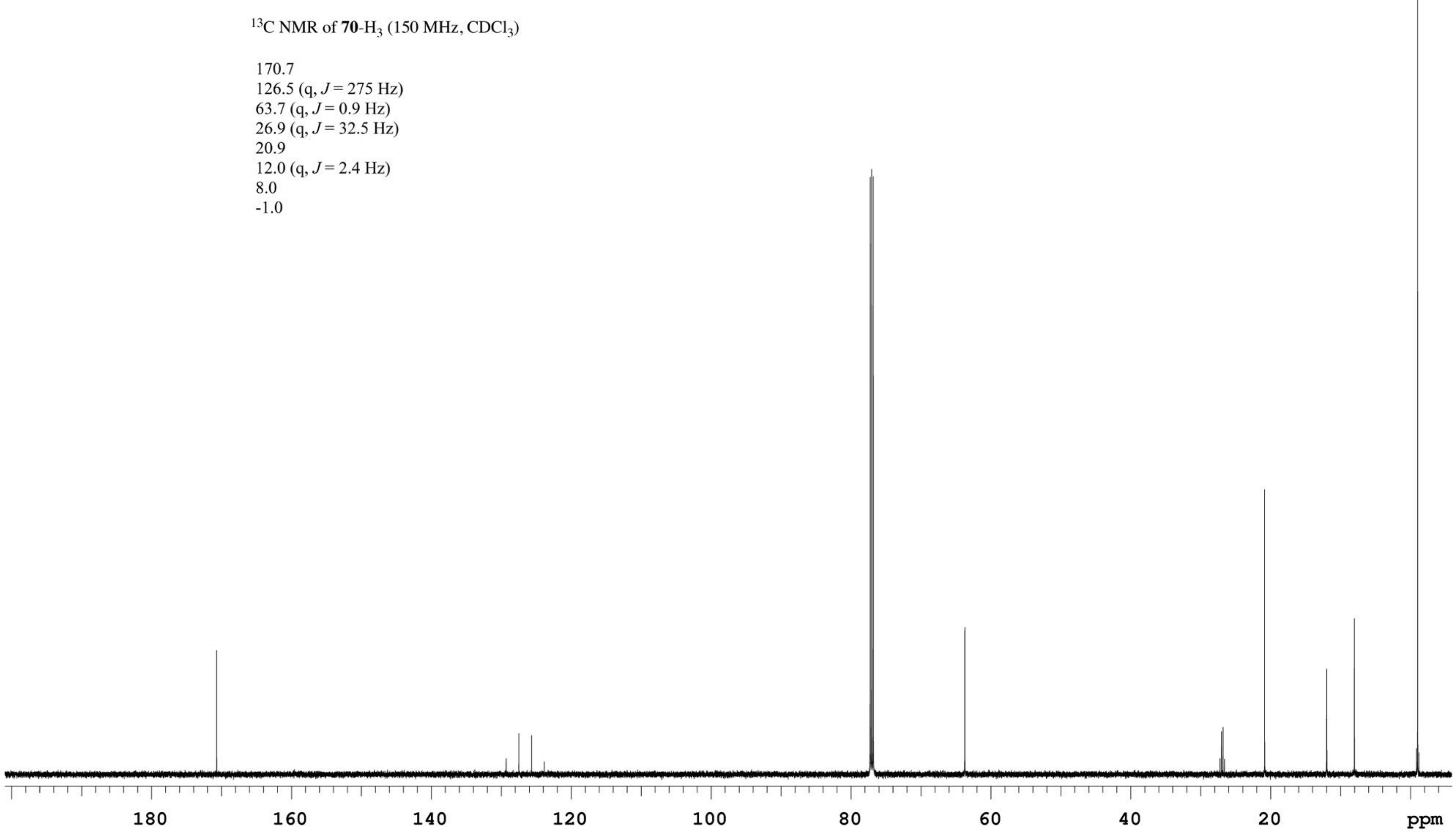

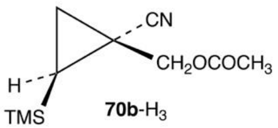

^{1}H NMR of **70b**-H_3 (600 MHz, $CDCl_3$)

4.17 (d, J = 12.1 Hz, 1 H)
3.93 (d, J = 12.1 Hz, 1 H)
2.13 (s, 3 H)
1.60 (d of d of d, J = 11.1, 4.6, 0.7 Hz, 1 H)
0.94 (d of d, J = 9.3, 4.6 Hz, 1 H)
0.62 (d of d, J = 11.1, 9.3 Hz, 1 H)
0.10 (s, 9 H)

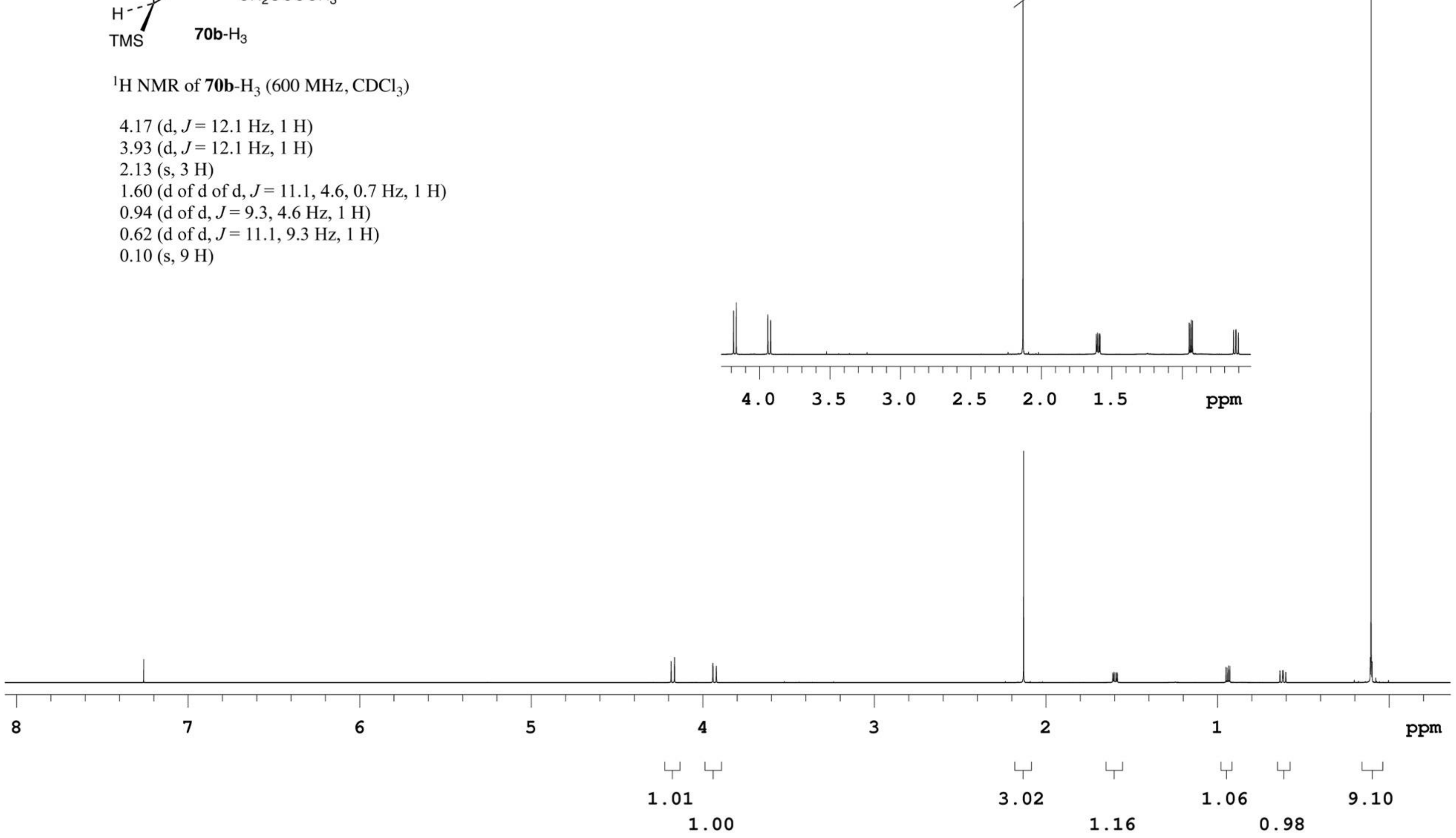

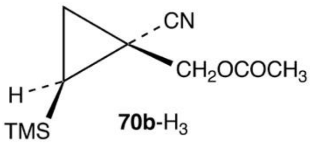

70b-H_3

^{13}C NMR of **70b**-H_3 (150 MHz, $CDCl_3$)

170.5
122.7
65.3
20.7
16.8
14.3
14.0
-1.0

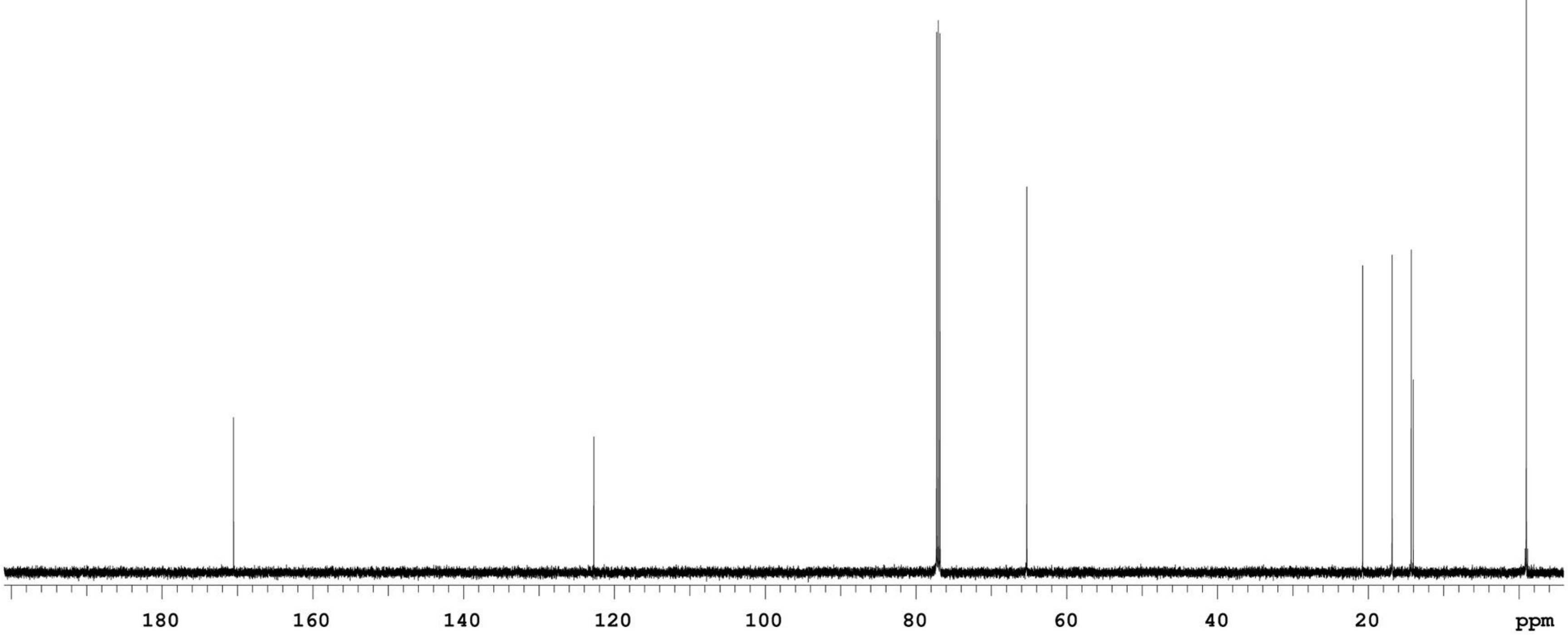

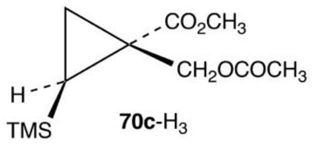

70c-H_3

^{1}H NMR of **70c**-H_3 (600 MHz, $CDCl_3$)

4.38 (d, J = 11.9 Hz, 1 H)
4.10 (d, J = 11.9 Hz, 1 H)
3.69 (s, 1 H)
2.07 (s, 3 H)
1.54 (d of d, J = 10.8, 3.4 Hz, 1 H)
0.84 (d of d, J = 9.0, 3.4 Hz, 1 H)
0.70 (d of d, J = 10.9, 9.0 Hz, 1 H)
0.08 (s, 9 H)

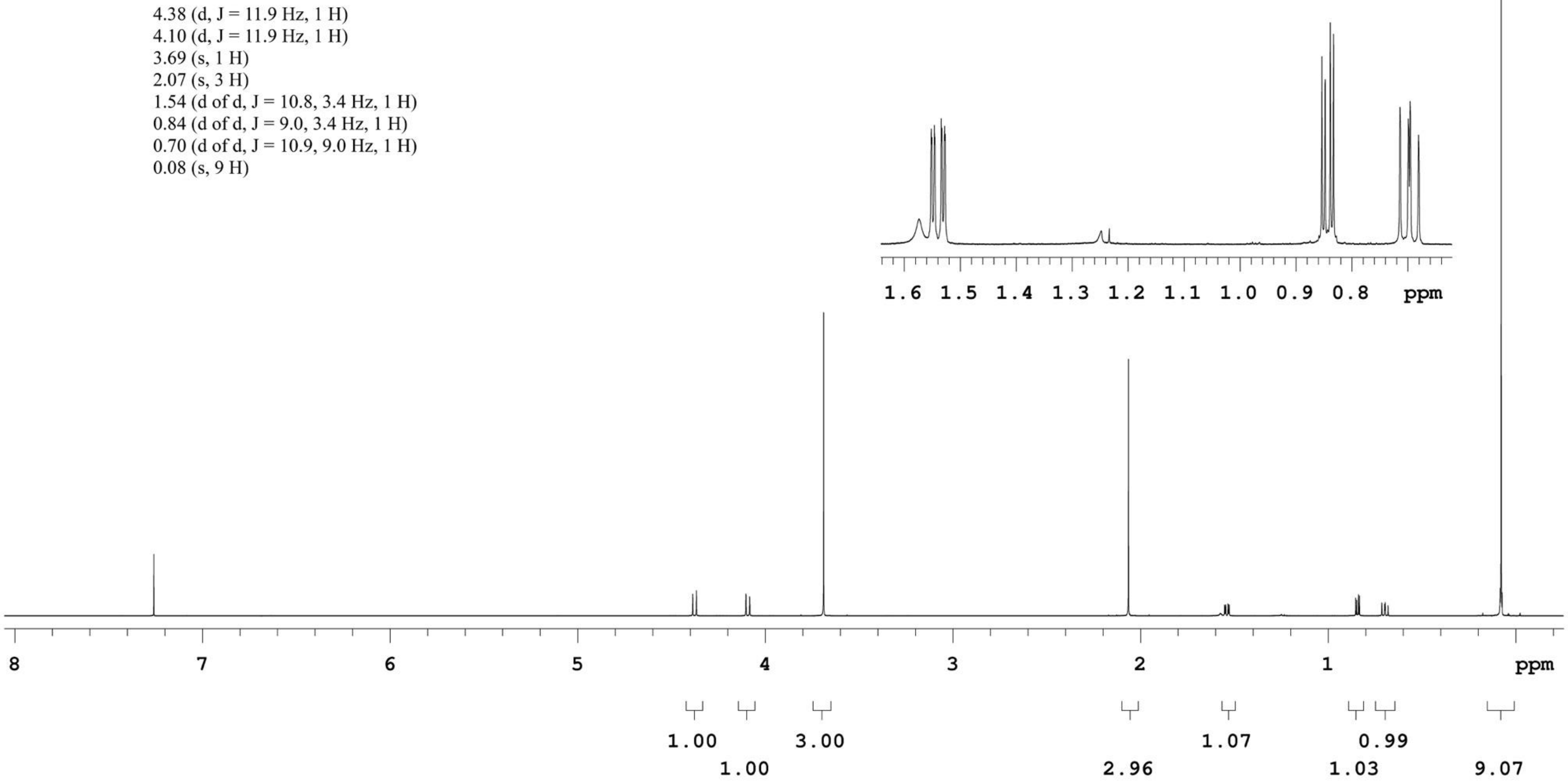

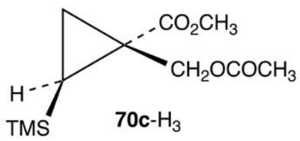

70c-H_3

^{13}C NMR of **70c**-H_3 (150 MHz, $CDCl_3$)

174.5
171.0
65.5
52.3
27.4
21.0
18.2
15.6
-0.8

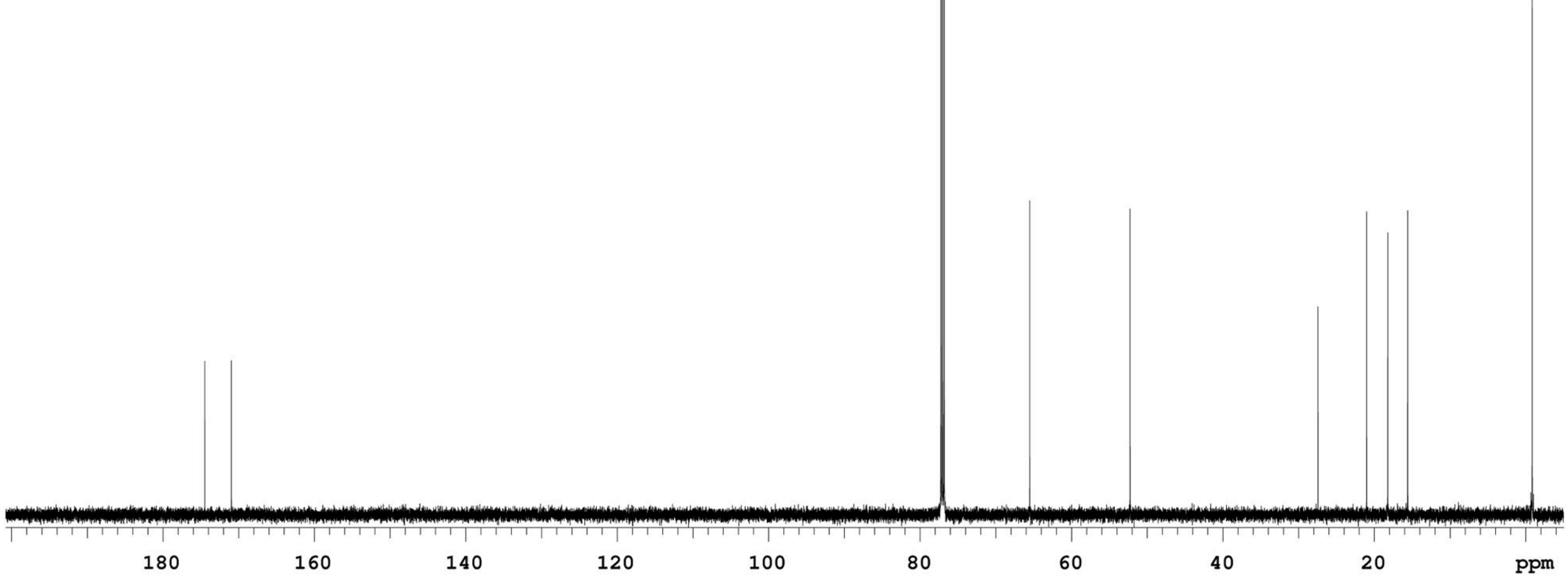

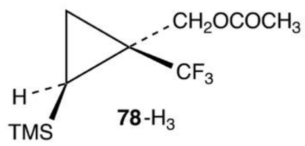

78-H_3

^{1}H NMR of **78**-H_3 (600 MHz, $CDCl_3$)

4.27 (d, J = 12.3 Hz, 1 H)
4.03 (d, J = 12.3 Hz, 1 H)
2.08 (s, 3 H)
1.05-0.98 (m, 2 H)
0.11 (m, 1 H)
0.07 (q, J = 0.8 Hz, 9 H)

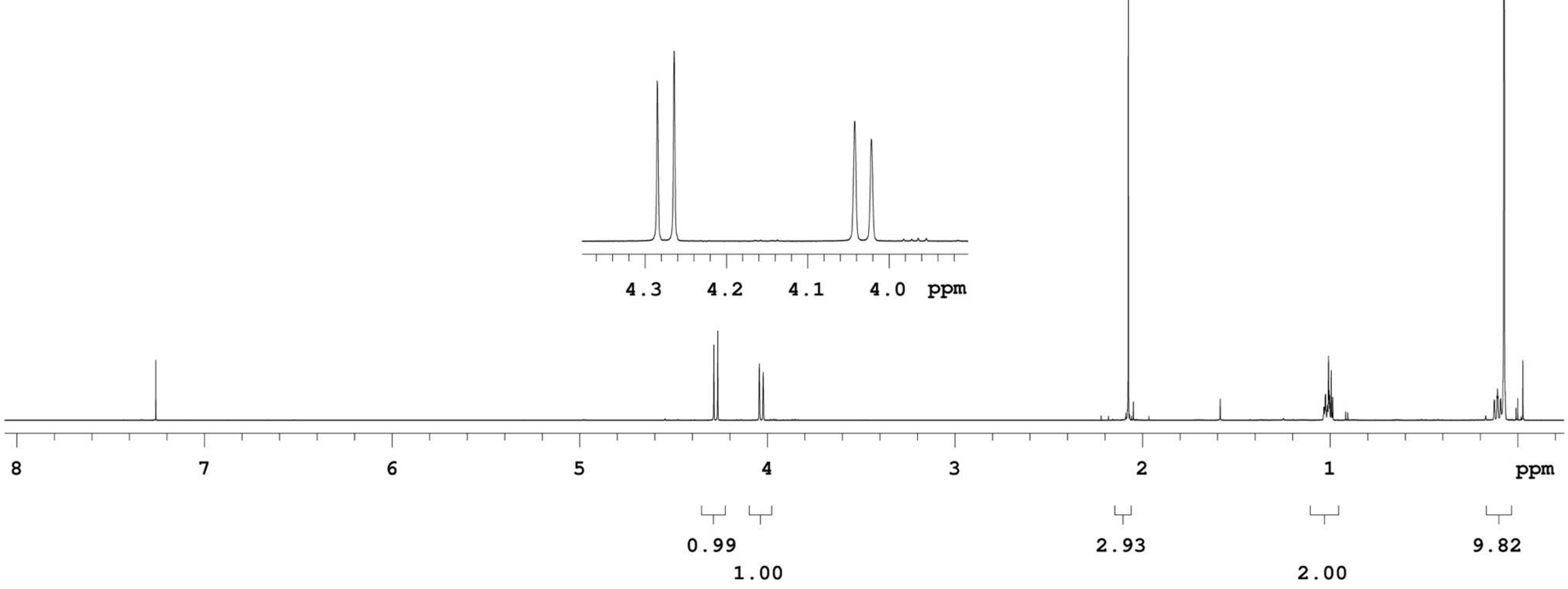

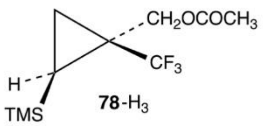

78-H_3

^{13}C NMR of **78**-H_3 (150 MHz, $CDCl_3$)

170.9
127.1 (q, J = 175 Hz)
67.1 (q, J = 1.7 Hz)
27.4 (q, J = 33 Hz)
20.9
12.5 (q, J = 2.2 Hz)
10.8
-1.1 (q, J = 2.0 Hz)

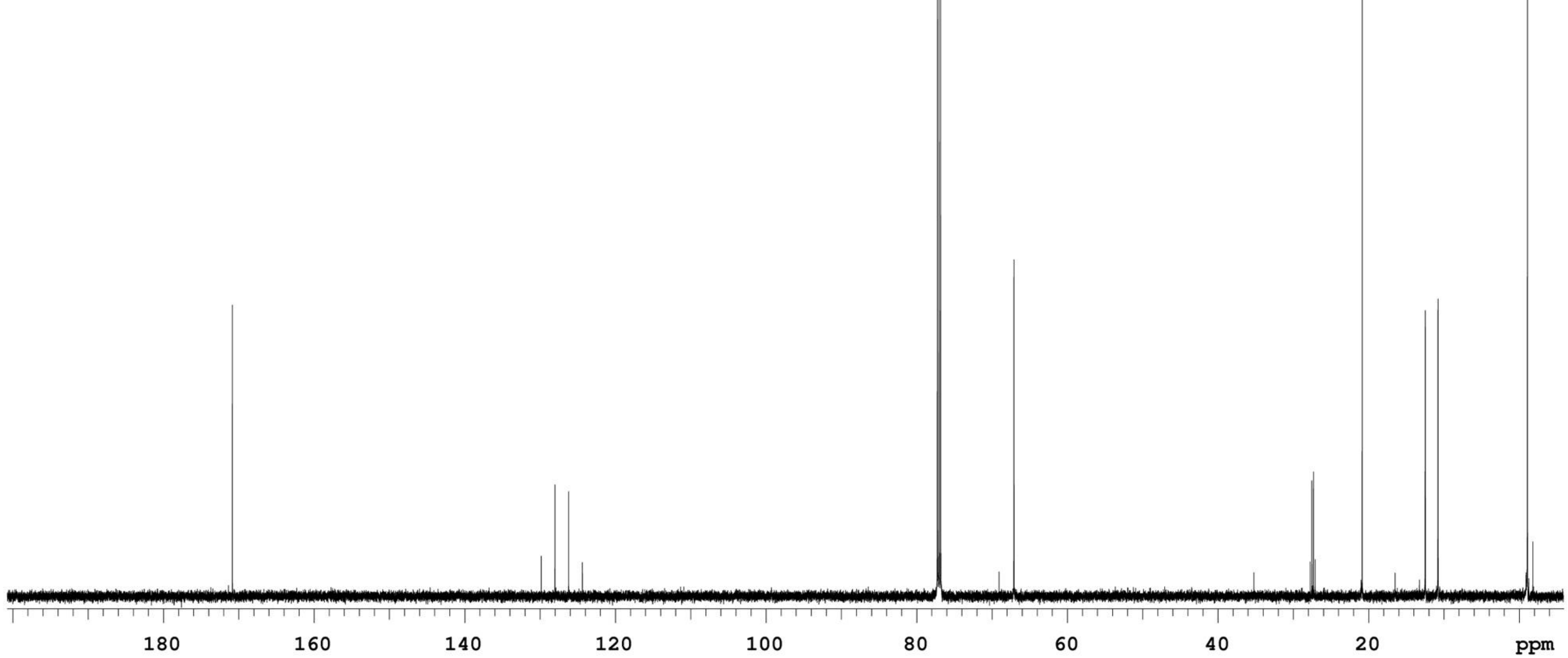

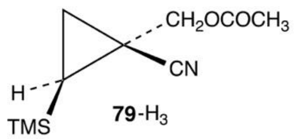

79-H_3

^{1}H NMR of **79**-H_3 (600 MHz, $CDCl_3$)

4.07 (d, J = 11.7 Hz, 1 H)
4.01 (d, J = 11.7 Hz, 1 H)
2.12 (s, 3 H)
1.23 (d of d, J = 10.6, 4.7 Hz, 1 H)
1.19 (d of d, J = 9.0, 4.6 Hz, 1 H)
0.25 (d of d, J = 10.6, 9.0 Hz, 1 H)
0.15 (s, 9 H)

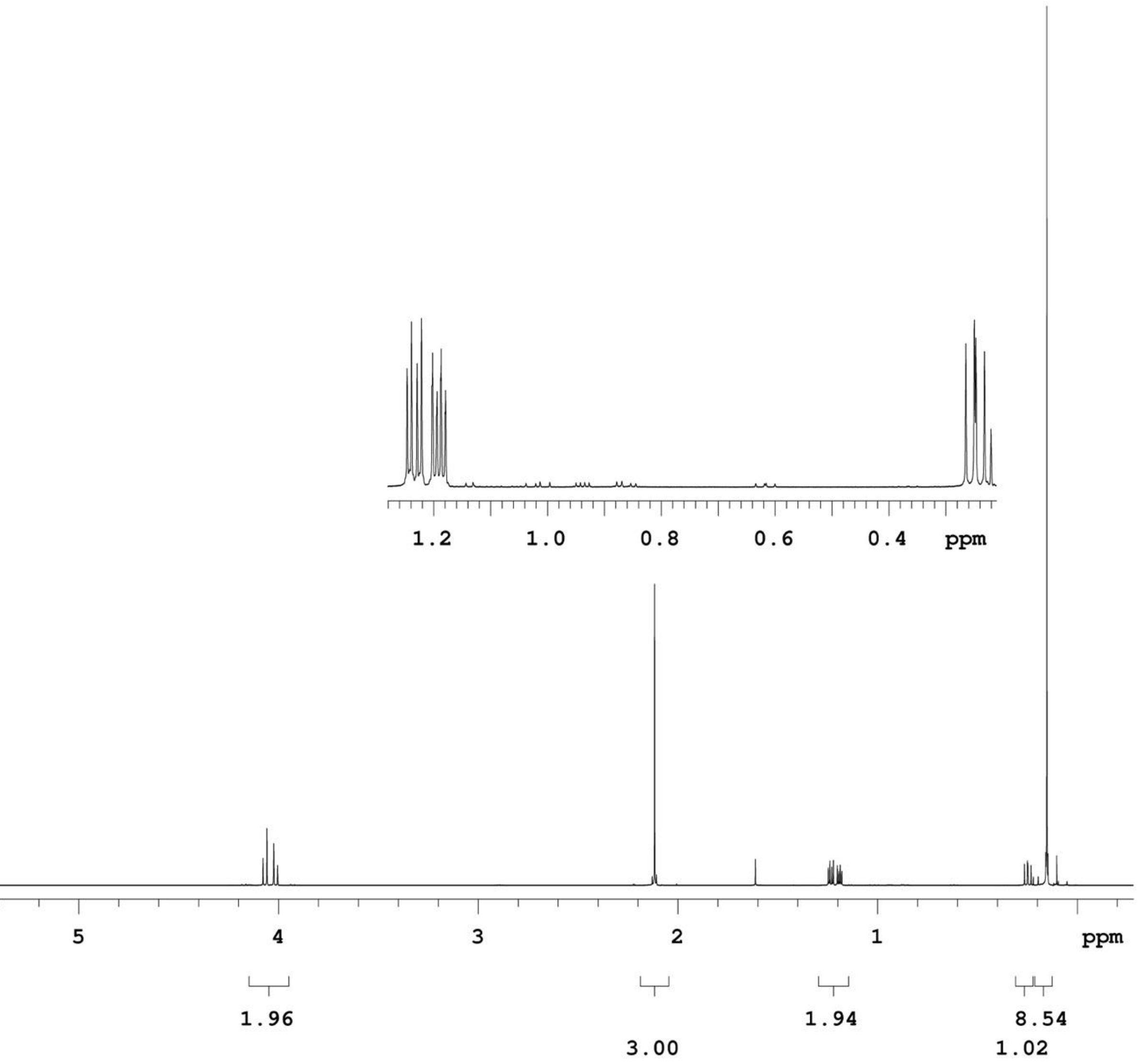

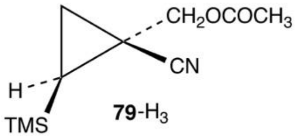

79-H_3

^{13}C NMR of **79**-H_3 (150 MHz, $CDCl_3$)

170.6
121.2
68.6
20.8
16.8
13.6
13.0
-2.2

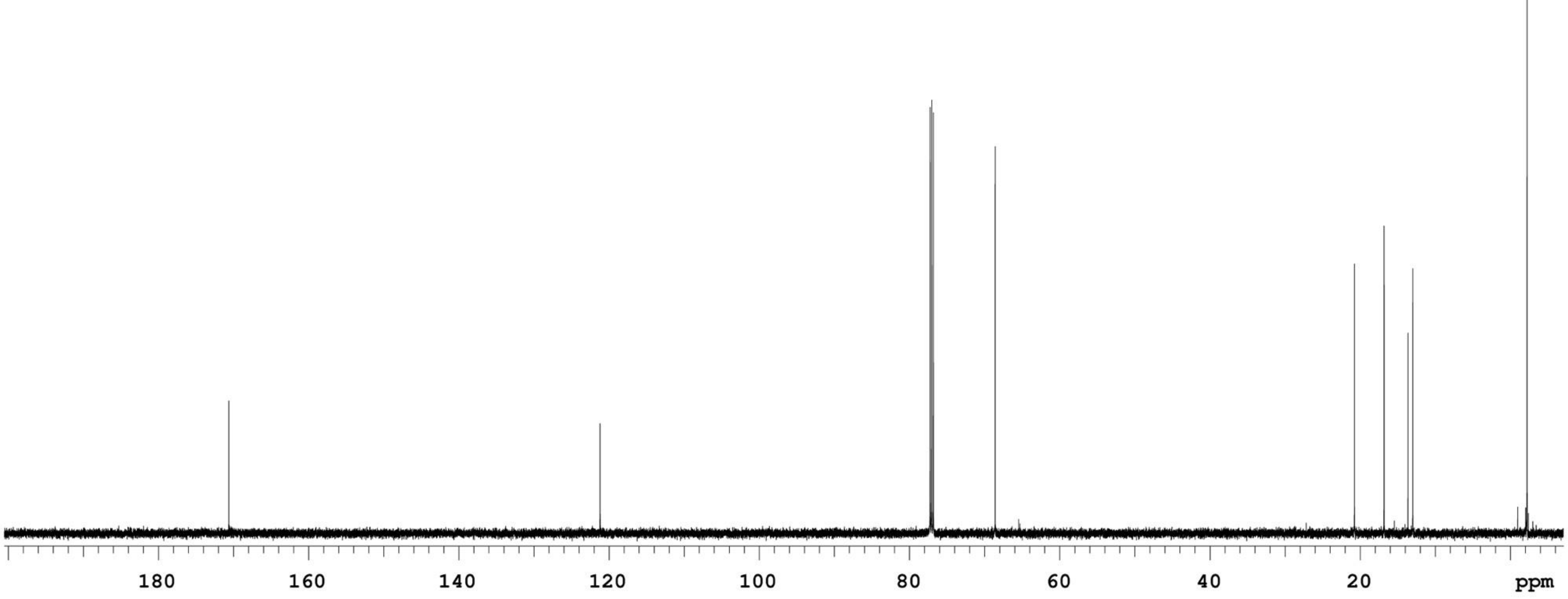

Supplement: File 1 — Experimental details and 1H and 13C NMR spectra of new compounds. [file Beilstein_J_Org_Chem-15-1769-s001.pdf]
