# Supplementary material for: The cyclopropylcarbinyl route to γ-silyl carbocations
Source: Beilstein J Org Chem. 2019 Jul 24;15:1769–80. doi: 10.3762/bjoc.15.170 (PMC6664402; doi:10.3762/bjoc.15.170)
Supplement: File 2 — M062X/6-611+G** calculated structures, energies, and Cartesian coordinates for carbocations and transition states. [file Beilstein_J_Org_Chem-15-1769-s002.pdf]

BEILSTEIN JOURNAL OF ORGANIC CHEMISTRY

Supporting Information

for

The cyclopropylcarbinyl route to γ-silyl carbocations

Xavier Creary

Beilstein J. Org. Chem. **2019,** *15,* 1769–1780. doi:10.3762/bjoc.15.170

M062X/6-611+G** calculated structures, energies, and Cartesian coordinates for carbocations and transition states

License and Terms: This is a supporting information file under the terms of the Creative Commons Attribution License (http://creativecommons.org/licenses/by/4.0). Please note that the reuse, redistribution and reproduction in particular requires that the authors and source are credited.
The license is subject to the *Beilstein Journal of Organic Chemistry* terms and conditions: (https://www.beilstein-journals.org/bjoc)

M062X/6-311+G** Calculated[1] Structures, Energies, and Cartesian Coordinates for Carbocations and Transition States, as well as B3LYP/6-31G* Calculated[2] Structures and NMR Spectra of **17** and **18**

1. Frisch, M, J.; Trucks, G. W.; Schlegel, H. B.; Scuseria, G. E.; Robb, M. A.; Cheeseman, J. R.; Scalmani, G.; Barone, V.; Mennucci, B.; Petersson, G. A.; Nakatsuji, H.; Caricato, M.; Li, X.; Hratchian, H. P.; Izmaylov, A. F.; Bloino, J.; Zheng, G.; Sonnenberg, J. L.; Hada, M.; Ehara, M; Toyota, K.; Fukuda, R.; Hasegawa, J.; Ishida, M.; Nakajima, T.; Honda, Y.; Kitao, O.; Nakai, H.; Vreven, T.; Montgomery, Jr., J. A.; Peralta, J. E.; Ogliaro, F.; Bearpark, M.; Heyd, J. J.; Brothers, E.; Kudin, K. N.; Staroverov,V. N.; Kobayashi, R.; Normand, J.; Raghavachari, K.; Rendell, A.; Burant, J. C.; Iyengar, S. S.; Tomasi, J.; Cossi, M.; Rega, N.; Millam, J. M.; Klene, M.; Knox, J. E.; Cross, J. B.; Bakken, V.; Adamo, C.; Jaramillo, J.; Gomperts, R.; Stratmann, R. E.; Yazyev, O.; Austin, A. J.; Cammi, R.; Pomelli, C.; Ochterski, J. W.; R. L. Martin, K. Morokuma, V. G. Zakrzewski, G. A. Voth, Salvador, P.; Dannenberg, J. J.; Dapprich, S.; Daniels, A. D.; Farkas, O.; Foresman, J. B.; Ortiz, J. V.; Cioslowski, J.; D. J. Fox, D. J. Gaussian 09, Revision A.02, Gaussian, Inc., Wallingford CT, 2009.

2. *Spartan'14* Wavefunction, Inc. Irvine, CA.

M062X/6-311+G** Optimized Structure and Energy of Cation **24**

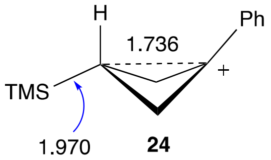

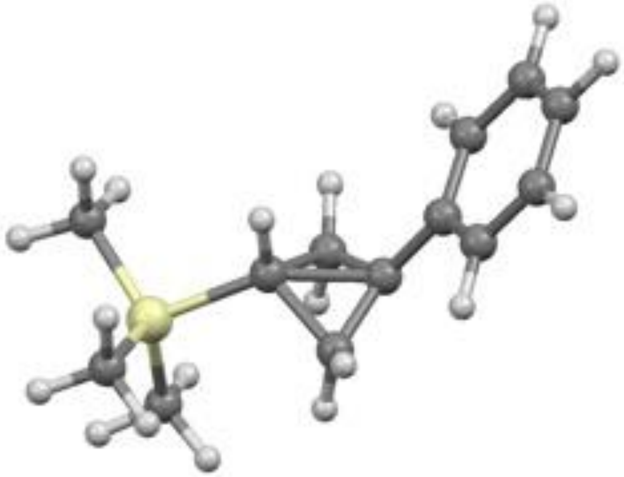

E(RM062X) = -795.9495326
Zero-point correction = 0.282375 au
No imaginary frequencies

Center Number	Atomic Number	Atomic Type	Coordinates (Angstroms) X	Y	Z
1	1	0	0.463960	1.907745	1.598640
2	6	0	0.656405	1.416046	2.544061
3	6	0	0.636476	-0.171308	2.611264
4	6	0	-0.469592	0.953347	3.336810
5	6	0	0.116752	-0.125344	4.112927
6	1	0	-0.524733	-0.920603	4.471888
7	1	0	0.943317	0.123242	4.772790
8	1	0	1.524561	1.788187	3.080711
9	6	0	-1.875324	1.063906	2.966243
10	1	0	-0.037857	-0.654034	1.906489
11	14	0	2.426082	-0.978809	2.454308
12	6	0	3.585105	-0.018279	3.554554
13	6	0	2.186109	-2.738498	3.013437
14	6	0	2.847560	-0.816058	0.648287
15	1	0	4.558302	-0.519089	3.533532
16	1	0	3.274065	0.010128	4.601434
17	1	0	3.751790	1.003754	3.207340
18	1	0	3.830012	-1.256340	0.456322
19	1	0	2.890170	0.229416	0.333772
20	1	0	2.125447	-1.338535	0.016651
21	1	0	3.121228	-3.294249	2.902027
22	1	0	1.428126	-3.250585	2.416110
23	1	0	1.895010	-2.796013	4.065027
24	6	0	-4.569552	1.327899	2.313253
25	6	0	-2.847628	0.334665	3.667368
26	6	0	-2.273931	1.950375	1.954233
27	6	0	-3.614311	2.074918	1.627541
28	6	0	-4.186777	0.464324	3.336865
29	1	0	-2.565116	-0.319517	4.482696
30	1	0	-1.544362	2.555608	1.430948
31	1	0	-3.917443	2.760769	0.846675
32	1	0	-4.933655	-0.098503	3.882204
33	1	0	-5.617662	1.430804	2.059147

M062X/6-311+G** Optimized Structure and Energy of Cation **27**

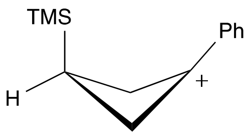

27

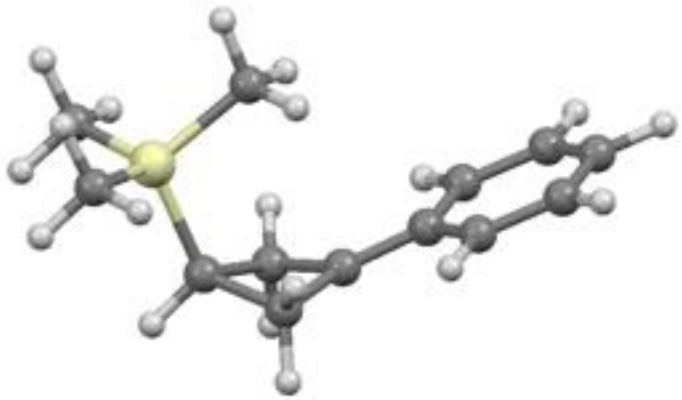

E(RM062X) = -795.9378959
Zero-point correction = 0.280682 au
No imaginary frequencies

Center Number	Atomic Number	Atomic Type	Coordinates (Angstroms) X	Y	Z
1	1	0	0.633899	1.855121	-0.936018
2	6	0	0.728437	1.708920	0.141422
3	6	0	1.144323	0.369704	0.623405
4	6	0	-0.533041	1.536594	1.061014
5	6	0	0.265992	0.415716	1.817206
6	1	0	-0.212919	-0.512894	2.132673
7	1	0	0.805748	0.845705	2.669877
8	1	0	1.391020	2.482250	0.549119
9	1	0	-0.740306	2.410889	1.678550
10	14	0	-2.099951	0.923355	0.155118
11	1	0	3.921363	-3.497329	-1.593939
12	6	0	1.853430	-0.671409	0.015740
13	6	0	-3.360471	0.451444	1.450897
14	6	0	-2.698880	2.306637	-0.948765
15	6	0	-1.583192	-0.574460	-0.860394
16	1	0	-0.827556	-0.334165	-1.614294
17	1	0	-2.452876	-0.961350	-1.398880
18	1	0	-1.205178	-1.394549	-0.242620
19	1	0	-3.004297	-0.356354	2.095552
20	1	0	-4.284906	0.108341	0.979735
21	1	0	-3.613576	1.303124	2.087218
22	1	0	-1.955320	2.583468	-1.700732
23	1	0	-2.940124	3.200965	-0.368841
24	1	0	-3.604884	2.007342	-1.481569
25	6	0	3.340902	-2.701508	-1.140091
26	6	0	2.496055	-0.459500	-1.230516
27	6	0	1.972364	-1.924580	0.668767
28	6	0	2.715066	-2.930543	0.090437
29	6	0	3.236355	-1.472081	-1.800319
30	1	0	2.404519	0.501177	-1.724204
31	1	0	1.481689	-2.080547	1.622663
32	1	0	2.816117	-3.890953	0.578988
33	1	0	3.733873	-1.323222	-2.749845

M062X/6-311+G** Optimized Structure and Energy of Transition State **28**

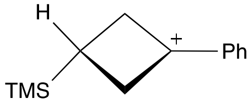

28

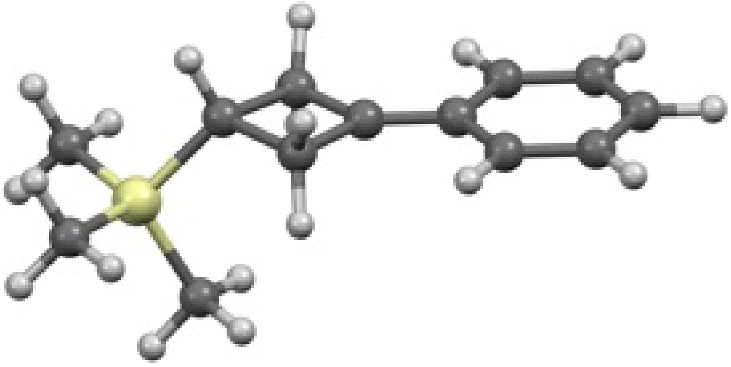

E(RM062X) = -795.9339923
Zero-point correction = 0.280551 au
One imaginary frequency at -33.97 cm^{-1}

Center Number	Atomic Number	Atomic Type	Coordinates (Angstroms) X	Y	Z
1	1	0	-0.127681	-0.068635	-0.164709
2	6	0	0.021315	-0.007263	0.918759
3	6	0	0.701505	-1.221598	1.626352
4	6	0	-1.211246	-0.265669	1.712313
5	6	0	-0.602932	-1.395539	2.466677
6	1	0	-1.148996	-2.339949	2.367832
7	1	0	-0.517547	-1.179766	3.540460
8	1	0	0.453524	0.979842	1.132475
9	6	0	-2.480710	0.312552	1.718942
10	1	0	0.841213	-2.054883	0.935329
11	14	0	2.318050	-0.882380	2.582692
12	6	0	1.998147	0.559267	3.746404
13	6	0	2.734723	-2.430180	3.546206
14	6	0	3.630508	-0.439476	1.326133
15	1	0	2.910375	0.776182	4.309243
16	1	0	1.219876	0.345920	4.484394
17	1	0	1.730271	1.479698	3.220204
18	1	0	4.582129	-0.230269	1.821319
19	1	0	3.359153	0.451693	0.753478
20	1	0	3.799596	-1.256602	0.620445
21	1	0	3.655080	-2.290657	4.118948
22	1	0	2.886771	-3.284921	2.882446
23	1	0	1.945532	-2.689849	4.257194
24	6	0	-4.999893	1.458736	1.730947
25	6	0	-3.480317	-0.179711	2.598398
26	6	0	-2.773378	1.392424	0.845327
27	6	0	-4.028501	1.959150	0.855657
28	6	0	-4.732060	0.394533	2.600363
29	1	0	-3.248866	-1.005655	3.261981
30	1	0	-2.004371	1.761933	0.175861
31	1	0	-4.266708	2.782785	0.195202
32	1	0	-5.504571	0.029947	3.264907
33	1	0	-5.987174	1.907528	1.735291

M062X/6-311+G** Optimized Structure and Energy of Cation **37**

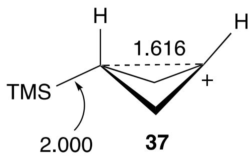

37

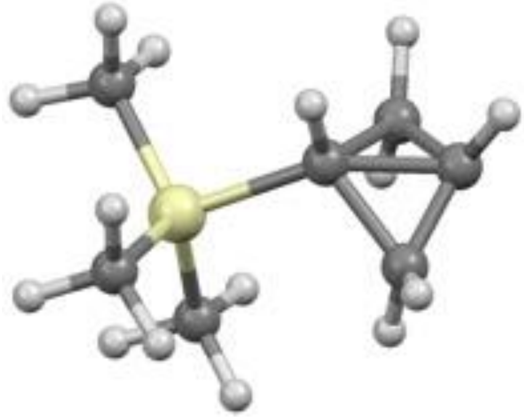

E(RM062X) = -564.9201042
Zero-point correction = 0.201629 au
No imaginary frequencies

Center Number	Atomic Number	Atomic Type	Coordinates (Angstroms) X	Y	Z
1	1	0	1.110896	0.968829	-1.087551
2	6	0	-0.634320	-0.879830	-0.328303
3	1	0	-1.326724	-1.585184	0.114867
4	6	0	0.419555	-0.359066	0.496999
5	1	0	-0.466209	-0.978178	-1.395538
6	6	0	-0.760384	0.662235	0.076927
7	1	0	-1.253145	0.914478	1.011849
8	6	0	0.815791	0.910069	-0.045079
9	1	0	0.480009	-0.575171	1.556497
10	1	0	1.274296	1.629342	0.622583
11	1	0	-1.965518	4.104548	-1.563300
12	14	0	-1.713494	1.676058	-1.359770
13	6	0	-3.474596	1.124237	-1.152230
14	6	0	-1.402045	3.447849	-0.894387
15	6	0	-0.986036	1.203700	-3.005231
16	1	0	0.080467	1.418259	-3.100526
17	1	0	-1.491098	1.812182	-3.763263
18	1	0	-1.165759	0.161391	-3.276766
19	1	0	-3.598207	0.061633	-1.374193
20	1	0	-4.113264	1.679206	-1.845431
21	1	0	-3.847133	1.315031	-0.143244
22	1	0	-0.346766	3.715229	-0.986982
23	1	0	-1.727801	3.664639	0.125594

M062X/6-311+G** Optimized Structure and Energy of Cation **47a**

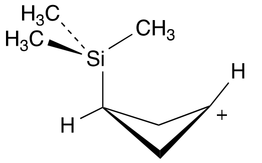

47a

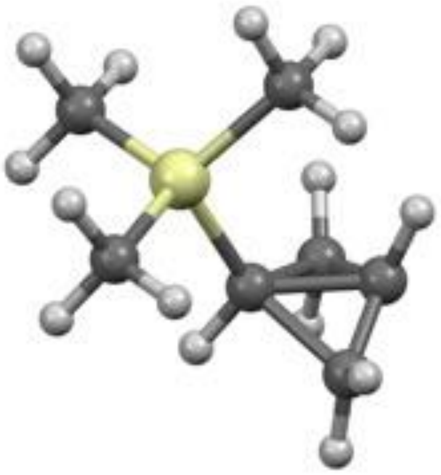

E(RM062X) = -564.9029481
Zero-point correction = 0.199966 au
No imaginary frequencies

Center Number	Atomic Number	Atomic Type	Coordinates (Angstroms) X	Y	Z
1	6	0	-1.156236	0.000065	1.162303
2	6	0	-1.459308	-1.185726	0.022351
3	1	0	-0.859216	-2.086473	0.071203
4	1	0	0.234849	0.002315	-0.629146
5	1	0	-2.530196	-1.318880	-0.069893
6	14	0	0.327066	-0.000156	2.415484
7	1	0	-2.142247	-0.000338	1.620115
8	6	0	-1.460556	1.187592	0.023855
9	6	0	-0.834118	0.001616	-0.440123
10	1	0	-0.861467	2.088927	0.074174
11	1	0	-2.531620	1.319635	-0.067920
12	6	0	0.110222	1.558184	3.412200
13	6	0	0.109419	-1.558367	3.412206
14	6	0	1.900024	-0.000618	1.408594
15	1	0	2.002175	-0.890935	0.781387
16	1	0	2.755885	-0.001203	2.089475
17	1	0	2.003057	0.889898	0.781819
18	1	0	0.195680	-2.457486	2.797238
19	1	0	-0.856609	-1.582438	3.922323
20	1	0	0.884428	-1.616703	4.181177
21	1	0	-0.855610	1.582526	3.922677
22	1	0	0.196510	2.457270	2.797188
23	1	0	0.885534	1.616327	4.180881

M062X/6-311+G** Optimized Structure and Energy of Cation **47b**

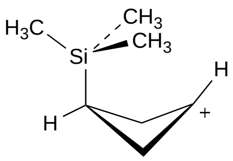

47b

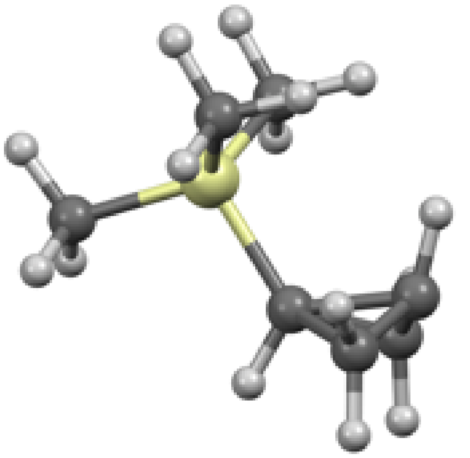

E(RM062X) = -564.9034249
Zero-point correction = 0.199807 au
No imaginary frequencies

Center Number	Atomic Number	Atomic Type	Coordinates (Angstroms) X	Y	Z
1	6	0	-1.167352	0.034046	1.154173
2	6	0	-2.314115	-1.179019	1.031160
3	1	0	-2.337892	-1.912504	1.827808
4	1	0	-0.405508	-1.910791	0.294300
5	1	0	-3.260320	-0.742869	0.735435
6	14	0	0.308385	0.013642	2.421046
7	1	0	-1.859109	0.870383	1.211283
8	6	0	-0.976222	0.027193	-0.506778
9	6	0	-1.227247	-1.221634	0.119736
10	1	0	0.021396	0.213506	-0.885232
11	1	0	-1.786365	0.585168	-0.959903
12	6	0	0.110439	1.550543	3.449027
13	6	0	0.107622	-1.564346	3.397815
14	6	0	1.859520	0.009321	1.382555
15	1	0	1.937880	-0.866146	0.731154
16	1	0	2.734167	-0.012841	2.038503
17	1	0	1.945633	0.909405	0.768480
18	1	0	0.160516	-2.463410	2.776677
19	1	0	-0.829821	-1.583542	3.959517
20	1	0	0.917597	-1.643771	4.128134
21	1	0	-0.838713	1.555735	3.990503
22	1	0	0.165127	2.455329	2.838712
23	1	0	0.910437	1.608718	4.191662

M062X/6-311+G** Optimized Structure and Energy of Transition State for Inversion of Cation **47** to Cation **37**

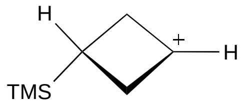

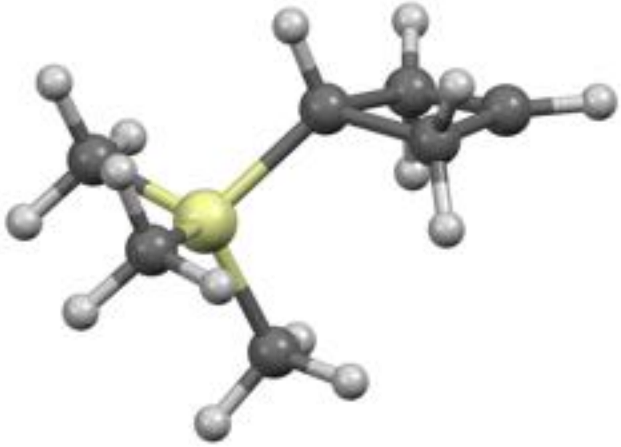

E(RM062X) = -564.8633430
Zero-point correction = 0.195005 au
One imaginary frequency at -227.79 cm^{-1}

Center Number	Atomic Number	Atomic Type	Coordinates (Angstroms) X	Y	Z
1	1	0	1.088498	0.949581	-0.922379
2	6	0	-0.779022	-0.736072	0.030412
3	1	0	-1.226755	-1.328498	0.847004
4	6	0	0.664678	-0.643868	0.191265
5	1	0	-1.092816	-1.253253	-0.890887
6	6	0	-0.864833	0.820548	0.104760
7	1	0	-1.246471	1.131395	1.079268
8	6	0	0.699514	0.793968	0.117667
9	1	0	1.439142	-1.396824	0.347804
10	1	0	1.364155	1.373745	0.764177
11	1	0	-2.033276	4.167055	-1.778124
12	14	0	-1.751901	1.757132	-1.317563
13	6	0	-3.521676	1.163770	-1.321164
14	6	0	-1.583263	3.582396	-0.971874
15	6	0	-0.859644	1.272794	-2.898102
16	1	0	0.142802	1.704784	-2.970681
17	1	0	-1.418235	1.654924	-3.757225
18	1	0	-0.783668	0.190128	-3.040313
19	1	0	-3.598992	0.093380	-1.530379
20	1	0	-4.092032	1.683545	-2.095182
21	1	0	-4.014301	1.360536	-0.365872
22	1	0	-0.536390	3.888333	-0.895682
23	1	0	-2.086994	3.861984	-0.043403

M062X/6-311+G** Optimized Structure and Energy of Cation **52**

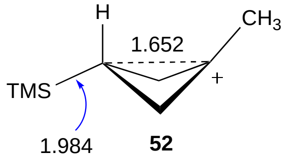

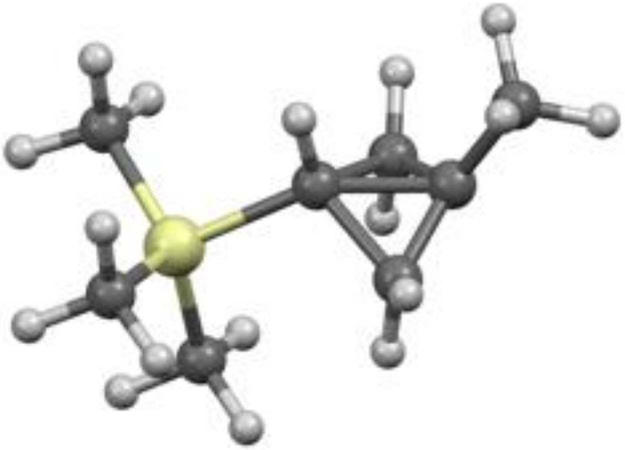

E(RM062X) = -604.2374265
Zero-point correction = 0.228894 au
One imaginary frequency at -23.62 cm^{-1}

Center Number	Atomic Number	Atomic Type	Coordinates (Angstroms) X	Y	Z
1	1	0	-0.013686	0.540255	0.296523
2	6	0	-0.074539	0.238571	1.336059
3	6	0	0.586536	-1.150417	1.772033
4	6	0	-1.022972	-0.790480	1.678520
5	6	0	-0.529813	-1.393379	2.890569
6	1	0	-0.836840	-2.410336	3.107096
7	1	0	-0.329773	-0.766651	3.753768
8	1	0	0.164785	1.006047	2.065184
9	1	0	0.698127	-1.912769	1.004372
10	6	0	-1.904448	-1.481823	0.694569
11	14	0	2.370736	-0.867188	2.591809
12	6	0	2.215510	0.434282	3.914253
13	6	0	2.806255	-2.543056	3.268229
14	6	0	3.423666	-0.326004	1.158570
15	1	0	4.458021	-0.198469	1.490214
16	1	0	3.092529	0.630614	0.747585
17	1	0	3.428194	-1.066254	0.355032
18	1	0	3.812447	-2.516339	3.696023
19	1	0	2.803762	-3.309139	2.489271
20	1	0	2.122375	-2.852721	4.062014
21	1	0	3.211423	0.571384	4.348690
22	1	0	1.557100	0.147716	4.737063
23	1	0	1.907511	1.411048	3.535166
24	1	0	-1.948990	-2.555372	0.879440
25	1	0	-1.594256	-1.283889	-0.331721
26	1	0	-2.911973	-1.075492	0.826045

M062X/6-311+G** Optimized Structure and Energy of Cation **54**

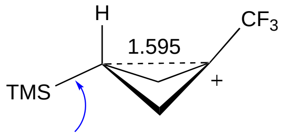

54

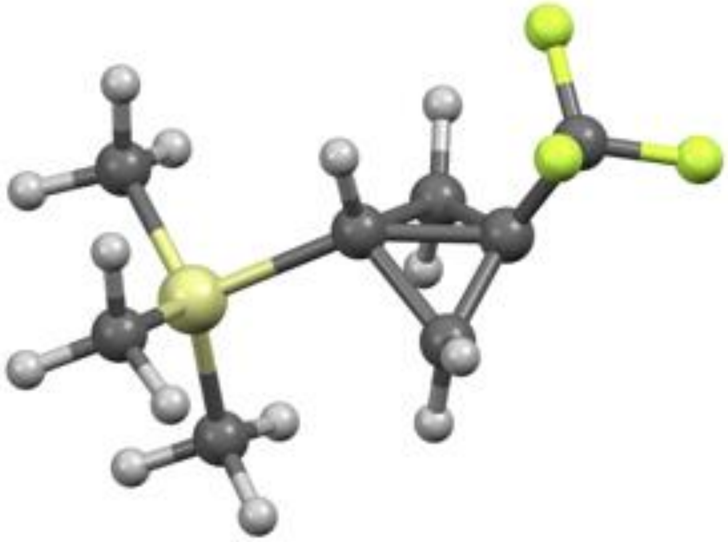

E(RM062X) = -901.9562706
Zero-point correction = 0.206631 au
No imaginary frequencies

Center Number	Atomic Number	Atomic Type	Coordinates (Angstroms) X	Y	Z
1	6	0	1.172771	-0.057034	0.669062
2	6	0	0.346628	1.093866	0.916971
3	6	0	0.336978	-1.226201	0.682252
4	6	0	-0.197274	0.032328	-0.143352
5	1	0	0.088805	0.135746	-1.188656
6	1	0	0.670981	-2.090470	0.119917
7	1	0	-0.279452	-1.419037	1.553444
8	1	0	0.692216	2.049774	0.540789
9	1	0	-0.267723	1.111469	1.810293
10	14	0	-2.220640	0.018407	-0.118888
11	6	0	-2.622235	-1.560478	-1.006069
12	1	0	-2.156862	-1.603095	-1.993545
13	1	0	-3.704468	-1.623793	-1.154306
14	1	0	-2.319438	-2.443180	-0.438175
15	6	0	-2.629521	1.565016	-1.057977
16	1	0	-2.265193	2.461534	-0.551200
17	1	0	-3.716562	1.656426	-1.143761
18	1	0	-2.223531	1.546701	-2.071879
19	6	0	-2.770249	0.048818	1.655577
20	1	0	-2.569935	0.999869	2.153372
21	1	0	-2.371070	-0.764644	2.265375
22	1	0	-3.858438	-0.077507	1.652444
23	6	0	2.463313	0.017937	-0.131981
24	9	0	2.538871	1.168849	-0.794238
25	9	0	2.529705	-0.978472	-1.010591
26	9	0	3.483567	-0.070680	0.703770

M062X/6-311+G** Optimized Structure and Energy of 3-Trimethylsilyl-1-cyanocyclobutyl Cation, **74b**

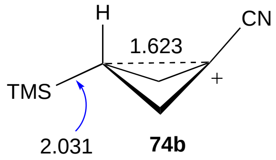

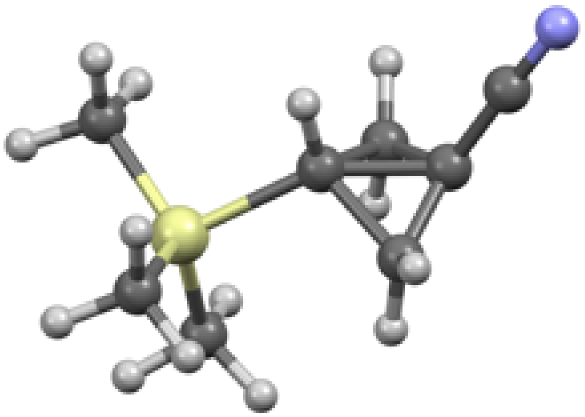

E(RM062X) = -657.1326325
Zero-point correction = 0.199579 au
No imaginary frequencies

Center Number	Atomic Number	Atomic Type	Coordinates (Angstroms) X	Y	Z
1	14	0	0.067399	0.022490	-1.632635
2	6	0	0.320286	0.026839	0.382807
3	6	0	-0.726369	-0.956591	1.068678
4	6	0	-0.351201	1.335082	0.994751
5	6	0	-0.324835	0.181672	1.864441
6	1	0	-1.688843	-0.915675	0.569502
7	6	0	0.546127	0.076710	2.998407
8	1	0	1.380203	-0.140216	0.555945
9	1	0	0.289332	2.171236	1.250359
10	1	0	-1.280548	1.572893	0.487625
11	1	0	-0.384091	-1.934633	1.386487
12	7	0	1.214839	-0.001538	3.928309
13	6	0	1.006118	1.535741	-2.151599
14	6	0	0.851658	-1.589231	-2.110843
15	6	0	-1.756774	0.106906	-1.970213
16	1	0	0.325870	-2.445220	-1.681791
17	1	0	1.901417	-1.637197	-1.812982
18	1	0	0.816506	-1.695763	-3.199440
19	1	0	2.039517	1.516542	-1.797799
20	1	0	0.532960	2.456585	-1.803037
21	1	0	1.038035	1.579535	-3.244615
22	1	0	-2.290879	-0.802924	-1.688519
23	1	0	-1.873951	0.212948	-3.054438
24	1	0	-2.257572	0.969112	-1.524409

M062X/6-311+G** Optimized Structure and Energy of Cation **74c**

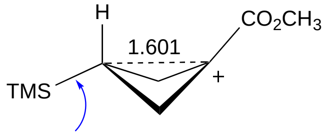

74c

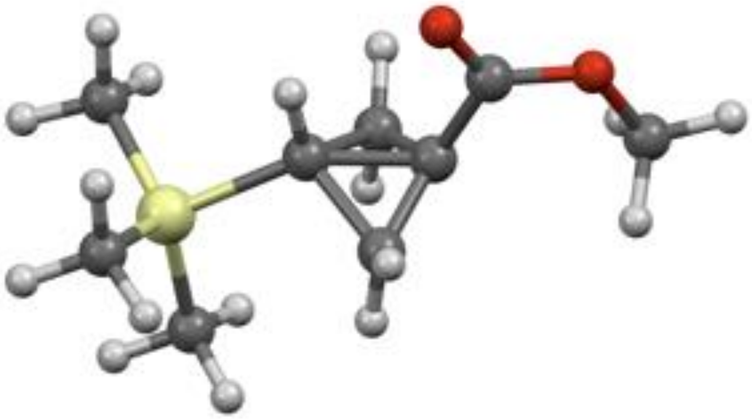

E(RM062X) = -792.7590508
Zero-point correction = 0.244226 au
No imaginary frequencies

Center Number	Atomic Number	Atomic Type	Coordinates (Angstroms) X	Y	Z
1	6	0	-0.278170	0.272264	-0.167135
2	6	0	0.438216	-1.097263	-0.556390
3	6	0	0.375823	0.323785	1.284632
4	6	0	1.214474	-0.161796	0.217477
5	1	0	0.607828	1.301529	1.693288
6	1	0	-0.096348	-0.384970	1.957498
7	1	0	-0.026599	-1.944777	-0.061697
8	14	0	-2.259385	-0.009856	-0.018087
9	1	0	-0.124217	1.131070	-0.818557
10	1	0	0.715201	-1.239569	-1.595568
11	6	0	-2.744556	-0.415485	-1.763980
12	1	0	-2.343696	-1.379201	-2.086993
13	1	0	-2.413639	0.352993	-2.466159
14	1	0	-3.834552	-0.474670	-1.833881
15	6	0	-2.853995	1.638705	0.592967
16	1	0	-2.476258	1.863493	1.593076
17	1	0	-3.946433	1.633403	0.648913
18	1	0	-2.564661	2.449807	-0.079034
19	6	0	-2.561837	-1.398083	1.182616
20	1	0	-2.273388	-1.155422	2.207504
21	1	0	-2.100802	-2.345590	0.895644
22	1	0	-3.642658	-1.575381	1.199611
23	6	0	2.325786	0.702972	-0.387492
24	8	0	2.071243	1.705894	-0.978706
25	8	0	3.551511	0.260784	-0.193813
26	6	0	3.799085	-0.948240	0.544246
27	1	0	3.369271	-1.808888	0.029770
28	1	0	3.412728	-0.866928	1.561588
29	1	0	4.880033	-1.044937	0.573494

M062X/6-311+G** Optimized Structure and Energy of Cation **74d**

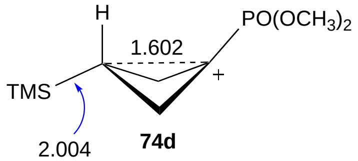

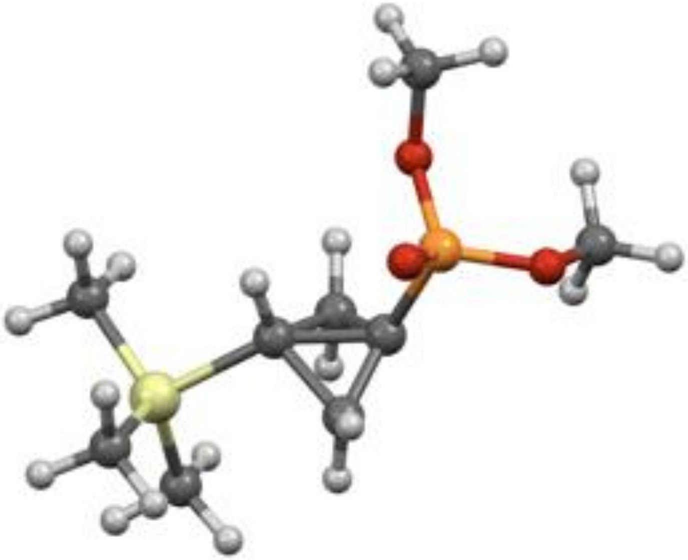

E(RM062X) = -1211.1913780
Zero-point correction = 0.283868 au
No imaginary frequencies

Center Number	Atomic Number	Atomic Type	Coordinates (Angstroms) X	Y	Z
1	6	0	0.335235	-0.937702	-0.115669
2	6	0	-0.675661	-1.464921	0.767882
3	6	0	-0.340873	-0.407217	-1.275511
4	6	0	-0.955164	-0.023441	0.143109
5	1	0	-0.585847	0.854269	0.670257
6	1	0	0.165248	0.373068	-1.831888
7	1	0	-1.017621	-1.048595	-1.831126
8	1	0	-0.433383	-1.505120	1.825118
9	1	0	-1.380876	-2.198977	0.392179
10	14	0	-2.937288	0.231843	-0.010280
11	6	0	-3.058383	1.886361	-0.844750
12	1	0	-2.570783	2.671971	-0.263436
13	1	0	-4.110796	2.164445	-0.952435
14	1	0	-2.619498	1.869575	-1.845133
15	6	0	-3.496081	0.215684	1.759617
16	1	0	-3.372671	-0.769101	2.216114
17	1	0	-4.558559	0.470580	1.808857
18	1	0	-2.954224	0.946733	2.363895
19	6	0	-3.647595	-1.153065	-1.028934
20	1	0	-3.460084	-2.148258	-0.620484
21	1	0	-3.326931	-1.129427	-2.072430
22	1	0	-4.734846	-1.020637	-1.033128
23	15	0	1.897156	-0.296942	0.605138
24	8	0	1.722117	0.009142	2.025229
25	8	0	2.167148	0.913745	-0.391037
26	8	0	2.956143	-1.419527	0.284281
27	6	0	3.108421	1.955089	-0.020115
28	1	0	4.122763	1.555307	-0.032971
29	1	0	2.864100	2.334585	0.971657
30	1	0	3.002341	2.734592	-0.768475
31	6	0	3.421310	-1.721891	-1.046216
32	1	0	4.209356	-2.459066	-0.924882
33	1	0	3.811712	-0.822567	-1.522492
34	1	0	2.609208	-2.143525	-1.641956

B3LYP/6-31G* Optimized Structure and NMR Shifts of **17**

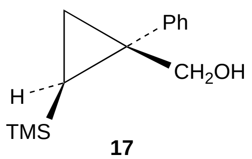

17

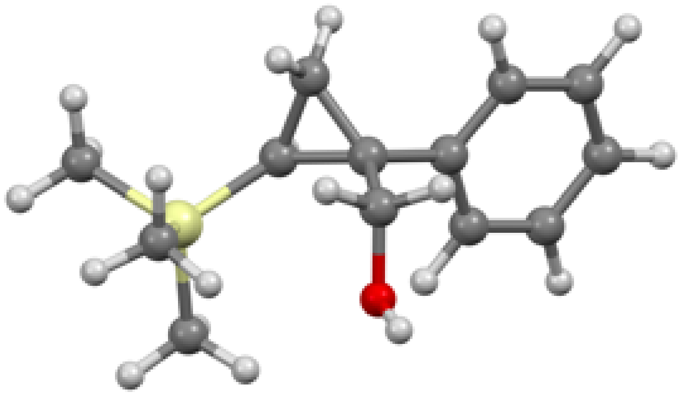

Coordinates (Angstroms)

H	0.330211	-2.352992	1.167969
C	-0.267625	-1.862993	0.403822
C	-0.768319	-0.454599	0.681700
C	0.343514	-0.699541	-0.338607
H	-0.910040	-2.543248	-0.152436
H	-0.366847	-0.053267	1.615312
C	1.740365	-0.253868	0.019086
C	4.362433	0.593552	0.598077
C	2.784759	-1.180435	0.137152
C	2.032551	1.107330	0.188651
C	3.331047	1.527477	0.477213
C	4.085524	-0.763241	0.425696
H	2.574602	-2.239145	0.003304
H	1.233553	1.835747	0.088276
H	3.537878	2.586683	0.609361
H	4.881015	-1.498680	0.517046
H	5.373563	0.921238	0.825689
C	0.024865	-0.581032	-1.817938
H	-0.866986	-1.175762	-2.058394
H	0.866573	-0.987933	-2.398557
O	-0.188551	0.795646	-2.142944
H	-0.178508	0.880273	-3.108388
Si	-2.527444	0.179950	0.407410
C	-3.558930	-0.427557	1.880485
H	-4.590523	-0.058776	1.821763
H	-3.139920	-0.085313	2.834674
H	-3.599475	-1.523166	1.912893
C	-2.492157	2.070944	0.392252
H	-3.498088	2.490454	0.267294
H	-1.866685	2.425841	-0.434191
H	-2.079731	2.471327	1.326471
C	-3.331472	-0.451785	-1.188477
H	-4.390676	-0.166309	-1.207605
H	-3.285669	-1.543895	-1.276150
H	-2.850965	-0.013055	-2.069191

NMR shifts (ppm)

Atom	Isotropic	Rel. Shift
H1	31.0659	1.12
C1	173.0564	16.57
C2	175.3786	14.25
C3	155.0499	34.58
H2	31.4657	0.72
H3	32.0929	0.09
C4	49.2770	140.35
C5	70.1136	119.51
C6	68.5570	121.07
C7	63.1759	126.45
C8	68.7027	120.92
C9	69.0199	120.61
H5	25.0589	7.13
H7	24.4492	7.73
H8	25.0079	7.18
H9	25.0582	7.13
H10	25.0972	7.09
C10	119.3443	70.28
H11	28.7654	3.42
H12	28.1888	4.00
O1	294.6181	
H6	31.9855	0.20
Si1	407.6115	6.07
C11	189.1555	0.47
H13	32.1806	0.00
H15	32.0755	0.11
H16	32.0119	0.17
C12	189.9673	-0.34
H14	32.1131	0.07
H17	31.3417	0.84
H18	31.9491	0.24
C13	191.3329	-1.71
H4	32.2723	-0.09
H19	32.2053	-0.02
H20	32.0495	0.13

Calculated (Rel. Shift)$_{average}$ = 0.16 ppm
Observed TMS Shift in 17 = 0.14 ppm

B3LYP/6-31G* Optimized Structure and NMR Shifts of **18**

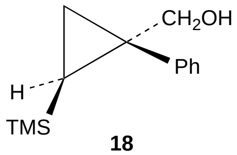

18

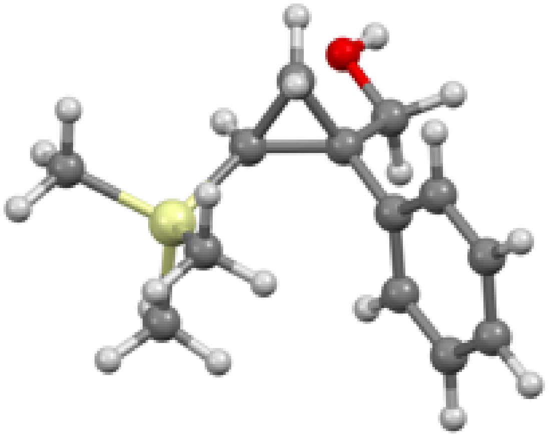

Coordinates (Angstroms)

H	-0.459603	-0.670874	-2.193903
C	-0.653335	-1.378395	-1.391662
C	0.194768	-1.287485	-0.156653
C	-1.264102	-0.841318	-0.098709
H	-0.958954	-2.366952	-1.721373
H	-1.892334	-1.582537	0.397735
Si	-1.972175	0.902837	0.033447
C	-3.789548	0.786049	-0.500659
H	-4.357248	0.108556	0.148827
H	-3.880623	0.412571	-1.527509
H	-4.275349	1.768813	-0.459733
C	-1.921562	1.533741	1.822939
H	-2.324024	0.790069	2.521853
H	-2.526400	2.443231	1.926506
H	-0.902361	1.778190	2.142592
C	-1.089237	2.141178	-1.095925
H	-1.128133	1.833418	-2.147780
H	-0.036399	2.272392	-0.825472
H	-1.577286	3.121821	-1.026896
C	1.331987	-0.301492	-0.076593
C	3.527201	1.454899	0.130078
C	2.139966	-0.026589	-1.187981
C	1.655735	0.308443	1.145925
C	2.739354	1.180533	1.250232
C	3.224192	0.847699	-1.089200
H	1.913566	-0.500567	-2.139297
H	1.043708	0.100035	2.019970
H	2.967584	1.647356	2.205065
H	3.833956	1.051898	-1.965763
H	4.370338	2.136291	0.207721
C	0.557925	-2.596124	0.546417
H	0.801556	-2.385445	1.600606
H	1.467721	-3.004676	0.075756
O	-0.519291	-3.521565	0.454040
H	-0.234988	-4.347615	0.871254

NMR shifts (ppm)

Atom	Isotropic	Rel. Shift
H1	31.3902	0.79
C1	178.1822	11.44
C2	156.7543	32.87
C3	179.1350	10.49
H2	30.7012	1.48
H6	31.3296	0.85
Si1	408.0626	5.62
C4	188.8410	0.79
H7	32.0571	0.13
H9	32.0218	0.16
H10	32.3201	-0.14
C5	190.1843	-0.56
H8	32.0229	0.16
H11	32.2949	-0.11
H12	32.0330	0.15
C6	192.8797	-3.25
H5	32.6855	-0.50
H13	33.5699	-1.39
H14	32.8008	-0.62
C7	52.5000	137.13
C8	69.8573	119.77
C9	66.2786	123.35
C10	65.2412	124.39
C11	68.7862	120.84
C12	68.0000	121.63
H3	24.9722	7.21
H15	24.7587	7.43
H16	25.0152	7.17
H17	25.0059	7.18
H18	25.0781	7.11
C13	124.4177	65.21
H19	28.1768	4.01
H20	8.3884	3.80
O1	298.9423	
H4	32.1347	0.05

Calculated (Rel. Shift)$_{average}$ = -0.23 ppm
Observed TMS Shift in 18 = -0.30 ppm
